# Functional architecture of reward learning in mushroom body extrinsic neurons of larval *Drosophila*

**DOI:** 10.1038/s41467-018-03130-1

**Published:** 2018-03-16

**Authors:** Timo Saumweber, Astrid Rohwedder, Michael Schleyer, Katharina Eichler, Yi-chun Chen, Yoshinori Aso, Albert Cardona, Claire Eschbach, Oliver Kobler, Anne Voigt, Archana Durairaja, Nino Mancini, Marta Zlatic, James W. Truman, Andreas S. Thum, Bertram Gerber

**Affiliations:** 1 Department of Genetics, Leibniz Institute for Neurobiology (LIN), Brenneckestr. 6, 39118 Magdeburg, Germany; 2 Institute for Biology, University of Konstanz, Universitätsstraße 10, 78464 Konstanz, Germany; 3 HHMI Janelia Research Campus, Helix Drive 19700, Ashburn, VA 20147 USA; 4 Special Lab Electron and Laserscanning Microscopy and Combinatorial Neuro Imaging Core Facility, Leibniz Institute for Neurobiology (LIN), Brenneckestr. 6, 39118 Magdeburg, Germany; 5 Friday Harbor Laboratories, University of Washington, Friday Harbor, WA 98250 USA; 6 Zukunftskolleg, University of Konstanz, Universitätsstraße 10, 78464 Konstanz, Germany; 7 Institute for Biology, University of Leipzig, Talstraße 33, 04103 Leipzig, Germany; 8 Center for Behavioral Brain Sciences (CBBS), Universitätsplatz 2, 39106 Magdeburg, Germany; 9 Institute for Biology, Otto von Guericke University Magdeburg, Universitätsplatz 2, 39106 Magdeburg, Germany

## Abstract

The brain adaptively integrates present sensory input, past experience, and options for future action. The insect mushroom body exemplifies how a central brain structure brings about such integration. Here we use a combination of systematic single-cell labeling, connectomics, transgenic silencing, and activation experiments to study the mushroom body at single-cell resolution, focusing on the behavioral architecture of its input and output neurons (MBINs and MBONs), and of the mushroom body intrinsic APL neuron. Our results reveal the identity and morphology of almost all of these 44 neurons in stage 3 *Drosophila* larvae. Upon an initial screen, functional analyses focusing on the mushroom body medial lobe uncover sparse and specific functions of its dopaminergic MBINs, its MBONs, and of the GABAergic APL neuron across three behavioral tasks, namely odor preference, taste preference, and associative learning between odor and taste. Our results thus provide a cellular-resolution study case of how brains organize behavior.

## Abstract (web summary)

The mushroom body of *Drosophila* integrates sensory information with past experience to guide behaviour. Here, the authors provide an atlas of the input and output neurons of the stage 3 larval mushroom body at the single-cell level, and analyse their function in learned and innate behaviours.

## Introduction

The insect mushroom body is an intensely studied example of a central brain structure that integrates present sensory input, past experience, and options for future behavior^1–6^. We use larval *Drosophila melanogaster* to study these processes at single-cell resolution. Our focus is on the mushroom body input (MBINs) and output neurons (MBONs), and their role in the association of odor with taste reward as a biologically meaningful learning process. Combined with the connectome of the larval mushroom body^7^, this provides a framework for understanding how a central brain structure is functionally organized.

**Fig. 1:**
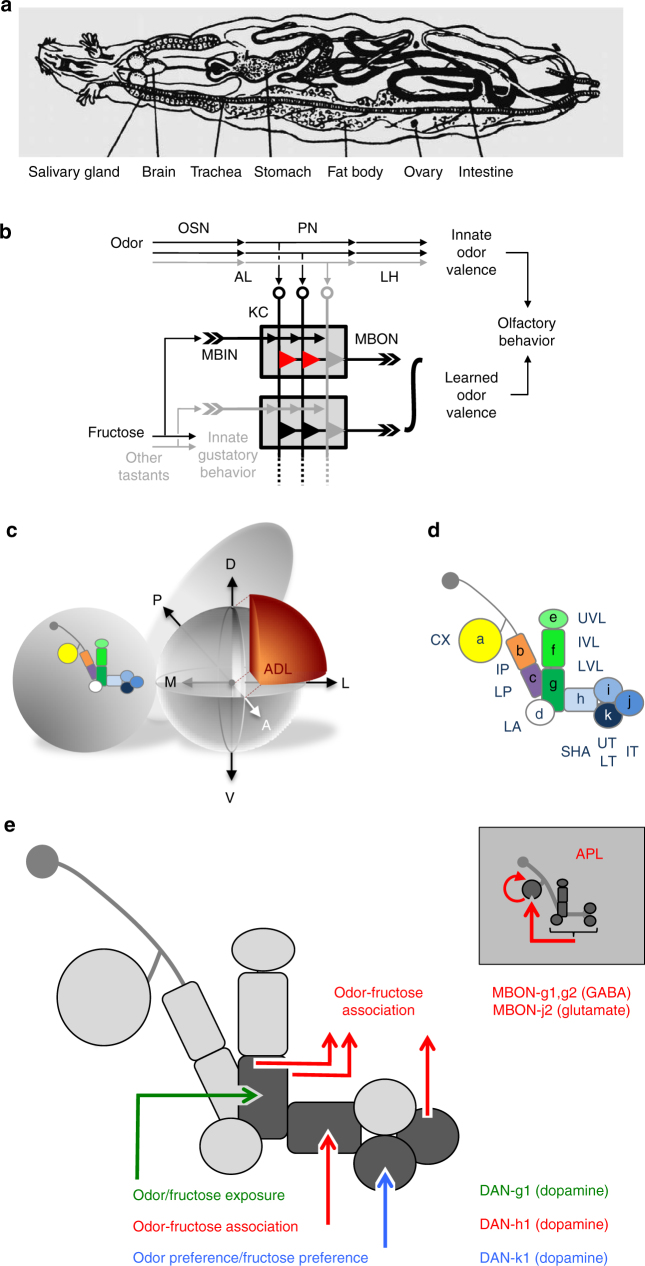
Overview of anatomical relationships, and of the requirement of MBINs and MBONs across behavioral tasks. **a** Body plan of stage 3 *Drosophila* larvae^102^ (modified with permission from ref. ^102^); brain refers to the ventral nerve cord plus the two brain hemispheres toward the left. Also see Supplementary Movies 1-5. **b** Simplified diagram of the olfactory and gustatory pathways, and of the organization of innate and learned olfactory behavior, as well as of innate gustatory behavior in the larva. AL antennal lobe, KC Kenyon cells of the mushroom body, LH lateral horn, MBIN mushroom body input neurons, MBON mushroom body output neurons, OSN olfactory sensory neurons, PN olfactory projection neurons. The red triangles indicate KC output synapses that are modulated by the joint presentation of odor and fructose; gray and black triangles indicate silent and active synapses, respectively. The gray boxes indicate mushroom body compartments. The integral sign implies that learned odor valence can be based on an integration of MBON activity from multiple compartments. Circuitry within the AL and the APL neuron are not displayed. Also, circuit motifs newly discovered^7^, namely KC-KC connections as well as KC > DAN and MBIN > MBON connections, are not included. **c** Schematic of the location and orientation of the mushroom body within the larval nervous system. The mushroom body is only shown in one hemisphere. A anterior, D dorsal, P posterior, L lateral, M medial, V ventral. **d** Organization of the larval mushroom body in 11 compartments. CX calyx; IP and LP intermediate and lower peduncle; LA lateral appendix; UVL, IVL, and LVL upper, intermediate, and lower vertical lobe; SHA, UT, IT, LT shaft as well as upper, intermediate, and lower toe of the medial lobe. Single-letter synonyms of compartment names are given as “a–k”; these letters are used to indicate compartment innervation by the MBEs in Fig. 2. **e** Summary of the requirement of MBINs, MBONs, and of the APL neuron across behavioral tasks, based on the data shown in Figs. 2–4, and Supplementary Table 1

The larval olfactory system is organized like that of adult insects, yet at much reduced cell numbers^8–13^ (Fig. 1a, b). Its 21 olfactory sensory neurons define the range of odors detectable for the larva. Each olfactory sensory neuron targets just one glomerulus in the antennal lobe. The 21 antennal lobe glomeruli are laterally connected by 14 interneurons^8, 14^. A total of 34 olfactory projection neurons then connect the antennal lobe with the lateral horn and the mushroom body. Depending on the odorant receptors expressed and the connectivity within this system, olfactory stimuli can thus be coded combinatorially across these pathways (but also see ref. ^15^). Processing along the lateral horn pathway is largely sufficient for olfactory behavior in experimentally naïve animals, whereas learned olfactory behavior requires the mushroom body loop^16–18^ (but also see ref. ^19^). The olfactory pathways remain mostly ipsilateral^8^, are bilaterally symmetrical, and are largely stereotyped. A notable exception to stereotypy is that the olfactory projection neurons connect in a predominantly random fashion to ~800 mushroom body intrinsic Kenyon cells (KCs)^7, 20, 21^. The high input resistance of the KCs and GABAergic gain control result in a sparse combinatorial code across the mushroom body^22^. This architecture of the olfactory system resembles that of mammals^3^.

Despite significant advances^23–31^, the taste system of the larva is less well understood. The ~80 larval gustatory sensory neurons are located in three external and four pharyngeal sense organs. However, a comprehensive picture of taste coding in the brain is just emerging^9, 11, 32, 33^. What is clear is that gustatory sensory neurons project to the subesophageal zone. From the subesophageal zone taste information is passed on for steering innate gustatory behavior (throughout this study, “innate” is used in the sense of “experimentally naïve”), and toward the brain. These brain projections connect, via an unknown number of synaptic steps, to MBINs. There follows a simplified account of how associative learning comes about at the interface of MBINs, KCs, and the MBONs (Fig. 1b).

The majority of MBINs are either dopaminergic or octopaminergic/tyraminergic (DANs and OANs, respectively) and are necessary and sufficient for reinforcement signaling in insects^34–42^. Optogenetic activation of OANs in larval *Drosophila* is sufficient as an internal reward signal^36^, as is activation of DANs from the pPAM cluster^42^. In turn, activation of DANs from other clusters that innervate separate mushroom body compartments is sufficient as a punishment^36^. Accordingly, a knockdown of the DopR1 dopamine receptor in the KCs impairs both appetitive and aversive learning^37^ (in adult *Drosophila*^43^). During odor-reward training, for example, an odor-specific subset of KCs is activated via the projection neurons. At the same time, a subset of MBINs is activated by the reward. Locally within the MBINs’ target compartments, therefore, most if not all KCs receive an aminergic internal reward signal (for punishment learning and potentially also for non-associative forms of learning different MBINs and their target compartments are involved). As has been shown in adult *Drosophila*, coincident input of odor and the aminergic reinforcement signal is detected within the odor-activated KCs^44–46^, resulting in modification of their output synapses. In this way the level of activity in the MBONs will reflect what has been learned about an odor^47–52^. Such learned-valence signals from the MBONs are then summed up with innate odor valence information delivered toward the lateral horn by the projection neurons^49, 53^ (Fig. 1b). The mushroom bodies thus are an example of a central brain structure integrating present sensory input (projection neurons, MBINs) and past experience associated with such input (the modified KC-to-MBON presynaptic terminals) to provide instruction for future behavior (via the MBONs).

For both larval and adult *Drosophila* the exact division of labor among individual MBINs remains to be elucidated^54^. Elucidation is required specifically in order to understand how separate memories for distinct kinds of reward can be established^55^. Likewise, the way in which MBONs affect learned behavior has only recently begun to be understood in adults^47–49, 51, 52, 56^ and remains clouded in the case of larvae. Here these issues are addressed at the single-cell level, in the larva.

## Results

### Atlas of mushroom body extrinsic neurons in stage 3 larvae

**Fig. 2:**
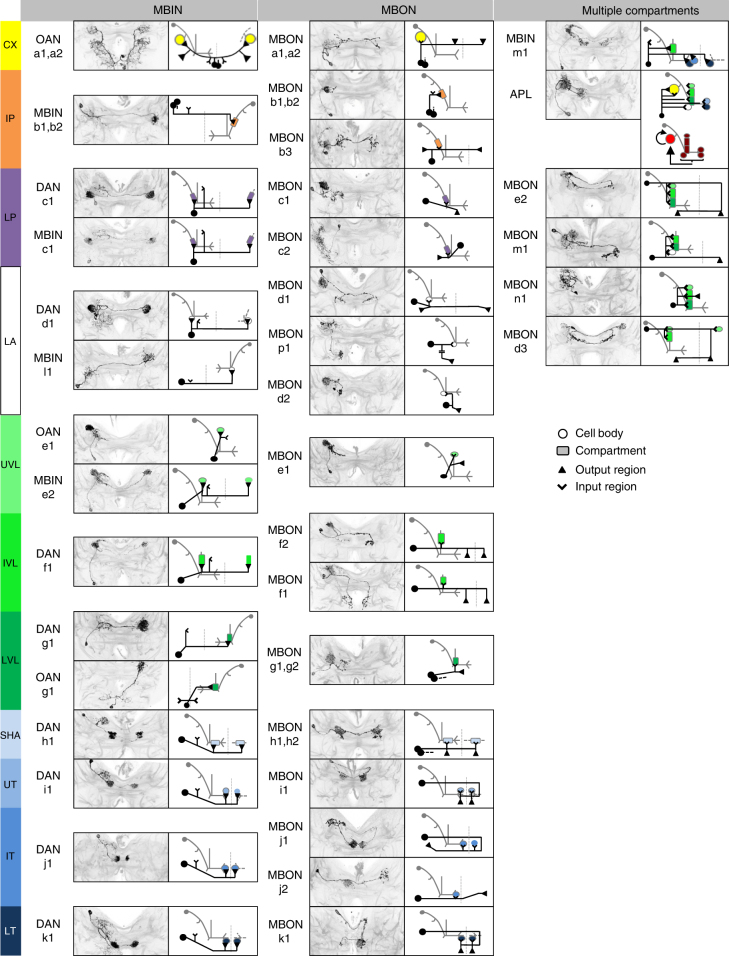
Atlas of the MBE neurons. Identification of the MBE neurons by compartment innervation, input and output regions, and cell body location. The MBE of only one hemisphere is shown (for the segmentally homologous, unpaired OAN-a1 and OAN-a2 neurons the cell bodies are located at the midline). For MBE pairs, the second neuron of the pair is indicated by its cell body and stippled primary neurite. Anatomical panels show z-projections of those parts of the larval brain that include the respective MBE. MBEs are visible based on antibody staining against the flp-out effectors; anti-neuroglian staining in gray reveals the local brain structure. Note that the nomenclature for the MBIN-l1 and MBON-p1 neuron is based on their compartment innervation in the stage 1 larva (Supplementary Fig. 1). For more details, see Supplementary Table 1

We selected 877 Gal4 driver strains with expression in the central nervous system of stage 3 *Drosophila* larvae^57^(http://flweb.janelia.org/cgi-bin/flew.cgi) and inspected them for coverage of mushroom body extrinsic neurons (MBEs). Systematic multicolor flp-out^57, 58^ revealed the identity and morphology of 11 mushroom body compartments and 44 individual MBE neurons (Figs. 1c, d and  2, Supplementary Table 1-3). We then established a subcollection of 102 Gal4 strains thus known to include MBE neurons in their expression pattern for functional analysis, and for narrowing down expression patterns by intersectional strategies^59, 60^ (Supplementary Table 4). A recent electron microscope reconstruction of a stage 1 larval brain^7^ has revealed three MBE neurons that have not yet been found in stage 3 larvae; in turn, six MBE neurons were identified that are present in stage 3 larvae but not in stage 1 (Supplementary Fig. 1, Supplementary Fig. 2). These differences suggest that our stage 3 MBE neuron atlas is about 95% complete, and that MBE neurons are incorporated into the system as the larvae grow and develop. Still, the behavioral faculties of stage 1 larvae qualitatively match those of stage 3 larvae across multiple assays, including those for odor preference, taste preference and odor-taste associative learning^61^.

Most MBE neurons are present as one cell per hemisphere, with mirror-image symmetry of the cells on the left and right side of the brain (pairs). Exceptions are the OAN-a1 and OAN-a2 neurons, with unpaired cell bodies located at the midline in the maxillary and mandibular segment, respectively (Supplementary Fig. 3); both these cells have bilaterally symmetrical morphology. In five cases MBE neurons are present as double pairs, i.e., with two cells per hemisphere (Supplementary Fig. 3, Supplementary Table 1).

The arborizations of the MBE neurons define 11 non-overlapping compartments of the mushroom body (Figs. 1d and 2, Supplementary Fig. 4). The majority of MBE neurons innervate a single compartment per hemisphere (38 out of 44) and in 14 of these 38 cases they also innervate the same compartment on the contralateral side (Fig. 2; for DAN-i1 as an example we provide more detail below). We have designated these neurons single-compartment MBE neurons. The six MBEs that innervate multiple compartments are called multiple-compartment MBE neurons.

The innervation by the MBE neurons intersects the axonal projections of the KCs. Within each compartment the MBE neurons can thus relate to the coding space encompassed across the KCs (for DAN-i1 and the anterior paired lateral (APL) neuron as examples we provide more detail below). In most cases the morphology of the MBE neurons allowed regions rich in presynaptic boutons to be distinguished from regions dominated by spine-like postsynaptic structures; electron microscopy verified these interpretations^7^. The MBE neurons were accordingly classified as MBINs or MBONs. The compartments are in register for MBINs and MBONs (Fig. 2). The APL neuron is the only MBE that communicates almost exclusively within the mushroom body and is therefore labeled as MBIN/MBON.

All MBINs except OAN-e1 provide inter-hemispheric cross talk; the same is observed for more than half of the MBONs (12/21 single-compartment MBONs and 3/4 multiple-compartment MBONs; Supplementary Table 1). This is striking, because little inter-hemispheric integration is otherwise seen along olfactory ascending pathways in the larva^7, 8, 62^.

With the exception of MBIN-m1 all MBINs are classified as being of the single-compartment type, whereas the MBONs feature four cases classified as multiple-compartment type. This suggests that mushroom body output is more integrated across compartments than mushroom body input is.

### Specific and sparse requirement of MBEs across behaviors

For a pre-screen of the function of the MBE neurons, the above-mentioned collection of 102 MBE-covering Gal4 driver strains was crossed to *UAS*-*shi*^*ts*^ (Supplementary Table 2 and Supplementary Table 3; four MBE neurons were not covered by this collection). At restrictive temperature, and thus with an acute block of synaptic output, the offspring were assayed in an odor-fructose (FRU) reward association task. The larvae received the odor and a FRU reward either in a paired manner or, in independent groups of animals, in an unpaired way. Then, the animals were tested for their preference for the odor. From the difference in preference between the paired-trained versus the unpaired-trained groups an associative performance index (PI) was calculated. Combining the data from the strains that cover a given MBE neuron, and taking into account a previous report^42^, this identified the MBEs innervating the medial lobe compartments, the lower vertical lobe compartment, and the APL neuron as candidates for being required in the odor-FRU association (Supplementary Fig. 5).

**Fig. 3:**
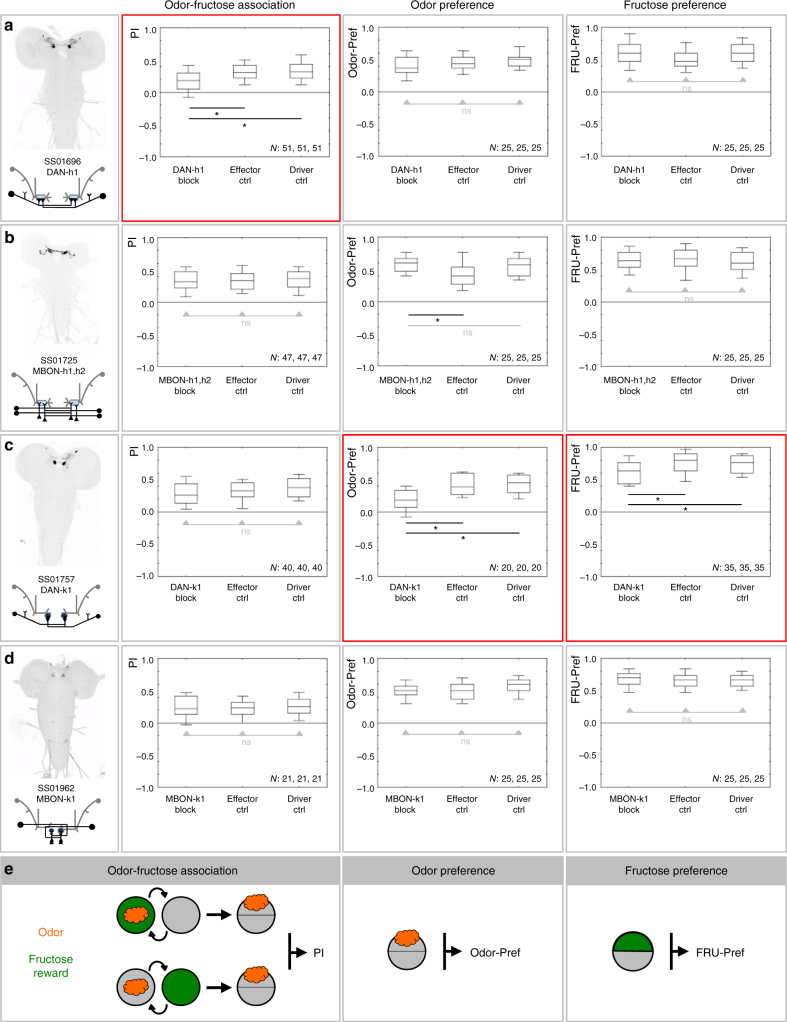
Requirement of shaft and lower-toe MBE neurons for odor-fructose reward association, odor preference, and fructose preference. **a**–**d** Expression pattern of the indicated split-Gal4 strain and schematic overview of the covered MBE (leftmost column), associative performance indices for odor-fructose reward associative memory (second column), and preference scores of experimentally naïve larvae for the odor (third column) as well as for fructose (rightmost column). Experimental larvae are heterozygous for the indicated split-Gal4 drivers and for *UAS*-*Kir2.1::GFP*, leading to silencing of the respective MBE (MBE block). Control larvae are heterozygous for only the *UAS*-*Kir2.1::GFP* effector (effector Ctrl), or for only the split-Gal4 drivers (driver Ctrl). Box plots show the median as the middle line, and 25/75% and 10/90% quantiles as box boundaries and whiskers, respectively. Sample sizes are indicated within the figure. At plain horizontal lines * refers to *P* < 0.05/2 and ns to *P* > 0.05/2 in Mann–Whitney *U*-tests; at horizontal lines with arrowheads, ns refers to *P* > 0.05 in Kruskal–Wallis tests. Significant phenotypes upon silencing a given MBE are marked by red frames. The preference scores underlying the associative performance indices in the leftmost panels can be found in Supplementary Fig. 6. Expression patterns of split-Gal4 drivers covering the respective MBE are visible based on anti-GFP staining (black); the central nervous system is visible based on background fluorescence (gray). **e** From left to right the panel shows schematics of the odor-fructose reward association task, and of the odor preference or fructose preference tasks. The orange cloud indicates *n*-amylacetate as the odor, the green circle indicates the fructose reward. In half of the cases the sequence of training trials was as indicated in the leftmost display, while for the other half it was reversed (not shown)

**Fig. 4:**
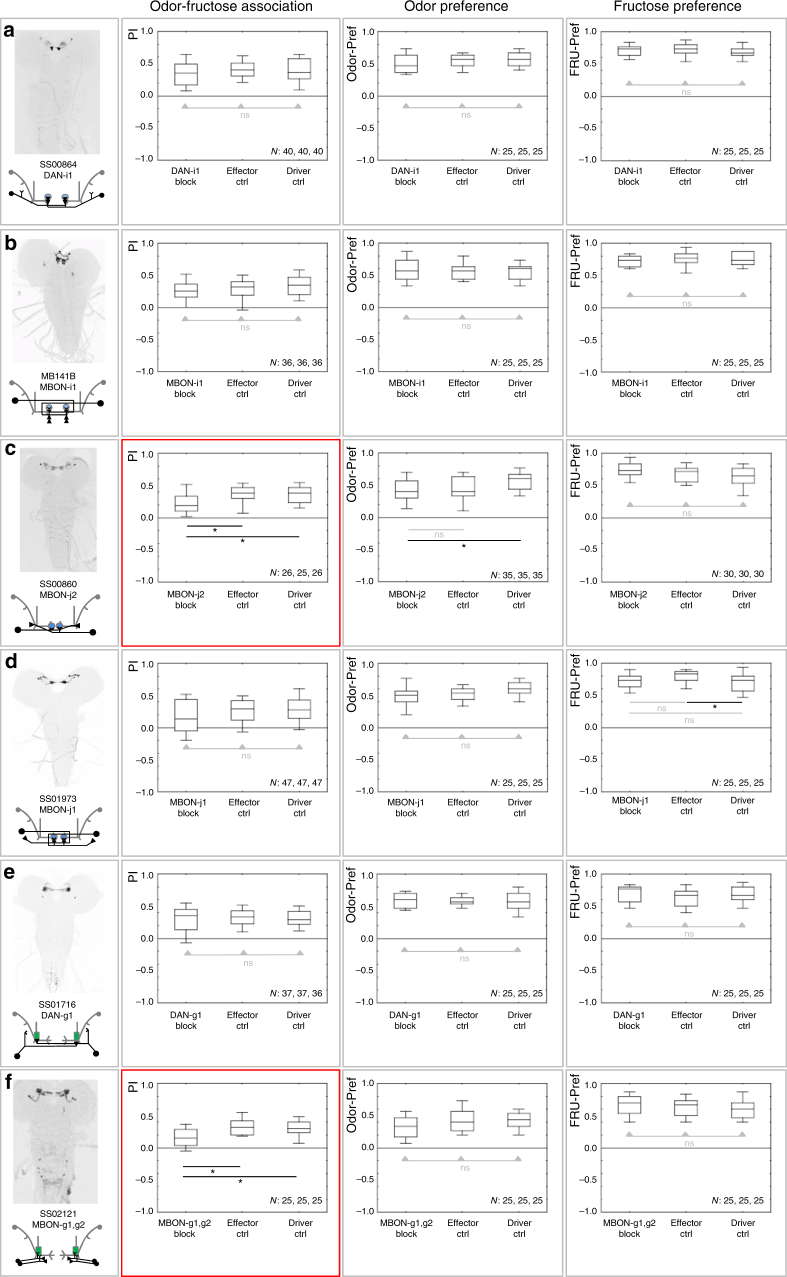
Requirement of upper and middle toe and of lower vertical lobe MBE neurons across behavioral tasks. **a**–**f** Analyses corresponding to the ones from Fig. 3a–d, for the upper and middle toe and of lower vertical lobe MBE neurons. Columns show, from left to right, the expression pattern of the indicated split-Gal4 strain and schematic overview of the covered MBE, the associative performance indices for odor-fructose reward associative memory, and the preference scores of experimentally naïve larvae for the odor as well as for fructose. Experimental larvae are heterozygous for the indicated split-Gal4 drivers and for *UAS*-*Kir2.1::GFP*, leading to silencing of the respective MBE (MBE block). Control larvae are heterozygous for only the *UAS*-*Kir2.1::GFP* effector (effector Ctrl), or for only the split-Gal4 drivers (driver Ctrl). Sample sizes are indicated within the figure. At plain horizontal lines * refers to *P* < 0.05/2 and ns to *P* > 0.05/2 in Mann–Whitney *U*-tests; at horizontal lines with arrowheads, ns refers to *P* > 0.05 in Kruskal–Wallis tests. Significant phenotypes upon silencing a given MBE are marked by red frames. The preference scores underlying the associative performance indices in the leftmost panels can be found in Supplementary Fig. 6. Expression patterns of split-Gal4 drivers covering the respective MBE are visible based on anti-GFP staining (black); the central nervous system is visible based on background fluorescence (gray). Behavioral tasks and other details were as in Fig. 3e

Next, a collection of 12 split-Gal4 driver strains was generated expressing reliably, strongly, and specifically in 13 MBEs (Supplementary Table 4; in three cases, the drivers were specific for the indicated double pair). These driver strains were crossed to *UAS*-*Kir2.1::GFP* to silence neuronal activity by an immunohistologically detectable transgene; expression of *Kir2.1::GFP* was confirmed in all cases (not shown). The larvae were then assayed for (i) odor-FRU reward association, as well as for (ii) odor preference and for (iii) FRU preference (Figs. 3 and 4). Our results show that MBEs play a sparse and specific role in these tasks (Figs. 1e, 3, and 4). That is, in three cases impairments specifically concerned the odor-FRU association task, but not odor preference and not FRU preference (DAN-h1: Fig. 3a; MBON-j2: Fig. 4c; MBON-g1,g2: Fig. 4f; Supplementary Fig. 6a, g, and j). In one case an impairment was found in the preference for odor as well as for FRU, but no impairment of the odor-FRU association (DAN-k1: Fig. 3c; Supplementary Fig. 6c, Supplementary Fig. 7a and b). Silencing DAN-g1 impaired none of these three tasks (Fig. 4e), but rendered olfactory behavior more susceptible to non-associative effects of odor-only as well as of FRU-only exposure (Supplementary Fig. 8). In the remaining five cases, no phenotype in any of the tasks was observed (MBON-h1,h2: Fig. 3b; MBON-k1: Fig. 3d; DAN-i1: Fig. 4a; MBON-i1: Fig. 4b; MBON-j1: Fig. 4d; Supplementary Fig. 6b, d, e, f, and h). The fact that all observed impairments were partial suggests a fair degree of redundancy for the studied MBE neurons and behavioral tasks. Strikingly, no case was observed of impairment in all tasks. Together, these results suggest sparseness and specificity in the requirement of the studied MBE neurons for odor-FRU association, odor preference, and FRU preference (Fig. 1e).

### Sufficiency of DAN-h1 and DAN-i1 as internal reward signal

**Fig. 5:**
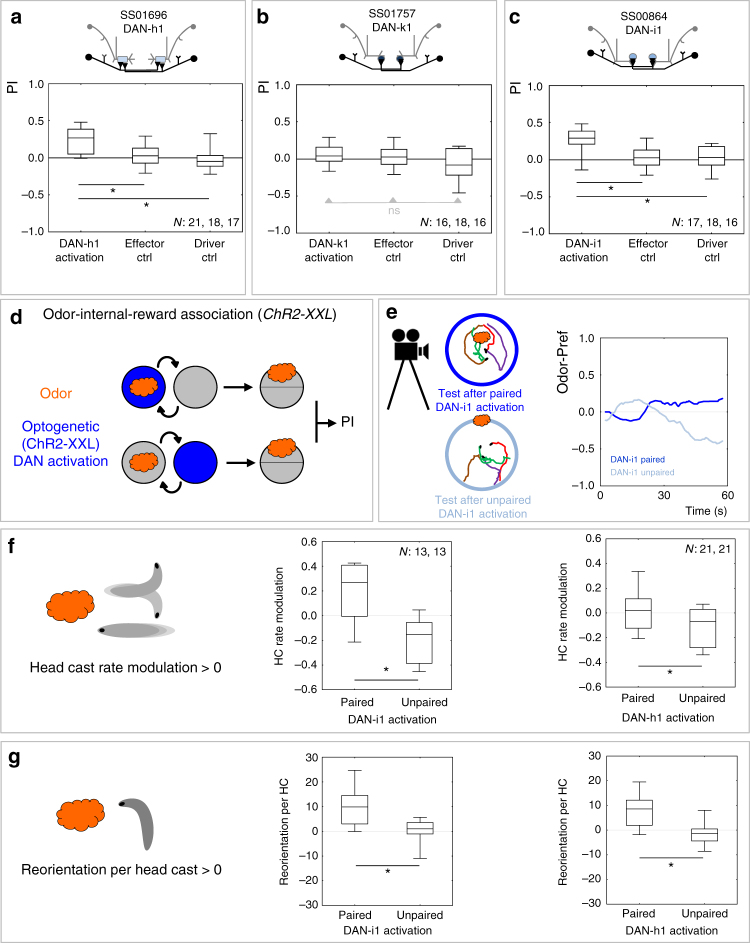
Sufficiency of DAN-h1 and of DAN-i1 as an internal reward signal. **a**–**c** Larvae are trained for association of odor with optogenetic activation of dopaminergic mushroom body input neurons (DANs) as an internal reward. The panels show a schematic of the covered DAN, plus the associative performance indices after odor-internal-reward training. Experimental larvae are heterozygous for the split-Gal4 drivers as well as *UAS-ChR2-XXL* (DAN activation). Control larvae are heterozygous for only the *UAS-ChR2-XXL* effector (effector Ctrl), or only the indicated split-Gal4 drivers (driver Ctrl). Activation of DAN-h1 (**a**) and activation of DAN-i1 (**c**) is sufficient as a reward, whereas activation of DAN-k1 is not (**b**). Other details as in Fig. 3. The preference scores underlying the associative performance indices can be found in Supplementary Fig. 9. **d** Schematic of the odor-internal-reward association task. The blue color indicates optogenetic activation of the respectively covered neuron. Other details as in Fig. 3. **e**–**g** Locomotor “footprint” of memories established by optogenetic DAN activation as reward. **e** Sample tracks by individual larvae recorded after training with paired (top left) or unpaired (bottom left) presentation of odor and DAN-i1 activation. Right: after paired training with odor and DAN-i1 activation, larvae have a higher preference for the odor than after unpaired training. Displayed is the median preference of the full sample (*N* = 13 with approx. 30 animals each) over time (sliding average 5 s). Similar results were observed for DAN-h1 activation (not shown). **f** Modulations of head cast (HC) rate. After paired training with either DAN-i1 (middle panel) or DAN-h1 activation (right panel) the larvae perform more HCs while they are heading away from the odor than while they are heading toward it, measured as a positive HC rate modulation; after unpaired training the opposite effect is observed. **g** HC direction. Larvae direct their HCs more toward the odor after paired training than after unpaired training with DAN-i1 (middle panel) or DAN-h1 activation (right panel). Sample sizes are indicated within the figure. *P < 0.05 in Mann–Whitney *U*-tests. Other details as in Fig. 3

The MBIN found to be required for full performance in the odor-FRU association task was DAN-h1, a member of the pPAM cluster of DANs innervating the medial lobe^42^ (Fig. 3a). This raised the question whether this and/or other pPAM neurons are also sufficient for mediating an internal reward signal. To this end, instead of presenting a real FRU reward, ChR2-XXL was used to optogenetically activate DAN-h1, DAN-k1, or DAN-i1 as an internal reward signal (attempts to generate a suitable driver strain for DAN-j1 have failed). Activation of DAN-h1 as well as activation of DAN-i1 was sufficient as an internal reward signal, but activation of DAN-k1 was without any such effect (Fig. 5a–d; Supplementary Fig. 9a-d). While the rewarding effect of activating either DAN-h1 or DAN-i1 was replicated with a tomato-tagged version of ChR2-XXL (Supplementary Fig. 9e), using Chrimson as an optogenetic effector yielded a rewarding effect only for DAN-i1 (Supplementary Fig. 10). Thus, levels of activation by Chrimson may be less for DAN-h1 than for DAN-i1. Alternatively, Chrimson may be a generally weaker activator than ChR2-XXL such that a stronger rewarding effect of DAN-i1 than of DAN-h1 is revealed with Chrimson but, because of a ceiling effect, not with ChR2-XXL.

### Behavioral footprint of DAN-h1 and DAN-i1 induced memories

The behavioral properties of memories established by DAN-i1 or DAN-h1 reinforcement were explored by means of video tracking (Fig. 5e–g). Optogenetically induced associative memory turned out to have the same “footprint” as memory established by FRU as a real reward^53^. That is, the innate bias of the larvae to run straight rather than turn when heading toward an odor source was strengthened after paired presentations of odor and DAN-i1 activation; likewise their tendency to direct turns toward, rather than away, from the odor source was strengthened (Fig. 5f, g; after presentations of odor unpaired from DAN-i1 activation both these effects were reverse). Corresponding effects, albeit somewhat weaker, were observed for DAN-h1 activation (Fig. 5f, g).

### Timing-dependent valence reversal of DAN-i1 reinforcement

**Fig. 6:**
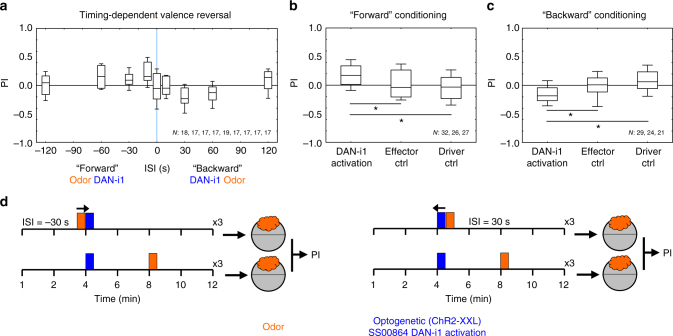
Timing-dependent valence reversal of optogenetic DAN-i1 reinforcement. **a** Larvae of the experimental genotype (heterozygous for SS00864-Gal4 and *UAS-ChR2-XXL*) were trained by presenting an odor and optogenetically activating DAN-i1, at various relative timings. A 30-s odor presentation either preceded a 30-s DAN-i1 activation (forward conditioning, plotted as negative inter-stimulus interval, ISI) or the odor followed DAN-i1 activation (backward conditioning, positive ISI). Subsequently, larvae were tested for their odor preference. For short-interval forward conditioning, positive scores reveal appetitive memory. For short-interval backward conditioning, negative scores indicate aversive memory. **b** Appetitive memory scores were confirmed after forward conditioning (ISI of −10 s) for the experimental genotype (heterozygous for SS00864-Gal4 and *UAS-ChR2-XXL* (DAN-i1 activation)). Control larvae heterozygous for only *UAS-ChR2-XXL* (effector Ctrl) or SS00864-Gal4 (driver Ctrl) did not show any memory. **c** Aversive memory scores were also confirmed after backward conditioning (ISI of 30 s) for the experimental genotype (DAN-i1 activation). Again, genetic controls did not show any memory. The preference scores underlying the associative performance indices can be found in Supplementary Fig. 11. Sample sizes are indicated within the figure. Other details as in Fig. 3. **d** Schematic of the timed odor-DAN-i1 association protocol. Orange and blue colors indicate the timing of odor presentation and optogenetic activation of DAN-i1, respectively, during each of the three 12-min training trials. In the example timeline, paired training (top) involves intervals between the onset of odor presentation and optogenetic activation of −30 s (left) and 30 s (right); the timing of events for unpaired training is shown to the bottom. After either paired or unpaired training, larvae are tested for their preference for the odor; the associative performance indices (PIs) are then calculated from the difference in preference between paired-trained and unpaired-trained larvae

**Fig. 7:**
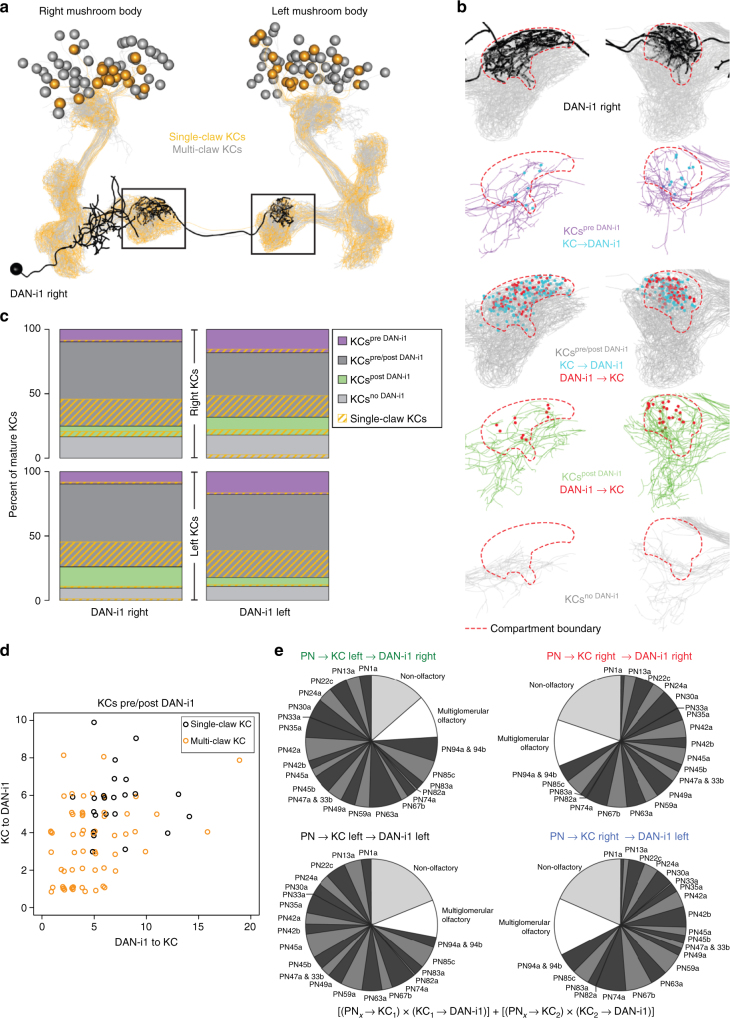
Structure of DAN-i1 and connectivity with KCs. **a**–**d** KC-to-DAN-i1 and DAN-i1-to-KC connections. **a**, **b** Electron microscopy reconstruction of the right-hemisphere DAN-i1 neuron and the mushroom body Kenyon cells (KCs). The regions highlighted by the box are shown in **b** for all KCs, and separated for KCs only presynaptic, pre- and postsynaptic, only postsynaptic, or not connected to DAN-i1, at a slightly tilted view relative to **a**. KC-to-DAN-i1 and DAN-i1-to-KC synapses are marked in blue and red, respectively. Inter-hemispheric connections are from aberrantly developed KCs, not from the so-called ni-cells^62^. The left-hemisphere DAN-i1 neuron is organized correspondingly (not shown). **c** Quantification of the numbers of single- and multiple-claw KCs synaptically connected to DAN-i1 (or not), separated for KCs that are only presynaptic, pre- and postsynaptic, only postsynaptic, or not connected to DAN-i1. This analysis does not suggest discrepancies between the right- and left-hemisphere DAN-i1, or between the DAN-i1 innervation of ipsi- and contralateral KCs. **d** For each KC reciprocally connected with DAN-i1, the numbers of KC-to-DAN-i1 versus DAN-i1-to-KC synapses are plotted (Supplementary Fig. 13a shows the data separated by hemisphere; Supplementary Fig. 13b plots each KC’s connections to the ipsi- and contralateral DAN-i1). The observed correlation (Spearman rank correlation analyses at *P* < 0.05) contrasts with the case of the APL neuron (Fig. 9c). **e** Relation of DAN-i1 to the KC inputs. The total input that DAN-i1 receives from KCs that in turn receive their input from the indicated classes on PNs was considered to calculate the matrix product of the respective PN-to-KC and KC-to-DAN-i1 connections. This was done separately for the right-hemisphere DAN-i1 and its connections to the contralateral and the ipsilateral KCs (top), and for the left-hemisphere DAN-i1 (bottom). The observed correlations (also see Supplementary Fig. 13c) reveal that both DAN-i1s sample the KC coding space in the same way (corresponding to the case of the APL neuron: Fig. 9d and Supplementary Fig. 17a), and that both DAN-i1s sample the ipsi- and contralateral KC coding space in the same way. A visualization of DAN-i1 connectivity within the mushroom body using a standard force-directed algorithm^103^ can be found in Supplementary Movie 6

We further asked whether the valence of the reinforcing effect of DAN-i1 activation is timing-dependent (Fig. 6, Supplementary Fig. 11; for a detailed account of DAN-i1 structure and connectivity: Fig. 7, Supplementary Fig. 12, Supplementary Fig. 13). That is, in mammals and bees, presenting a cue before presenting a real reward can establish an association of the cue with reward gain, whereas presenting the cue only upon termination of a reward can establish an association with reward loss^63, 64^. We found that “forward” pairings of odor followed by DAN-i1 activation induced appetitive memory, whereas “backward” pairings of DAN-i1 activation followed by odor induced aversive memory (Fig. 6). Thus, larval *Drosophila* show timing-dependent valence reversal. Specifically, DAN-i1 activation can be sufficient for appetitive learning about receiving a reward, and for aversive learning about reward termination.

### DANs differ in the kinds of reward they mediate

**Fig. 8:**
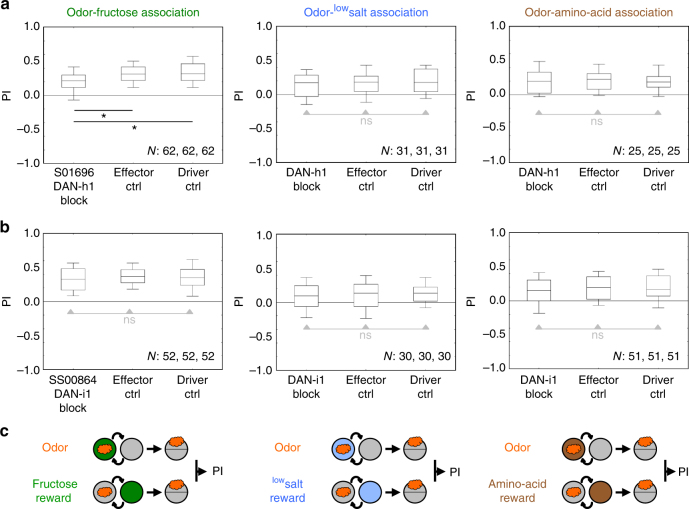
Requirement of DAN-h1, but not DAN-i1, for specifically odor-fructose association. **a** Silencing of DAN-h1 by means of *Kir2.1::GFP* expression leads to an impairment in odor-fructose association (left) (these data include the odor-fructose association data shown in Fig. 3a), but not in odor-^low^salt (middle) and not in odor-amino-acid association (right). **b** Silencing DAN-i1 does not impair association of odor with any of these three rewards (odor-fructose association data include those shown in Fig. 4a). Likewise, odor-arabinose association as well as odor-sorbitol association is unaffected by silencing of DAN-i1 (Supplementary Fig. 15). The preference scores underlying the associative performance indices can be found in Supplementary Fig. 14. **c** From left to right, schematics of the indicated association tasks are shown. The green, light blue, and brown circles indicate the fructose, odor-^low^salt, and amino-acid reward, respectively. Other details as in Fig. 3

It is striking that activation of either DAN-h1 or DAN-i1 is sufficient to mediate a “virtual” internal reward signal (Fig. 5, Supplementary Fig. 9e), whereas only DAN-h1 activity participates in mediating the rewarding effect of a real FRU reward (Figs. 3a and 4a). Is it that DAN-i1 is required for mediating other rewards, whereas for those other rewards DAN-h1 is dispensable? Silencing DAN-h1 impaired learning about a FRU reward, but not learning about low-salt or aspartic acid (ASP) rewards (Fig. 8a, Supplementary Fig. 14). By contrast, silencing DAN-i1 did not impair learning about any of the known taste rewards (FRU, low salt, and ASP: Fig. 8b, Supplementary Fig. 14; arabinose (ARA) and sorbitol (SOR): Supplementary Fig. 15). Whether silencing DAN-i1 would affect learning about any hitherto uncharacterized reward X remains to be tested. In any event, these results show that more than one DAN can mediate an internal reward signal, and that DANs differ with respect to the kinds of reward they mediate. Does a corresponding separation of processing channels carry through to mushroom body output?

### Requirement of MBINs and MBONs does not match by compartment

In none of the studied cases did the requirement of MBINs and MBONs for a task match by compartment (Figs. 1e, 3, and 4). That is, the only MBIN found to be required for proper performance in the odor-FRU association task innervates the medial lobe shaft (DAN-h1: Fig. 3a), but silencing the MBONs from this compartment does not impair performance (MBON-h1,h2: Fig. 3b, Supplementary Fig. 16). Rather, silencing MBONs from two other compartments causes impairments in odor-FRU association scores (MBON-j2 from the intermediate toe: Fig. 4c; MBON-g1,g2 from the lower vertical lobe: Fig. 4f).

Likewise, silencing the MBIN to the lower toe impairs both odor and FRU preference (DAN-k1: Fig. 3c, Supplementary Fig. 7a and b), whereas silencing its output neuron does not (MBON-k1: Fig. 3d, Supplementary Fig. 7c and d).

Last but not least, silencing the MBIN to the lower vertical lobe modulates the effects of non-associative exposure to either odor or FRU (DAN-g1: Supplementary Fig. 8) but neither odor-FRU association nor odor preference or FRU preference (Fig. 4e). By contrast, the MBONs from this compartment affect odor-FRU association (MBON-g1,g2: Fig. 4f).

This suggests that within-mushroom-body and between-compartment cross talk takes place during the organization of behavior. Does the larval mushroom body provide a circuit-level substrate for such cross talk?

### Circuit motifs for integration across compartments

**Fig. 9:**
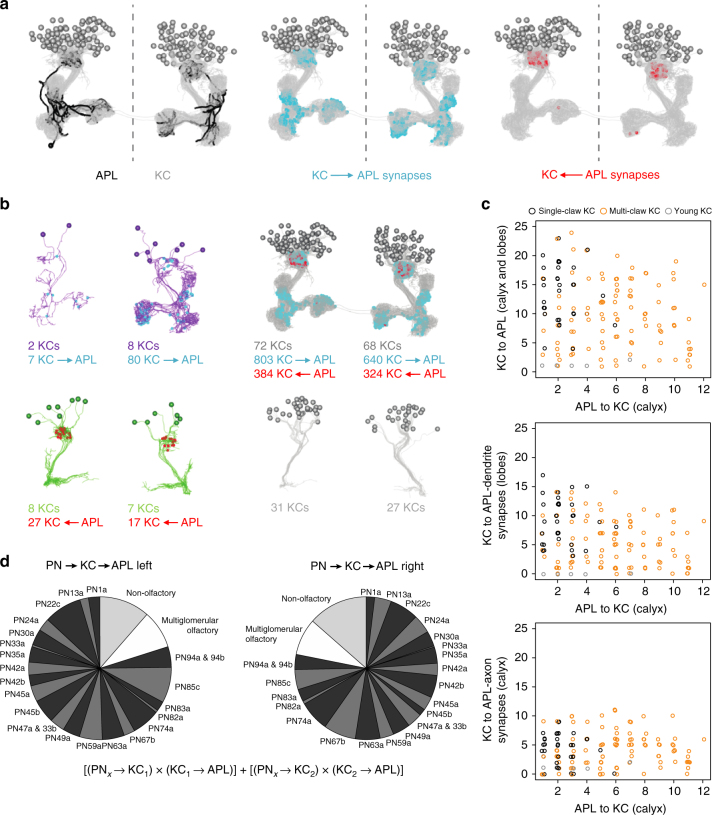
Structure and connectivity of the APL neuron. The larval APL neuron provides distributive inhibition within the mushroom body. **a** Electron microscopy reconstruction of the APL neuron relative to the mushroom body Kenyon cells (KCs) in both brain hemispheres (left panel). Sites of KC-to-APL synapses are marked in blue (middle panel) and sites of APL-to-KC synapses in red (right panel). Inter-hemispheric connections are from aberrantly developed “freak” KCs and do not correspond to the so-called ni-cells identified by Kunz et al.^62^; these were found in the electron microscopy reconstruction but are not shown here. **b** KCs are classified as either being only presynaptic, as both pre- and postsynaptic, as only postsynaptic, or as unconnected to the APL neuron. The anatomy of these KC classes is shown, including their number and the number and the site of KC-to-APL synapses (blue) and APL-to-KC synapses (red). KCs without any synapse with the APL neuron are apparently lacking proper dendrites and axons, and therefore are classified as young and probably immature. **c** For each KC the number of APL-to-KC synapses versus respectively the number of all KC-to-APL synapses (top), the number of KC to APL-dendrite synapses (middle), and the number of KC to APL-axon synapses are plotted (bottom). The type of KC (single-claw, multi-claw, and young KC) is color-coded. The lack of correlation implies that inhibition through the APL neuron is distributive, i.e., with only random levels of KC_x_-APL-KC_x_ feedback. This lack of correlation contrasts to the case of the reciprocal connections between the KCs and the DAN-i1 neuron (Fig. 7d). A quantification of the connections of APL with non-KCs can be found in Supplementary Fig. 17b. **d** The APL neuron samples the complete information delivered to the KCs via projection neurons (PNs). To determine the total input that the APL neuron receives from KCs that in turn receive their input from uniglomerular olfactory PNs (black and dark gray), multiglomerular olfactory PNs (light gray), and non-olfactory PNs (white), the matrix product of the respective PN-to-KC connections and the KC-to-APL connections was calculated. The sampling of the KC coding space by the right- and the left-hemisphere APL neuron is strongly correlated (Supplementary Fig. 17a). A visualization of APL connectivity within the mushroom body using a standard force-directed algorithm^103^ and based on the connectivity data available with ref. ^7^ can be found in Supplementary Movie 7

At least four circuit motifs could provide intercompartmental cross talk, possibly in addition to molecular mechanisms of cross-compartmental integration within the KCs. First, four mMBONs integrate inputs from multiple compartments (MBON-e2, MBON-m1, MBON-n1, and MBON-d3: Fig. 2), and of these MBON-d3 also integrates across hemispheres. Second, KC-KC synapses provide direct lateral connections^7^. Third, MBON-MBON synapses establish an intricate network of mushroom body efferent circuitry^7^. Fourth, the GABAergic APL neuron integrates input from nearly half of the compartments and provides inhibition toward the calyx, where the signal is distributed across the KCs (Fig. 9a, b, Supplementary Fig. 17). Indeed, a connectivity analysis of the APL neuron revealed that very few KCs are either exclusively presynaptic or exclusively postsynaptic to it, and that for a given KC_x_ the number of KC_x_-APL synapses is unrelated to the number of APL-KC_x_ synapses; rather, APL establishes distributive inhibition such that most KCs receive inhibition proportional to an integrated read-out of the activity in all KCs and across at least six compartments^7^ (Figs. 2 and 9c). In turn, the APL neuron samples most, if not all, of the olfactory input that the olfactory projection neurons deliver toward the KCs, plus a sizeable non-olfactory input^7^ (Fig. 9d, Supplementary Fig. 17). Taken together, there is an ample substrate for circuit-level integration across the mushroom body and across compartments. The behavioral significance of such integration was explored, taking the APL neuron as an example.

### Silencing or driving APL impairs odor-FRU association

**Fig. 10:**
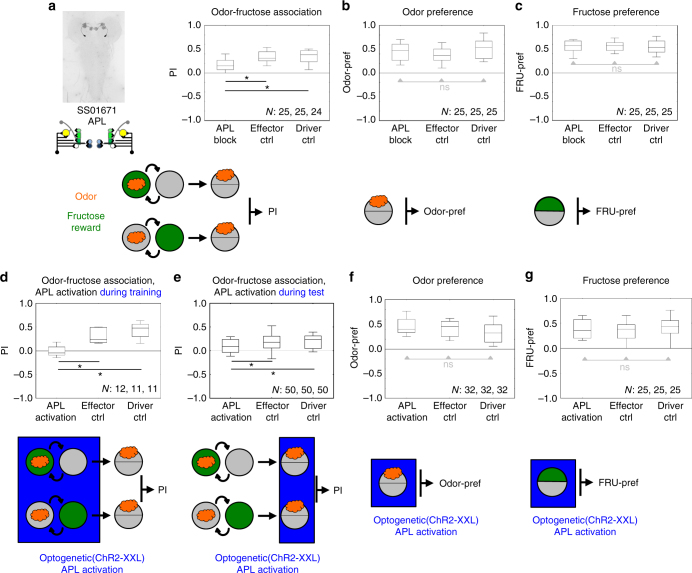
Behavioral function of the APL neuron. **a** Silencing the APL neuron impairs odor-fructose association. Shown are the expression pattern of the split-Gal4 strain covering the APL neuron and a schematic of its innervation of the mushroom body (top left), associative performance indices for odor-fructose reward associative memory (top right), and a schematic of the behavioral task. Experimental larvae are heterozygous for the split-Gal4 driver and for *UAS*-*Kir2.1::GFP* (APL block). Control larvae are heterozygous for only the *UAS*-*Kir2.1::GFP* effector (effector Ctrl), or for only the split-Gal4 driver (driver Ctrl). Other details as in Fig. 3. The preference scores underlying the associative performance indices can be found in Supplementary Fig. 6. **b** Silencing the APL neuron does not impair innate odor preference; other details as in **a**. **c** Silencing the APL neuron does not impair innate fructose preference; other details as in **a**. **d** Optogenetic activation of the APL neuron during training abolishes odor-fructose association. Experimental larvae are heterozygous for the split-Gal4 driver as well as *UAS-ChR2-XXL* (APL activation). Control larvae are heterozygous for only the *UAS-ChR2-XXL* effector (effector Ctrl), or for only the split-Gal4 drivers (driver Ctrl). Other details as in Figs. 3 and 5a–d. The preference scores underlying the associative performance indices can be found in Supplementary Fig. 18. The bottom of the panel shows a schematic of the behavioral task. Blue shading indicates optogenetic stimulation. Other details as in Fig. 3. **e** As in **d**, showing that activation of the APL neuron during only the test reduces odor-fructose association scores. **f**, **g** As in **b**, **c**, showing that activation of the APL neuron in experimentally naïve animals affects neither odor preference nor fructose preference

Cross-compartmental integration via the GABAergic APL neuron is required for proper odor-FRU association, as shown by the impairment in this task upon blocking APL; this requirement is specific, as both odor preference and FRU preference remain unchanged (Fig. 10a–c, Supplementary Fig. 6l). Thus, without the possibility to maintain a sparse activity pattern across the KCs the larvae are unable to perform properly in the present seemingly non-discriminatory association task. Arguably, this is because such sparseness is required to discriminate olfactory stimulus foreground from non-olfactory, contextual background (see Discussion). In turn, optogenetically driving the GABAergic APL neuron should effectively silence the KCs; fittingly, under such conditions odor-FRU association is impaired, both when such GABAergic activation is exerted during the training phase only, and when it is exerted only during testing (Fig. 10d, e, Supplementary Fig. 18). Again, this effect is specific, as driving the APL neuron affects neither odor preference nor FRU preference (Fig. 10f, g).

## Discussion

The present study reveals the identity and morphology of almost all neurons of the MBIN-MBON-APL network of stage 3 *Drosophila* larvae. For a subset of these neurons, it provides a collection of split-Gal4 drivers for specific manipulation and a survey of their function in olfactory preference, gustatory preference, and odor-taste associative learning.

A stage 3 larva shares characters with its earlier self as a stage 1 larva and its later self as an adult fly^65, 66^. Across the larval stages body length and speed of locomotion increase about fourfold. Nevertheless, the behavioral faculties of stage 1 larvae qualitatively match those of stage 3^66^. Appropriately, the number and morphology of their MBINs and MBONs and the morphology of the APL neuron are similar^7^ (Supplementary Fig. 1, Supplementary Fig. 2)—with the absence of the DAN-h1 neuron in stage 1 as the arguably most obvious exception. Despite this absence of DAN-h1, however, stage 1 larvae are capable of odor-FRU association^17, 61^. This is consistent with our observation in stage 3 larvae that silencing DAN-h1 only partially impairs odor-FRU association (Figs. 3a and 8a).

The organization of the MBIN-MBON-APL network in stage 3 larvae is also similar to that in adult *Drosophila*^56^, with at least three notable exceptions. First, the number of PAM-cluster DANs increases from 1 to up to 20 (sic) per cell type and compartment in adult *Drosophila*^56^. PAM-DANs preferentially innervate the medial lobes and are responsible for reward learning in both larval and adult *Drosophila*^39, 40, 42^. An increase in the number of PAM-DANs thus suggests a better signal-to-noise ratio for reward processing in adult *Drosophila*. Differences in the sensitivity to reward between PAM-DANs would broaden the dynamic range across which reward strength can be mapped in adult *Drosophila*. Higher-sensitivity and broader-range mapping of memory strength to reward strength is relevant when memory formation or memory-based decisions need to be quick, for example, when facing shallow gradients during fast walking or during flight. Qualitative differences in the reward inputs that different PAM-DANs receive would additionally allow for more nuanced processing of reward kind in adult *Drosophila*. Together, this may be critical for adult flies to keep track of rewards that are further away, more variable in strength, time, or space, or that are of relevance for adults only, such as sexual rewards. By contrast, the representation of punishment, for the most part mediated by PPL-DANs^36, 37^ (adult *Drosophila*^35, 38, 52^), does not differ in numbers of neurons between larvae and adults.

Second, neither our inspection of the driver lines of the Janelia collection nor our extensive flp-out analysis nor the electron microscope reconstruction in a stage 1 larva^7^ revealed a larval dorsal paired median (DPM) neuron^67^. Further, none of the driver strains known to cover the DPM neuron in adult *Drosophila* includes a larval DPM neuron (Thum, unpublished). In adult *Drosophila*, the DPM neuron innervates the ipsilateral mushroom body and ramifies throughout it, excluding the calyx. The DPM neuron produces the amnesiac neuropeptide^67^, serotonin^68^, and GABA^69^. In accordance with the absence of the DPM neuron in the larva, there is no detectable immunoreactivity against serotonin in its mushroom body^70^.

Third, in the larva KC-APL synapses are present in the mushroom body calyx and lobes, but APL-KC synapses are restricted to the calyx (Fig. 9a, b). In addition to a local KC-APL-KC network in the calyx, the APL neuron may thus establish a lobe-to-calyx connection in larvae, reminiscent of mushroom body feedback neurons in the bee^71^. No such polarity is apparent in adult *Drosophila* (i.e., KC-APL and APL-KC synapses are intermingled in both calyx and lobes^56, 72, 73^). Furthermore, in the case of the larva the APL neuron innervates 7 out of 11 mushroom body compartments (Figs. 2 and 9a, b), whereas the APL neuron innervates all MB compartments in adult *Drosophila*^72, 73^. Differences in function between the larval and the adult APL neuron are also to be expected because the DPM neuron, anatomically and functionally entangled with APL in adult *Drosophila*^73^, is absent in the larva (see previous paragraph). Of note is that despite these differences in structure and probably also in function, the larval APL neuron does persist through metamorphosis (Supplementary Fig. 19).

Larvae form associative memories between odor and taste reward that can be specific not only for the odor^74, 75^ but also for the reward^55^. That is, after odor-FRU reward training learned odor attraction is abolished if the test is carried out in the presence of FRU^55, 76, 77^ (innate olfactory behavior is not likewise affected). This is adaptive because tracking down the learned odor in search of a reward is pointless if the sought-for reward is actually present. Indeed, learned search after odor-FRU training is abolished in the presence of FRU, but not in the presence of an equally strong ASP reward, and vice versa^55^. Thus, memories from odor-FRU and from odor-ASP training can be established and behaviorally expressed independently of each other. Accordingly, we found that activity in DAN-h1 is required for proper odor-FRU association but is dispensable for odor-ASP association (Fig. 8a). This suggests a division of labor between DANs for signaling FRU (and similar rewards) and other DANs for signaling ASP (and similar rewards; see the following section). Indeed, a similar picture may be emerging for adult *Drosophila* too^39, 78^. Once it becomes possible to study defined subsets of DANs in mammals, it will be feasible to test for similar breaches of the common-currency hypothesis of DAN function^79^. It is noteworthy that behavioral analyses suggest that reward-general and reward-specific memories co-exist in the larva^55^ (regarding humans^80^).

Our results suggest that the requirement for MBE activity in behavior organization is sparse and specific. For each behavioral tasks under study, it was only in a few MBEs that silencing activity had a measurable effect; conversely, for each MBE under study, the requirement for their activity was task-specific (Fig. 1e). For example, silencing DAN-h1 left odor preference and FRU preference intact (Fig. 3a), yet selectively impaired the odor-FRU association, but neither the odor-ASP association nor the association of odor with low-concentration salt as a reward (Figs. 3a and 8a). This suggests that MBIN identity may carry at least some non-redundant information about reward kind. On the other hand, in this as in all other cases of impairment, the impairment was partial, suggesting an at least partially redundant code. Together, the way MBINs process rewards is discrete and sparse enough for single MBINs to contribute measurably to the processing of specific rewards, yet broad enough such that ensuing impairments are only partial.

For the studied MBONs as well, the impairments upon silencing them were sparse, selective, and partial (Fig. 1e). Strikingly, the impairments in odor-FRU association did not match the impairments observed upon silencing the MBINs to the same compartment. Thus, although the physiological effects of MBIN-KC coincidence may remain local to the MBINs’ target compartment (as shown in adult *Drosophila*^52^), the organization of learned behavior apparently involves cross-compartmental integration. This is consistent with a view of the MBON network as providing a learned-valence signal integrated across large aspects if not the whole of the mushroom body, and thus reflecting most if not all memories associated with a situation. Summing this learned-valence signal with the innate valence of the stimuli comprising that situation will result in approach or avoidance^53^ (for adult *Drosophila*^49^). Within the mushroom body, relatively high-dimensional representations of predictive stimuli (across the KCs) and of reinforcement (across the MBINs) are thus “reformatted” to provide a rather low-dimensional output of behavioral valence.

Optogenetic activation of DAN-i1 is sufficient as an internal reward signal (Fig. 5a, e–g, Supplementary Fig. 9e, Supplementary Fig. 10e). Specifically, it is sufficient for two types of reward learning: appetitive learning about receiving a reward during odor-then-DAN-i1 training, and aversive learning about reward termination upon DAN-i1-then-odor training (Fig. 6). This conforms to earlier suggestions that it is the temporal difference in reinforcement that determines affect and memory valence^63, 64, 81–83^: it feels good to receive a reward but its termination feels bad, resulting respectively in appetitive and aversive learning of the associated cues. This implies that the onset of DAN-i1 activity is rewarding, while the offset of DAN-i1 activity is punishing. Conversely, receiving punishment versus being relieved from it respectively supports aversive or appetitive learning^64, 84–86^. Such timing-dependency is also a feature of molecular coincidence detection^87^, and of synaptic plasticity^88^. This has inspired computational models relating timing-dependent valence reversal at the behavioral level to the timing-dependent reversal in the “sign” of synaptic plasticity^83, 89^.

The APL neuron collects input from and distributes inhibitory GABAergic output across the KCs^22^ (Fig. 9a–c; adult *Drosophila*^56, 72, 73^). The level of activity in a given KC will thus be scaled to the level of activity across the KCs. Innate preference for odor and for FRU are not affected by altering APL neuron function, while odor-FRU association is impaired both by silencing and by driving the APL neuron (Fig. 10). Why is this so?

When encountering an odor, a subset of KCs will be phasically activated. Further KCs may be activated by tonic contextual input. Together these inputs can drive the APL neuron^22^ (adult *Drosophila*^72, 90, 91^ and bees^92^). Distributive GABAergic inhibition through APL will reduce activity in strongly activated KCs, and silence weakly active KCs. In effect, the strongest, most salient input will dominate the KC activity pattern (winner takes all). When at the same time a reward such as FRU is presented, it will be this most salient input that preferentially enters into association with it. Thus, in our paradigm the salient, phasic olfactory “foreground” rather than the tonic, non-olfactory contextual “background” will be associated with FRU. Strongly reducing APL activity will lift inhibition of the KCs and disable the winner-take-all mechanism, such that the odor will be less discriminable from the background. Mildly increasing APL activity will make the olfactory input more distinct from the background. Strongly driving APL, however, may silence the KCs altogether. Such a scenario can explain why both strongly reducing and strongly driving APL reduces performance in the one-odor, non-discriminatory paradigm of the present study (Fig. 10a, d, e).

We note that a modulation of the sparseness of KC activity through APL could tweak the set point between generalization and discrimination of stimuli, which can indeed be adjusted in a task-dependent way in larvae^93^ and adults^94^.

In adult *Drosophila* odor-electric shock training entails an odor-specific and associative weakening of odor-evoked activity in the APL neuron^72^. This corresponds to the synaptic depression that has been observed after associative learning at the KC-MBON synapse in adult *Drosophila*^47, 51, 52^ (bee^4^; but also see refs. ^48, 50^). The proximity of the KC-MBON synapses to the KC-APL synapses^7^ (Supplementary Fig. 20) suggests that local learning-induced molecular alterations at KC-MBON presynaptic sites may affect the closely neighboring KC-APL synapses, too. A strengthening of the memory trace would thus correspond to more severe depression of both the KC-MBON and the KC-APL synapses. In effect, a learned odor would evoke less APL activity and render the KCs more excitable than an untrained odor would.

Together with the upcoming future analyses of the medial lobe MBINs and MBONs and the full synaptic connectome of the MBINs, MBONs, and KCs^7^ the present work provides the most detailed view to date of the structure and behavioral function of the mushroom body in larval *Drosophila*. The apparent principles are largely shared with adult *Drosophila* and other insects^1–6^ providing a study case of how a central brain structure is organized, and how it organizes behavior.

## Methods

### Fly lines

All experiments used third-instar feeding-stage, 5-day-old *Drosophila melanogaster* larvae, raised and treated under standard conditions (25 °C temperature, 65–70% humidity, 12/12 light-dark cycle, and standard food), unless stated otherwise. Larvae were chosen at random from their food vials and used immediately for behavioral experiments. As is usual for larval assays, yet unlike the usual procedure for reward learning in adult *Drosophila*, there was no additional starvation period. Behavioral experiments were performed under adjustable temperature and humidity conditions using a custom-built setup (blueprints are available upon request). For the pre-screen, larvae were incubated in a water bath for 5 min at 36 °C to ensure that synaptic transmission was blocked when using *shibire*^*ts*^ as the effector (see below). Training and test were then performed at a restrictive, yet slightly lower temperature (32 °C).

The statistical analyses used two-tailed, non-parametric tests throughout (Statistica 12.0, StatSoft software, Hamburg, Germany; multiple-group comparison with Kruskal–Wallis tests, in case of significance followed by pairwise comparisons with Mann–Whitney *U*-tests); statistical assumptions for these tests were met. Bonferroni corrections were used to maintain error rates of multiple pairwise comparisons below 5%. Sample sizes were based on previous reports of chemosensory behavior and learning with moderate to weak effect sizes^18, 36, 95^. All behavioral experiments were run in parallel for the respective experimental group and genetic controls. Experimenters were blind to the specific genotypes.

### Odor-FRU reward association for the pre-screen

A standard odor-FRU reward associative learning paradigm was used^95–97^ (Fig. 3e), adapted to facilitate the pre-screen. The pre-screen was performed at HHMI Janelia Research Campus by a team of 4 experimenters of which 1–3 worked in parallel. From a list of Gal4 strains known to cover MBEs from flp-out analyses, sets of approx. six Gal4 strains were randomly selected (without “replacement”) for crossings every week, yielding sample sizes of approximately *N* = 6/genotype and week. Once all genotypes had been tested, this cycle was repeated, yielding sample sizes of typically *N* = 9 or 15/genotype. When information about additional Gal4 strains covering MBEs became available from the flp-out analyses, these strains were included during and/or after the first two cycles of experimentation. Throughout, the genetic control strain was tested in parallel, at approximately *N* = 3/week, yielding *N* = 150 in total.

Groups of 30 larvae each were free to move in 90-mm-diameter plastic Petri dishes filled with either plain 2.5% agar (CAS: 9002-18-0; Fisher BioReagent, Fair Lawn, NJ, USA) or, during rewarded trials, agar with 2 mol/l FRU as a reward (+: CAS: 57-48-7; Fisher Scientific, Pittsburgh, PA, USA). Whenever an odor was to be presented, 10 µl *n*-amylacetate (AM: CAS: 628-63-7, diluted 1:50 in mineral oil: CAS: 8012-95-1; both Avantor Performance Materials, Center Valley, PA, USA) was pipetted onto 5 × 5 mm filter papers taped to the lid of the Petri dish. When no odor was to be presented, a blank filter paper was used.

In the paired group, odor and reward were presented together throughout a given 2.5 min trial (AM+), whereas the unpaired group received odor and reward during separate 2.5 min trials (AM/+). To equate the unpaired and paired group for handling and total training duration, sham trials were alternately performed for the paired group during which neither odor nor reward were presented (AM+/blank). In half of the cases, the sequence of training trials was reversed (blank/AM+ and +/AM, respectively). After three training cycles, preference for AM was assayed by placing the larvae in the middle of a fresh plain-agar Petri dish that featured AM on one side and a blank on the other side. After 3 min the number of larvae on either side was determined, and the preference for the odor AM in the paired and unpaired groups calculated:

1 $${\mathrm {Odor}}{\hbox{-}}{\mathrm {Pref}}=({{\rm number}} \ {{\rm on}} \ {{\rm AM}} \ {{\rm side}}-{{\rm number}} \ {{\rm on}} \ {{\rm blank}} \ {{\rm side}})/{{\rm total}} \ {{\rm number}}$$

From the difference in preference between the paired-trained and unpaired-trained groups the PI was calculated

2 $${{\rm PI}} = \left( {{{\rm Odor} {\hbox{-}} {\rm Pref}}_{{{\rm Paired}}}-{{\rm Odor} {\hbox{-}} {\rm Pref}}_{{{\rm Unpaired}}}} \right)/2$$

PI scores are thus bound between −1 and 1, with positive scores indicating appetitive and negative scores aversive memory. Each PI score (*N* = 1) is based on Odor-Pref data from 60 larvae (*N* = 30 each for the paired-trained and unpaired-trained case).

### Odor-FRU reward association experiments for the screen

For the validation of candidates from the pre-screen with more specific drivers, it was considered important to be able to observe the effector expression directly, and *UAS*-*Kir2.1::GFP* was chosen as the effector. These experiments, and the ones mentioned below, were performed at the LIN Magdeburg in the way described above except that (i) training and test for these experiments were run at 25 °C because the *Kir2.1::GFP* effector does not require heat induction; (ii) 1% agarose (NEEO Ultra-Quality; CAS: 9012-36-6; Roth, Karlsruhe, Germany) was used instead of agar; (iii) fresh Petri dishes were used for each training trial; (iv) odors were applied using custom-made Teflon containers with perforated lids to allow for evaporation of the odor (AM; CAS: 628-63-7; Merck Millipore, Darmstadt, Germany, diluted 1:20 in paraffin oil, CAS: 8042-47-5; PanReac AppliChem, Darmstadt, Germany); and (v) experiments were organized per genotype, i.e., the experiments using a given driver and its genetic controls were performed in parallel until finished, followed by the next driver and its respective genetic controls.

### Association of odor with other taste rewards

The experiments for odor-^low^salt, odor-ASP, odor-ARA, and odor-SOR reward association were performed as detailed for odor-FRU association in the previous section, except that 0.4 mol/l NaCl (salt; CAS: 7647-14-5; Roth), 10 mmol/l ASP (CAS: 56-84-8, Sigma-Aldrich Chemie GmbH, Munich, Germany), 2 mol/l ARA (CAS: 10323-20-3; Sigma-Aldrich Chemie GmH), and 2 mol/l SOR (CAS: 50-70-4; Roth) were used, respectively.

### Innate odor preference

Groups of 30 experimentally naïve larvae were placed in the middle of a Petri dish that featured the odor on one side and a blank on the other side. After 3 min the number of larvae on either side was determined, and the preference for AM was calculated according to Eq. (1).

### Innate FRU, ASP, and low-salt preference

Split Petri dishes were prepared by filling one-half with 1% agarose (blank), and the other half with agarose plus 2 mol/l FRU. Thirty larvae were collected and placed in the middle of these dishes, and after 3 min their numbers were scored on either side, or on a 10 mm middle zone. The preference for fructose was calculated as:

3 $${{\rm FRU} {\hbox{-}} {\rm Pref}} = \left( {{{\rm number}}\,{{\rm on}}\,{{\rm FRU}}\,{{\rm side}}-{{\rm number}}\,{{\rm on}}\,{{\rm blank}}\,{{\rm side}}} \right)/{{\rm total}}\,{{\rm number}}$$

Positive values thus indicate a preference for, and negative values an avoidance of FRU.

Low-salt and ASP preferences were determined in the same way, except that 0.4 mol/l NaCl and 10 mmol/l ASP were used, respectively.

### Genotypes and transgenes for the pre-screen

For the pre-screen we used the double-heterozygous offspring from crosses of Gal4 strains from the Janelia collection^57^ (available from the Bloomington Stock Center or the Vienna Drosophila Resource Center; Supplementary Table 2 and Supplementary Table 3) to drive a temperature-sensitive dynamin transgene from the *UAS-shi*^*ts*^ effector^98^ (Bloomington Stock Center no. 44222). In the Gal4-expressing cells, this allows synaptic output to be acutely disabled in a temperature-dependent way.

The Gal4 strains used feature insertions of a given enhancer fragment combined with the *Gal4* gene as the driver construct; these driver constructs were inserted into an attp2 landing site. Therefore, a strain carrying this attp2 landing site but no Gal4 construct (empty) was crossed to *UAS-shi*^*ts*^ to obtain baseline, control memory scores for the pre-screen (Ctrl). Note that the same Gal4 construct (respectively combined with a different enhancer fragment) likewise inserted at the attp2 site is used in all strains to drive the *UAS-shi*^*ts*^ construct. Adverse effects of the attp2 site, the Gal4 construct, or of a leakiness of the *UAS-shi*^*ts*^ construct would have affected all screened strains, and thus cannot account for differences in memory scores between them.

### Genotypes and transgenes for the screen

For the validation of candidate cells from the pre-screen a collection of 12 split-Gal4 intersection strains was generated (Supplementary Table 4). These express either the transcription activation domain (p65ADZp) or the DNA-binding domain (ZpGAL4DBD) of GAL4, each under the control of one of the selected enhancer fragments^59, 60^. The landing sites for the p65ADZp and the ZpGAL4DBD construct were attp40 and attP2, respectively. Upon combining the p65ADZp and ZpGAL4DBD strains by classical genetics, a functional Gal4 protein is thus expressed only in the intersection of the two strains^59, 60^. Specificity was confirmed by crossing the split-Gal4 drivers to *UAS-GFP* from ref. ^60^ (Bloomington Stock Center no. 32185 and 32186) or *UAS*-*Kir2.1::GFP* (*5xUAS-DCSP-eGFPKir2.1* at VK00005 as landing site; available upon request) and inspecting expression under a Zeiss LSM 510 and/or a Leica DM 6000 CS confocal microscope.

Split-Gal4 strains with a confirmed expression pattern were crossed to *UAS*-*Kir2.1::GFP* as the effector, an inward-rectifying potassium channel that silences expressing neurons by preventing them from spiking^99^. The advantage of this effector relative to *UAS-shi*^*ts*^ is that the expression pattern and therefore the manipulated cells can be directly verified via the GFP tag. For the single-cell analyses we were aiming for, this advantage appeared to outweigh the disadvantage of it being constitutive in effect. As driver controls, the split-Gal4 strains were crossed to the wild-type strain (CS_MH_, available upon request) that had been used as the genetic background for the *UAS*-*Kir2.1::GFP* strain (driver Ctrl). As an effector control, a strain homozygous for both the attP40 and attP2 landing sites, yet without a Gal4 domain inserted (empty), was crossed to *UAS*-*Kir2.1::GFP* (effector Ctrl).

### Genotypes and methods for optophysiology

The indicated driver strains were crossed to *UAS-ChR2-XXL* as the effector^100^ (Bloomington Stock Center no. 58374). Double-heterozygous progeny was used for activation; larvae heterozygous for either the Gal4 or the effector were used as genetic controls. Optogenetic experiments were performed in a dark custom-built box equipped for illumination from a blue LED light table (wavelength: 470 nm; intensity: 1.2 µW/mm^2^). For the learning experiments, one group of larvae received the odor presentation during blue light illumination alternated with blank trials without odor and without illumination (AM+/blank), whereas a reciprocally trained group of larvae received the odor unpaired with light stimulation (AM/+). All other aspects of the learning task were performed as described for odor-FRU learning. The same type of experiment was also performed using a tomato-tagged version of *UAS-ChR2-XXL* (*UAS-ChR2-XXL::tdtomato*, kindly provided by R. Kittel, University of Würzburg) under the same conditions as the experiments with *UAS-ChR2-XXL*, as well as using *UAS-Chrimson* as the effector^101^ (Bloomington Stock Center no. 55134). For the *UAS-Chrimson* experiments, 630 nm red light at an intensity of 3.5 µW/mm^2^ was used, and the odor for training was ethyl acetate (CAS: 141-78-6; Sigma-Aldrich Chemie GmbH) diluted in distilled water at a concentration of 10^−4^.

### Genotype for movies

To obtain the data shown in Movies 1-5, we generated recombinations of nSyb-Gal4 as driver (Bloomington Stock Center no. 51635) and UAS-mCherryCAAX as effector (Bloomington Stock Center no. 59021) and viewed fluorescence with a confocal microscope (for more details see end of movies).

### Locomotor footprint of memories established by DAN activation

For the experiments shown in Fig. 5e–g the larval behavior was video-tracked and analyzed as described in detail in ref.^53^. In brief, two aspects of larval chemotaxis were analyzed. First, based on the number of head casts (#HC) performed while either heading away from the odor of when heading towards it, the modulation of HC rate was determined:

4 $$\begin{array}{ccccc}\\ {{\rm HC} \, {\rm rate} {\hbox{-}} {\rm modulation}} &= \left( {\# {{\rm HC}}/{{\rm s}}\left( {{{\rm away}}} \right) - \# {{\rm HC}}/{{\rm s}}\left( {{{\rm towards}}} \right)} \right)\\ & /\left( {\# {{\rm HC}}/{{\rm s}}\left( {{{\rm away}}} \right) + \# {{\rm HC}}/{{\rm s}}\left( {{{\rm towards}}} \right)} \right)\\ \end{array}$$

This measure yields positive scores for attraction, i.e., when larvae systematically perform more HCs while heading away from the odor (i.e., when odor concentration decreases) than while heading toward it (i.e., when odor concentration increases). Conversely, it yields negative scores for aversion.

Second, based on the absolute heading angle before a HC α and the absolute heading angle after it β, the modulation of HC direction is measured by the reorientation per HC:

5 $$\begin{array}{ccccc}\\ {{\rm Reorientation}}\,{{\rm per}}\,{{\rm HC}} = & {\alpha}- {\beta}\\ \end{array}$$

The heading angle describes the orientation of the animal’s head relative to the odor, with absolute heading angles of 0° or 180°, for example, indicating that the odor is to the front or to the rear of the larvae, respectively. This measure thus yields positive scores for attraction, i.e., when the HC directs the larvae towards rather than away from the odor target, whereas it yields negative scores for aversion.

### Timing-dependent reversal of DAN-i1 reinforcement

For the experiment shown in Fig. 6, a modified optophysiological activation protocol was used, varying the inter-stimulus interval (ISI) between odor and DAN-i1 activation. Double-heterozygous progeny of SS00864 split-Gal4 crossed to *UAS-ChR2-XXL* were used; animals heterozygous for either construct were used as genetic controls. The larvae were placed in the middle of an agarose-filled Petri dish that was covered with a lid. For the case of forward conditioning with an ISI of −30 s as an example (Fig. 6d), the paired training consisted of presenting the odor at time 3.5 min for 30 s by replacing the standard, blank lid with a modified lid equipped with odor-loaded filter papers as for the pre-screen experiments. At 4 min, and thus at a 30-s ISI relative to odor onset, DAN-i1 was activated for 30 s by blue light. Then the larvae were left untreated until 12 min, when the next of a total of three such training trials began. Only after the end of the third training trial were the larvae collected, moved to a fresh test Petri dish, and scored for their odor preference according to Eq. (1). Larvae of the unpaired groups were treated the same, except that odor presentation was temporally removed from DAN-i1 activation by 4 min. Performance indices were then calculated according to Eq. (2). The critical variable of the experiment was that the ISI in the paired groups varied between experimental conditions: for the case of backward conditioning with an ISI of 30 s as an example, DAN-i1 activation came first and was followed by odor presentation after 30 s. In order to equate paired and unpaired groups for manipulations of the lid, exchanges of blank lids were included at the time points of odor presentation for the corresponding other group.

### Immunohistochemistry

In all, 877 Gal4 driver strains preselected for expression in the central nervous system of stage 3 *Drosophila* larvae^57^ (http://flweb.janelia.org/cgi-bin/flew.cgi) were screened for coverage of MBE neurons. Anatomical screening was further refined by systematic multicolor flp-out analysis of all 877 Gal4 driver lines^57, 58^ (Fig. 2, Supplementary Table 1, 2 and 3). In addition, expression patterns of split-Gal4 strains (Figs. 3, 4, and 10; Supplementary Fig. 8, Supplementary Fig. 10, Supplementary Fig. 15, Supplementary Fig. 16, and Supplementary Fig. 21) were anatomically verified. Specifically, larval brains of the indicated genotypes were dissected in phosphate-buffered saline (PBS) and fixed for 1 h in 4% paraformaldehyde at room temperature. After multiple rinses in PBS with 1% Triton X-100 (PBT) and incubation in 3% normal donkey serum in PBT (2 h at room temperature), samples were incubated over two nights at 4 °C with the indicated primary antibodies. After multiple rinses in PBT, brains were incubated for 2 days at 4 °C with the indicated secondary antibodies. For mounting, the brains were rinsed multiple times in PBT, transferred on a poly-l-lysine-coated cover glass, dehydrated through a graded ethanol series, cleared in xylene, and embedded in the DPX Mountant (Sigma). Detailed information including step-by-step protocol and movies can be found on the HHMI Janelia Research Campus website (https://www.janelia.org/project-team/flylight/protocols). Immunolabeled larval nervous systems were imaged on a Zeiss 510 confocal microscope.

To verify *UAS*-*Kir2.1::GFP* effector gene expression a different protocol was applied, modified after^37^. Larval brains of the indicated genotypes were dissected in Ringer solution. The brains were fixed in 4% formaldehyde (Merck) in PBS for 25 min. After several washes with PBT (PBS with 3% Triton X-100, Sigma-Aldrich, St. Louis, MO), the brains were blocked with 5% normal goat serum (Jackson ImmunoResearch Laboratories, West Grove, PA) in PBS for 2 h and then incubated for 1 day with the indicated primary antibodies at 4 °C. After several washes with PBS, the indicated secondary antibodies were applied for 1 day at 4 °C. Finally, the brains were again washed with PBS and mounted in Vectashield (Vector Laboratories).

### Antibodies

To visualize the expression patterns of split-Gal4 strains, mouse anti-neuroglian (1:50; BP-104; Developmental Studies Hybridoma Bank), rat anti-N-cadherin (1:50; DN-Ex #8; Developmental Studies Hybridoma Bank), and rabbit anti-GFP (1:1000; Life Technologies A11122) were used as primary antibodies. Alexa Fluor 568 donkey anti-mouse IgG (1:500; Life Technologies A10037), Alexa Fluor 647 donkey anti-rat IgG (1:500; Jackson ImmunoResearch 712-605-153), and fluorescein isothiocyanate-conjugated donkey anti-rabbit (1:500; Jackson ImmunoResearch 711-095-152) were used as secondary antibodies.

To visualize the morphology of individual neurons the multicolor flp-out^58^ technique was applied. Primary antibodies included mouse anti-neuroglian (1:50; BP-104; Developmental Studies Hybridoma Bank), rabbit anti-HA Tag (1:500; Cell Signal Technologies 3724S), and rat anti-FLAG Tag (DYKDDDDK Epitope Tag; 1:500; Novus Biologicals NBP1-06712). Secondary antibodies were Alexa Fluor 488 donkey anti-mouse IgG (1:500; Life Technologies A10037), DyLight 549 goat anti-rabbit (1:800; Rockland Antibodies and Assays 611-142-002), and Alexa Fluor 594 donkey anti-rat IgG (1:500; Jackson ImmunoResearch 712-585-150). In addition, the protocol included a post-secondary antibody wash using PBT, with blocking using normal mouse serum (1:20 in PBT), and additional incubation with Alexa Fluor 647 mouse anti-V5 Tag (1:200; AbD Serotec MCA1360A647) overnight at 4 °C.

To verify *UAS*-*Kir2.1::GFP* effector gene expression, rabbit anti-GFP (1:1000; Invitrogen A11122) and mouse anti-FASII (1:50; DSHB) were used as primary antibodies, and Alexa Fluor 488 goat anti-rabbit (1:200; Invitrogen A11034) and Cy3 donkey anti-mouse (1:200; Jackson ImmunoResearch Laboratories 715-165-150) as secondary antibodies.

### Electron microscopy

We took advantage of the electron microscopy reconstruction of the MB intrinsic neurons, the KCs, and all their pre- and postsynaptic partners, from a 6-h-old stage 1 larva^7^. Images were taken at 3.8 × 3.8 × 50 nm resolution. Reconstruction methods were previously described in detail^7^.

### Data availability

All data are included in Supplementary Data File 1, or are available from the authors upon request. Connectomic analyses are based on data available with ref. ^7^.

## Electronic supplementary material

Supplementary Information(PDF 31820 kb)

Description of Additional Supplementary Files(PDF 76 kb)

Supplementary Movie 1

Supplementary Movie 2

Supplementary Movie 3

Supplementary Movie 4

Supplementary Movie 5

SupplementaryMovie 6

Supplementary Movie 7

Supplementary Data 1(XLSX 245 kb)
