# Supplementary material for: Functional architecture of reward learning in mushroom body extrinsic neurons of larval Drosophila
Source: Nat Commun. 2018 Mar 16;9:1104. doi: 10.1038/s41467-018-03130-1 (PMC5856778; doi:10.1038/s41467-018-03130-1)
Supplement: Supplementary file 1 — Supplementary Information(PDF 31820 kb) [file 41467_2018_3130_MOESM1_ESM.pdf]

Table 1: Defining features of mushroom body extrinsic neurons (MBEs)

MB compartment	Cell	MBE class	Cell body location	Major presynaptic terminals @ (bold: MB compartment)	Major postsynaptic region(s) @ (bold: MB compartment)	Interhemispheric cross talk? (with MB-to-MB connection?)	Lineage	Transmitter	Synonym cell name	Reference
CX*	OAN-a1	sMBIN	SEZ maxillary segment	AL (c + i), CX (c + i), CPL (c + i)	SEZ (both hemispheres)	Yes (No)	Not determined	OA/TA	sVUMmx1	Selcho et al. 2014
	OAN-a2	sMBIN	SEZ mandibular segment	AL (c + i), CX (c + i), CPL (c + i)	SEZ (both hemispheres)	Yes (No)	Not determined	OA/TA	sVUMmd1	Selcho et al. 2014
	MBON-a1	sMBON	PDM	CPM (c + i), CPI (c + i)	**CX (i)**	Yes (No)	CPv2/3	ChAT	odd	Slater et al. 2015
	MBON-a2	sMBON	PDM	CPM (c + i), CPI (c + i)	**CX (i)**	Yes (No)	CPv2/3	ChAT	odd	Slater et al. 2015
IP	MBIN-b1	sMBIN	ADL	**IP (c)**	CPL (i), CPM (i), BPM (i)	Yes (No)	DPLd			
	MBIN-b2	sMBIN	ADL	**IP (c)**	CPL (i), CPM (i), BPM (i)	Yes (No)	DPLd			
	MBON-b1	sMBON	PVL	CPL (i), CPI (i)	**IP (i)**	No (No)	BLV a3/4	GABA	BL	Pauls et al. 2010, Eichler et al. 2017
	MBON-b2	sMBON	PVL	CPL (i), CPI (i)	**IP (i)**	No (No)	BLV a3/4	GABA	BL	Pauls et al. 2010, Eichler et al. 2017
	MBON-b3***	sMBON	PVL	CPL (i), CPM (c + i), CPI (c + i)	**IP (i)**	Yes (No)	CPv2/3			
LP	DAN-c1	sMBIN	PVL	**LP (c + i)**	DP (i), DA (i)	Yes (Yes)	CPd2/3	DA	DL1-5	Selcho et al. 2009, Eichler et al. 2017
	MBIN-c1***	sMBIN	PVL	**LP (c + i)**	DP (i), DA (i)	Yes (Yes)	CPd2/3			
	MBON-c1	sMBON	PDL	CPL (i), BPM (i), CPM (i)	**LP (i)**	No (No)	BLDc	ChAT		Eichler et al. 2017
	MBON-c2	sMBON	PDM	SEZ (i), TG (i)	**LP (i)**, BPL (i); BPM (i)	No (No)	DPM l1	DA	DM1	Selcho et al. 2009
LA	DAN-d1	sMBIN	PVL	**LA (c + i)**	DP (i), BC (i), CPM (i)	Yes (Yes)	CPd2/3	DA	DL 1-4	Selcho et al. 2009, Eichler et al. 2017
	MBIN-l1	sMBIN	PVL	**LA (c)**	BC (i)	Yes (No)	BLV a3/4			
	MBON-d1	sMBON	ADL	CPM (c + i)	**LA (i)**	Yes (No)	DAL CM-1/2	GABA	la type	Pauls et al. 2010, Eichler et al. 2017
	MBON-p1	sMBON	ADL	BPM (i)	**LA (i)**, CPI (i), DP (i), DA (i)	No (No)	DAL CM-1/2			
	MBON-d2***	sMBON	AVM	CPM (i)	**LA (i)**	No (No)	BAmd2			
UVL	OAN-e1	sMBIN	PVL	**UVL (i)**	DA (i), DP (i)	No (No)	CPd2/3	OA		Eichler et al. 2017
	MBIN-e2	sMBIN	PVL	**UVL (c + i)**	DA (i), DP (i)	Yes (Yes)	CPd2/3	DA?	vl 3 type, DL1-1	Pauls et al. 2010, Selcho et al. 2009 - but see also Eichler et al. 2017
	MBON-e1	sMBON	PVL	DA (i), DP (i)	**UVL (i)**	No (No)	CPd2/3	ChAT		Eichler et al. 2017
IVL	DAN-f1	sMBIN	PVL	**IVL (c + i)**	DA (i), DP (i), CPL (i)	Yes (Yes)	CPd2/3	DA	vl 2 type, DL 1-2	Pauls et al. 2010, Selcho et al. 2009, Eichler et al. 2017
	MBON-f2	sMBON	ADL	BC (c + i)	**IVL (i)**	Yes (No)	DAL cl2			
	MBON-f1***	sMBON	PVL	SEZ (c + i)	**IVL (i)**, DA (i), DP (i), CPL (i)	Yes (No)	CPd			
LVL	DAN-g1	sMBIN	PVL	**LVL** (c), CA (c), CPI (c)	DA (i), BC (i), DP (i)	Yes (No)	CPd2/3	DA	DL 1-3	Selcho et al. 2009, Eichler et al. 2017
	OAN-g1	sMBIN	SEZ maxillary segment	**IVL** (c), DA (c), DP (c), CPL (c)	SEZ (c + i)	Yes (No)	Not determined	OA/TA	sVPMmx	Selcho et al. 2014
	MBON-g1	sMBON	AVL	CPM (i)	**LVL (i)**	No (No)	DAL-V 2/3	GABA	vl1 type	Pauls et al. 2010, Eichler et al. 2017
	MBON-g2	sMBON	AVL	CPM (i)	**LVL (i)**	No (No)	DAL-V 2/3	GABA	vl1 type	Pauls et al. 2010, Eichler et al. 2017
SHA	DAN-h1	sMBIN	ADL	**SHA (c + i)**	DA (i), DP (i)	Yes (Yes)	DAL CM-1/2	DA	pPAM1	Rohwedder et al. 2016
	MBON-h1	sMBON	AVL	CPM (c + i)	**SHA (c + i)**	Yes (Yes)	DAL-V 2/3	GABA/GLUT	APBL	Pauls et al. 2010, Eichler et al. 2017
	MBON-h2	sMBON	AVL	CPM (c + i)	**SHA (c + i)**	Yes (Yes)	DAL-V 2/3	GABA/GLUT	APBL	Pauls et al. 2010, Eichler et al. 2017
UT	DAN-i1	sMBIN	ADL	**UT (c + i)**	DA (i), DP (i)	Yes (Yes)	DAL CM-1/2	DA	pPAM3	Rohwedder et al. 2016, Eichler et al. 2017
	MBON-i1	sMBON	ADM	DA (c + i), CPM (c + i)	**UT (c + i)**	Yes (Yes)	DAM-d1	GLUT		Eichler et al. 2017
IT	DAN-j1	sMBIN	ADL	**IT (c+ i)**	DA (i), DP (i)	Yes (Yes)	DAL CM-1/2	DA	pPAM4	Rohwedder et al. 2016, Eichler et al. 2017
	MBON-j1	sMBON	ADM	DA (i), DP (i), CPL (i)	**IT (c + i)**	Yes (Yes)	DAM-d1	GLUT		Eichler et al. 2017
	MBON-j2	sMBON	ADL	DA (c), DP (c), CPL (c)	**IT (i)**	Yes (No)	DAL CM-1/2	GLUT		This study, Figure S21
LT	DAN-k1	sMBIN	ADL	**LT (c + i)**	DA (i), DP (i), BC (i)	Yes (Yes)	DAL CM-1/2	DA	ma type, pPAM2	Pauls et al. 2010, Rohwedder et al. 2016, Eichler et al. 2017
	MBON-k1	sMBON	ADM	DA (c + i)	**LT (c + i)**	Yes (Yes)	DAM-d1	GLUT		Eichler et al. 2017
MI	MBIN-m1	mMBIN	PVL	**ML***** (c + i), IVL**	DA (i)	Yes (Yes)	CPd2/3			
	APL	mMBIN/MBON	AVL	**CX (i)**	**UT (i), LT (i), UVL (i), IVL (i), LVL (i), LA (i),** DA (i)	No (No)	BLV a3/4	GABA	Larval APL	Masuda-Nakagawa et al. 2014, Eichler et al. 2017
	MBON-e2	mMBON	ADM	CPM (c + i), CPI (c + i)	**UVL (i), IVL (i), LVL (i),**	Yes (No)	DAM-d1	GLUT		Eichler et al. 2017
	MBON-m1	mMBON	ADL	BC (c), BPM (c)	**IVL (i), LVL (i), LA (i),** DA (i)	Yes (No)	DAL CM-1/2	GABA		Eichler et al. 2017
	MBON-n1	mMBON	AVM	DA (i), BC (i)	**UVL (i), IVL (i), LVL (i),** DA (i), DP (i), BC (i)	No (No)	BA md1			
	MBON-d3	mMBON	ADM	CPI (c + i), CPM (c + i)	**UVL (c + i), IVL (c + i)**	Yes (Yes)	DAM-d1			

MB compartments and MBE class

**CX**	Calyx
**IP**	Intermediate peduncle
**LP**	Lower peduncle (spur**)
**LA**	Lateral appendix
**UVL**	Upper vertical lobe
**IVL**	Intermediate vertical lobe
**LVL**	Lower vertical lobe
**SHA**	Shaft of medial lobe
**UT**	Upper toe of medial lobe
**IT**	Intermediate toe of medial lobe
**LT**	Lower toe of medial lobe
**ML**	Medial lobe
**VL**	Vertical lobe
sMBE	MBE with single-compartment innervation
mMBE	MBE with multiple-compartment innervation

Orientation for cell body location

A: anterior
P: posterior
V: ventral
D: dorsal
L: lateral
M: medial

Neuropils/nervous system compartments

AL	antennal lobe (BA****)
SEZ	subesophageal zone (sbg****)
CPM	centroposterior medial compartment
CPI	centroposterior intermediate compartment
CPL	centroposterior lateral compartment
BA	basoanterior compartment
BPM	basoposterior medial compartment
DA	dorsoanterior compartment
DP	dorsoposterior compartment
BC	basocentral compartment
CA	centroanterior compartment
CPI	centroposterior intermediate compartment
TG	thoracic ganglion
BPL	basoposterior lateral compartment
i	ipsilateral projection, relative to cell body location
c	contralateral projection, relative to cell body location

*Ascending sensory interneurons, such as the olfactory PNs, are not included
**Synonym according to Selcho et al. 2009
***Not included in behavioral screen
****Synonym according to Younossi-Hartenstein et al. 2003
*****Innervated compartments within ML cannot be clearly discerned

Table 2: Matrix of which Gal4 driver strain(s) include(s) which MBE neuron

MBE neuron…	… covered in driver strain(s)
OAN-a1	R34A11
OAN-a2	R34A11
MBON-a1	R28A09, R36G04, R37D06, R47C08, R52E12, R64F07, R93G12
MBON-a2	R28A09, R36G04, R37D06, R47C08, R52E12, R64F07, R93G12
MBIN-b1	R14B11, R64E03, R78G08
MBIN-b2	R14B11, R64E03, R78G08
MBON-b1	R12C03, R20H11, R21D02, R22A08, R27F02, R86A06
MBON-b2	R12C03, R20H11, R21D02, R22A08, R27F02, R86A06
MBON-b3	R30E10*, VT17749*
DAN-c1	R30E11, R30G04, R64E03, R76C04, R95H02
MBIN-c1	R64E03, R72G06*
MBON-c1	R20F01, R74B11, R92H01, R94E06
MBON-c2	R12C11
DAN-d1	R21D02, R22A08, R22H04, R30E08, R30F04, R38E08, R64E03, R76C04, R78E04
MBIN-l1	R12C03, R20B12, R20C09, R21A06, R47C08, R47H03, R48D11, R48F09, R52H01, R58F03, R95A11
MBON-d1	R14B11, R20F01, R37G09, R49C08, R84D07
MBON-p1	R15D04, R17B02
MBON-d2	R54A09*, R87G02*, R89B06*, VT32899*
OAN-e1	R24A12, R28A12, R46F09*, R49D11*, R52G03, R64E03, R75F01*
MBIN-e2	R12C03, R14E06, R64E03, R72B05
MBON-e1	R74B11
DAN-f1	R10B07, R21D02, R37D06, R64E03, R76F05, R95H02
MBON-f2	R10B07, R36B06, R42F08, R64A11
MBON-f1	R48E11*
DAN-g1	R10D02, R14E06, R21D02, R27F02, R30E11, R64E03
OAN-g1	R30D03
MBON-g1	R11F03, R21D06, R21D08, R23B09, R48G01, R67E07, R94E06
MBON-g2	R11F03, R21D06, R21D08, R23B09, R48G01, R67E07, R94E06
DAN-h1	R26G04, R30G08, R58E02, R64E03, R64H06, R76F05, R95H02
MBON-h1	R11C10, R11F03, R22B05, R28A10, R28F06, R32D04, R50A04, R67B01, R67E07, R74A06
MBON-h2	R11C10, R11F03, R22B05, R28A10, R28F06, R32D04, R50A04, R67B01, R67E07, R74A06
DAN-i1	R21D02, R30G08, R31C03, R48F09, R49C08, R56H09, R58E02, R64E03, R64H06, R76F05, R95H02
MBON-i1	R14C08, R15F05, R20C05, R20H11, R21C08, R21D02, R21D07, R28A12, R46E11, R53G11, R59C07, R64E01, R65A05, R65E10, R74A06
DAN-j1	R49C08, R58E02, R64E03, R64H06, R76F05
MBON-j1	R11F03, R12C11, R20H11, R21C08, R23B09, R33D07, R46E11, R74C01
MBON-j2	R24E12, R36B06, R54H12, R89G07
DAN-k1	R20H11, R21D02, R27A11, R30G08, R48F09, R64E03, R64H06, R71D01, R76F05
MBON-k1	R20H11, R21D02, R65A05, R74A06

MBIN-m1	R76F05
APL	R20H11, R21D02, R26G02, R55D08
MBON-e2	R12C11, R28A12, R43A02, R65A05
MBON-m1	R20B12, R24D12, R52H01
MBON-n1	R22B05, R29E04, R83G11
MBON-d3	R11F03, R12C11

*Not included in behavioral screen

Table 3: Matrix of which MBE neuron(s) are included in which Gal4 driver strain(s)

Driver strain…	Stock Number	… covering MBE neuron(s)	Driver strain…	Stock Number	… covering MBE neuron(s)
R10B07	48244	DAN-f1, MBON-f2	R43A02	41256	MBON-e2
R10D02	48437	DAN-g1	R46E11	50272	MBON-j1, MBON-i1
R11C10	48450	MBON-h1,h2	R46F09*	50275	OAN-e1
R11F03	48464	MBON-j1, MBON-h1,h2, MBON-g1,g2, MBON-d3	R47C08	50298	MBIN-l1, MBON-a1,a2
R12C03	45024	MBIN-l1, MBON-b1,b2, MBIN-e2	R47H03	50331	MBIN-l1
R12C11	48497	MBON-j1, MBON-e2, MBON-c2, MBON-d3	R48D11	50365	MBIN-l1
R14B11	49255	MBIN-b1,b2, MBON-d1	R48E11*	50372	MBON-f1
R14C08	48606	MBON-i1	R48F09	50377	DAN-k1, DAN-i1, MBIN-l1
R14E06	48643	DAN-g1, MBIN-e2	R48G01	50381	MBON-g1,g2
R15D04	n.a.	MBON-p1	R49C08	50418	DAN-i1, DAN-j1, MBON-d1
R15F05	48699	MBON-i1	R49D11*	38684	OAN-e1
R17B02	48754	MBON-p1	R50A04	38720	MBON-h1,h2
R20B12	48880	MBIN-l1, MBON-m1	R52E12	38837	MBON-a1,a2
R20C05	48883	MBON-i1	R52G03	38842	OAN-e1
R20C09	48886	MBIN-l1	R52H01	47638	MBIN-l1, MBON-m1
R20F01	48610	MBON-c1, MBON-d1	R53G11	50449	MBON-i1
R20H11	48918	DAN-k1, MBON-j1, MBON-i1, MBON-k1, MBON-b1,b2, APL	R54A09*	50460	MBON-d2
R21A06	n.a.	MBIN-l1	R54H12	48205	MBON-j2
R21C08	48935	MBON-j1, MBON-i1	R55D08	39115	APL
R21D02	48939	DAN-k1, DAN-i1, DAN-f1, DAN-g1, DAN-d1, MBON-i1, MBON-k1,MBON-b1,b2, AP	R56H09	39166	DAN-i1
R21D06	48942	MBON-g1,g2	R58E02	41347	DAN-h1, DAN-i1, DAN-j1
R21D07	48943	MBON-i1	R58F03	39187	MBIN-l1
R21D08	48944	MBON-g1,g2	R59C07	48217	MBON-i1
R22A08	47902	DAN-d1, MBON-b1,b2	R64A11	39289	MBON-f2
R22B05	49300	MBON-h1,h2, MBON-n1	R64E01	48230	MBON-i1
R22H04	n.a.	DAN-d1	R64E03	41351	DAN-h1, DAN-k1, DAN-i1, DAN-j1, DAN-f1, DAN-g1, DAN-c1, MBIN-c1, DAN-d1, OAN-e1, MBIN-b1,b2, MBIN-e2
R23B09	49018	MBON-j1, MBON-g1,g2	R64F07	39311	MBON-a1,a2
R24A12	49061	OAN-e1	R64H06	49608	DAN-h1, DAN-k1, DAN-i1, DAN-j1
R24D12	49080	MBON-m1	R65A05	39329	MBON-i1, MBON-k1, MBON-e2
R24E12	49084	MBON-j2	R65E10	39359	MBON-i1
R26G02	48065	APL	R67B01	39961	MBON-h1,h2
R26G04	n.a.	DAN-h1	R67E07	39444	MBON-h1,h2, MBON-g1,g2
R27A11	47908	DAN-k1	R71D01	39579	DAN-k1
R27F02	48072	DAN-g1, MBON-b1,b2	R72B05	39611	MBIN-e2
R28A09	49445	MBON-a1,a2	R72G06*	39792	MBIN-c1
R28A10	48074	MBON-h1,h2	R74A06	47398	MBON-i1, MBON-k1, MBON-h1,h2
R28A12	47909	MBON-i1, OAN-e1, MBON-e2	R74B11	41301	MBON-e1, MBON-c1
R28F06	48083	MBON-h1,h2	R74C01	39845	MBON-j1
R29E04	49486	MBON-n1	R75F01*	41304	OAN-e1
R30D03	49532	OAN-g1	R76C04	48621	DAN-c1, DAN-d1
R30E08	48099	DAN-d1	R76F05	41305	DAN-h1, DAN-k1, DAN-i1, DAN-j1, DAN-f1, MBIN-m1
R30E10*	49638	MBON-b3	R78E04	39997	DAN-d1
R30E11	48100	DAN-g1, DAN-c1	R78G08	n.a.	MBIN-b1,b2
R30F04	48614	DAN-d1	R83G11	46764	MBON-n1
R30G04	n.a.	DAN-c1	R84D07	40391	MBON-d1
R30G08	48101	DAN-h1, DAN-k1, DAN-i1	R86A06	n.a.	MBON-b1,b2
R31C03	48103	DAN-i1	R87G02*	40500	MBON-d2
R32D04	49711	MBON-h1,h2	R89B06*	40543	MBON-d2
R33D07	49893	MBON-j1	R89G07	41365	MBON-j2
R34A11	49767	OAN-a1,b	R92H01	40631	MBON-c1
R36B06	49929	MBON-j2, MBON-f2	R93G12	40667	MBON-a1,a2
R36G04	49940	MBON-a1,a2	R94E06	40687	MBON-g1,g2, MBON-c1
R37D06	47921	DAN-f1, MBON-a1,a2	R95A11	48834	MBIN-l1
R37G09	49538	MBON-d1	R95H02	40716	DAN-h1, DAN-i1, DAN-f1, DAN-c1
R38E08	50008	DAN-d1	VT17749*	200702**	MBON-b3
R42F08	50164	MBON-f2	VT32899*	203529 **	MBON-d2

* Not included in behavioral screen
** Available from Vienna Drosophila Resource Center
n.a. No longer available from Bloomington Stock Centre

Table 4: List of split-Gal4 intersection strains, their source strains, and the MBE neuron(s) covered

MBE	Classification*	ADsource strain_DBDsource strain	Strain
DAN-g1	AA	R14E06_R27G01	GMR_SS01716
MBON-g1, g2	AA	R21D06_R23B09	GMR_SS02121
DAN-h1	AAA	R76F05_R95H02	GMR_SS01696
MBON-h1, h2	AAA	R20A02_R28A10	GMR_SS01725
MBON-h1, h2	AAA	R20A02_R28A10	GMR_SS00928
DAN-i1	AA	R13D05_R36B06	GMR_SS00864
MBON-i1	AAA	R15B01_R14C08	GMR_MB141B
MBON-j1	AAA	VT057469_R18D09	GMR_SS01973
MBON-j2	AAA	R89G07_R24E12	GMR_SS00860
DAN-k1	AAA	R48F09_R27A11	GMR_SS01757
MBON-k1	AAA	VT033301_R27G01	GMR_SS01962
APL	AAA	R21D02_R55D08	GMR_SS01671

*AAA: High Expression strength, reliability, specificity
AA: Occasional, unilateral, or weak expression in other MBEs, likely without systematic effect in *en mass* behavioral assays

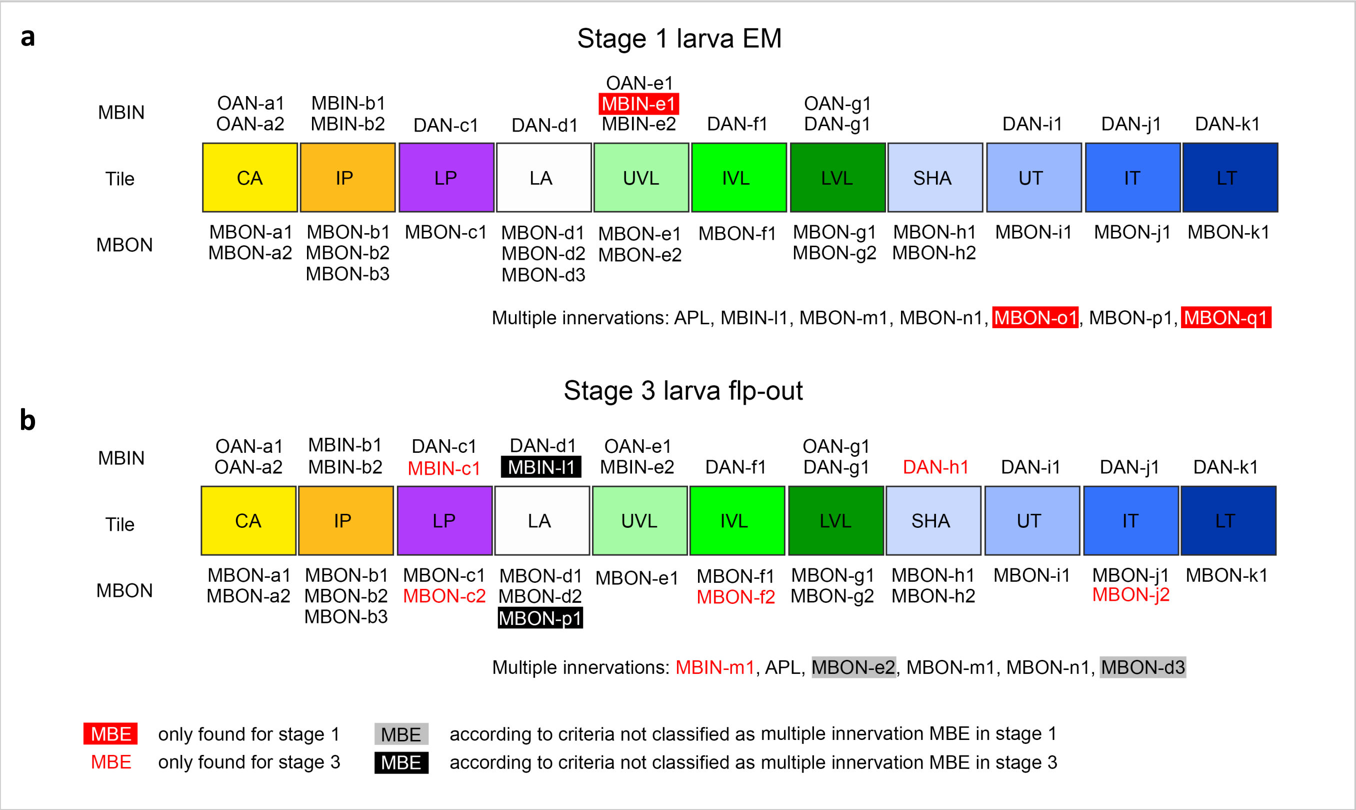

Supplementary Figure 1: Schematic organization of MBE neurons identified in stage 1 and stage 3 larvae

For both developmental stages (stage 1 in a; stage 3 in b) the compartmental organization of mushroom body extrinsic neurons (MBEs) is presented. Mushroom body input neurons (MBINs) and mushroom body output neurons (MBONs) are listed above and below each tile. MBEs that innervate multiple tiles are shown at the bottom. For stage 1 this is based on the electron microscopy reconstructions of (1), for stage 3 on light microscopy brain scans of flp-out experiments. In both developmental stages, 11 tiles can be recognized: calyx (CA); intermediate (IP) and lower peduncle (LP); lateral appendix (LA); upper (UVL), intermediate (IVL) and lower vertical lobe (LVL); the shaft (SHA), lower (LT), intermediate (IT) and upper toe (UT). Three MBEs were specifically found for stage 1 (MBIN-e1, MBON-o1 and MBON-q1; highlighted by red boxes) and six MBEs seem to be particular to stage 3 (MBIN-c1, DAN-h1, MBON-c2, MBON-f2, MBON-j2 and MBIN-m1; highlighted in red lettering).

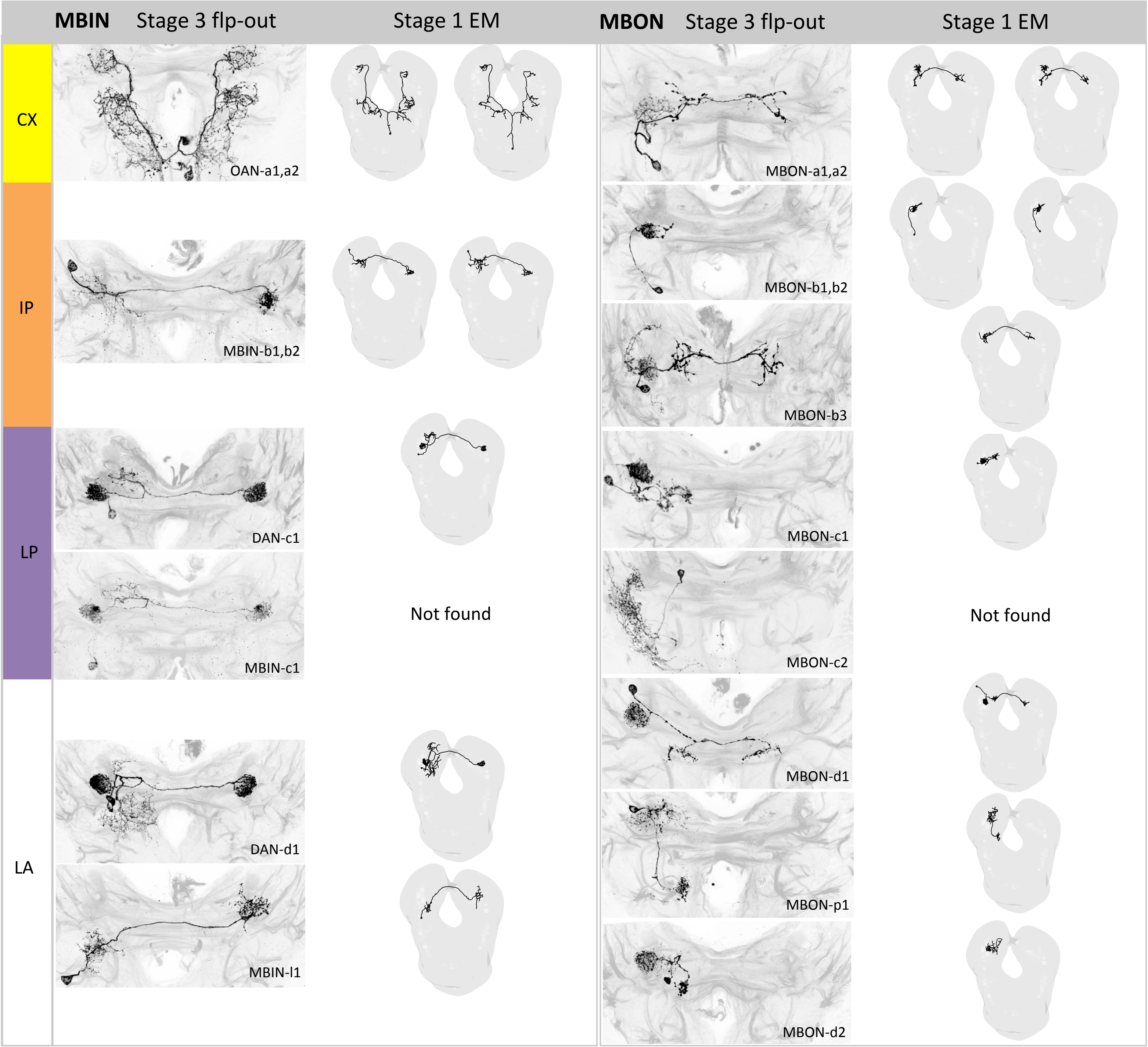

Supplementary Figure 2, ctd.

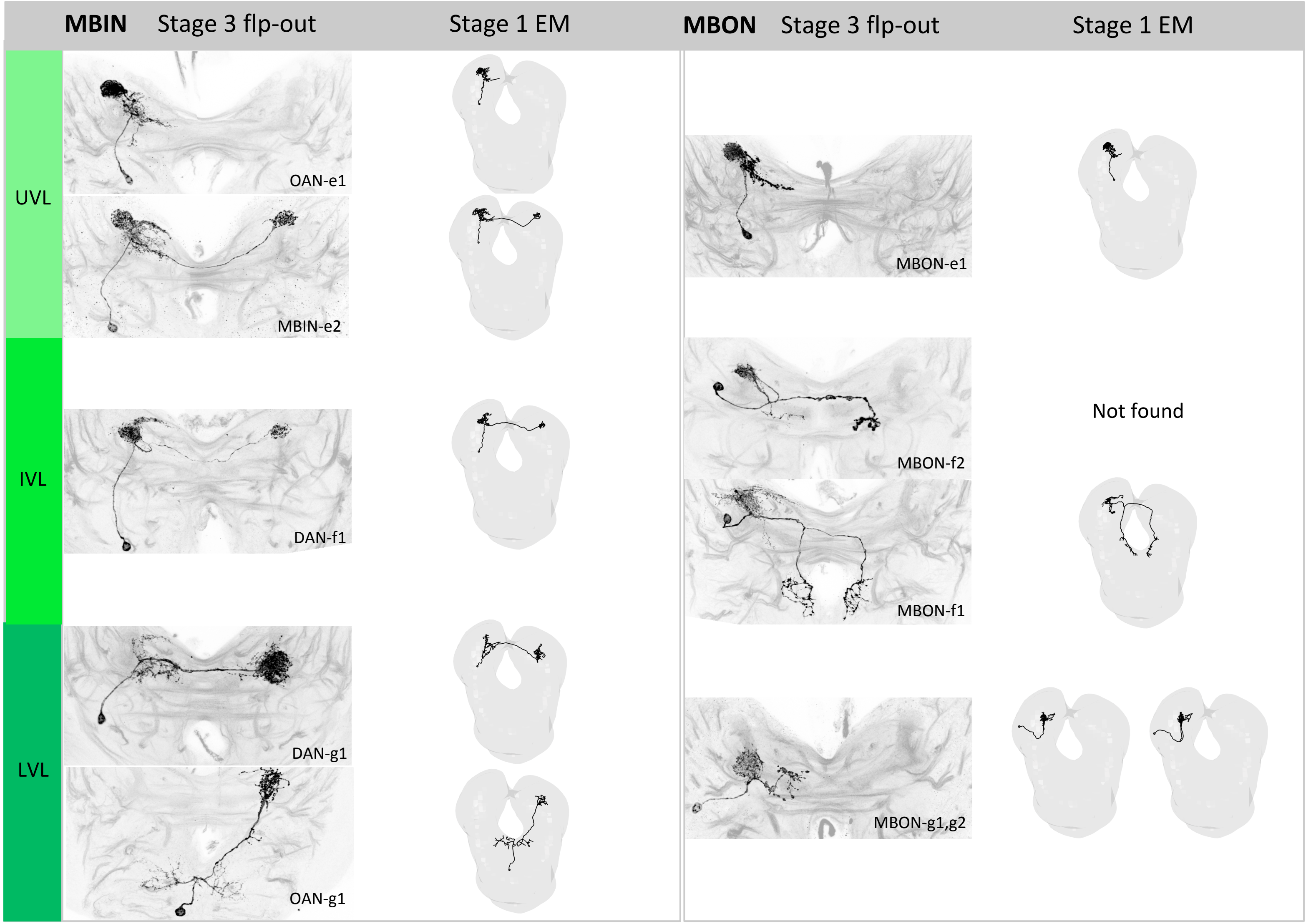

Ctd. Supplementary Figure 2

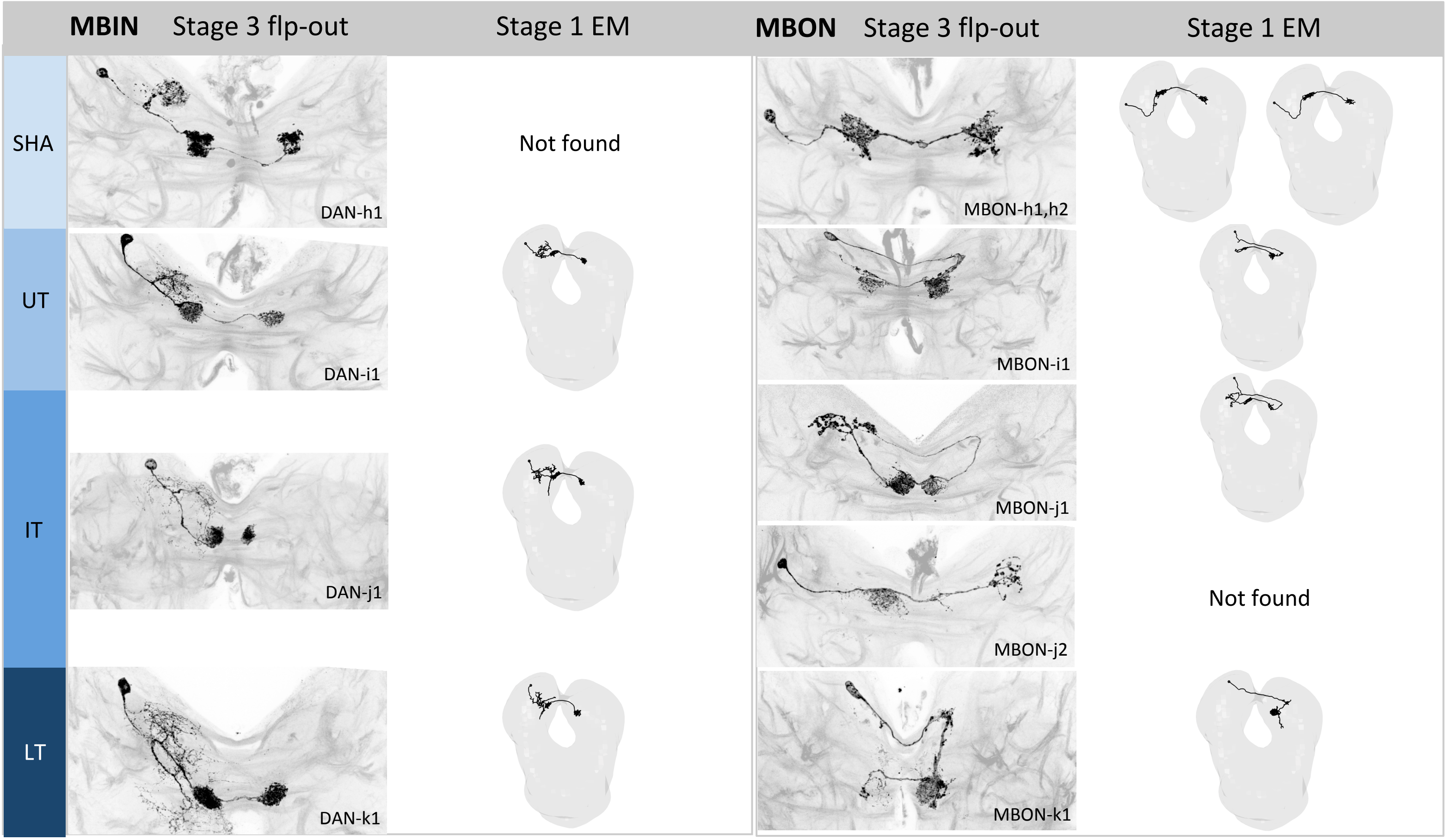

Ctd. Supplementary Figure 2

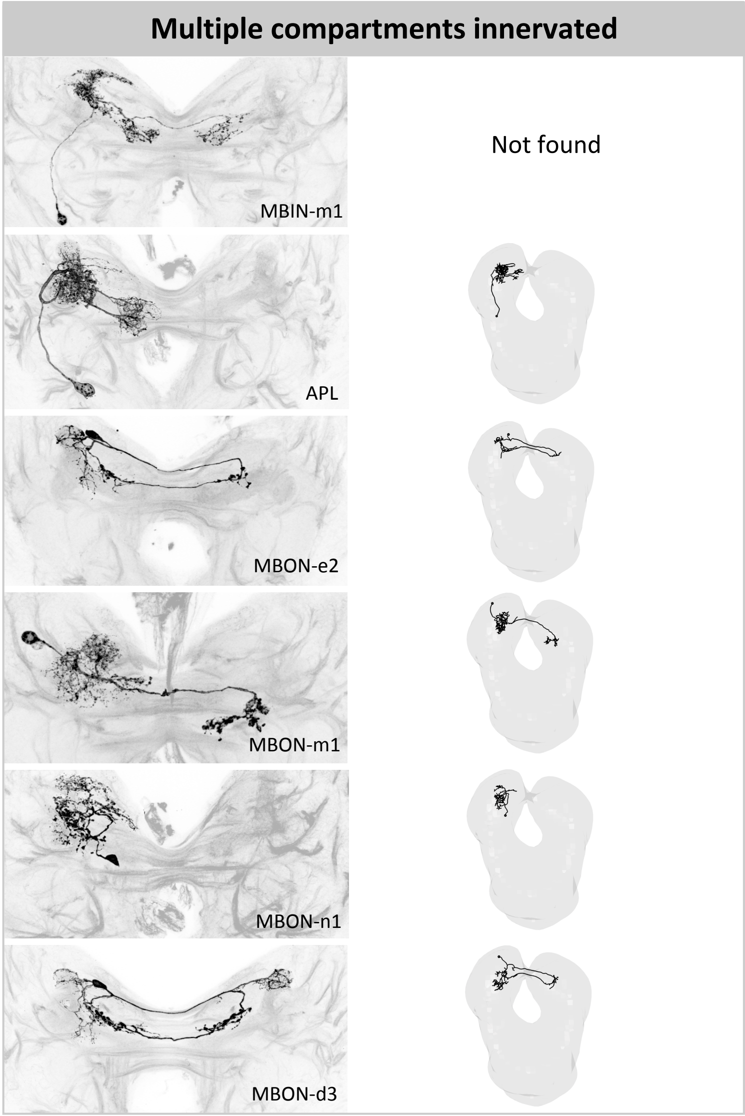

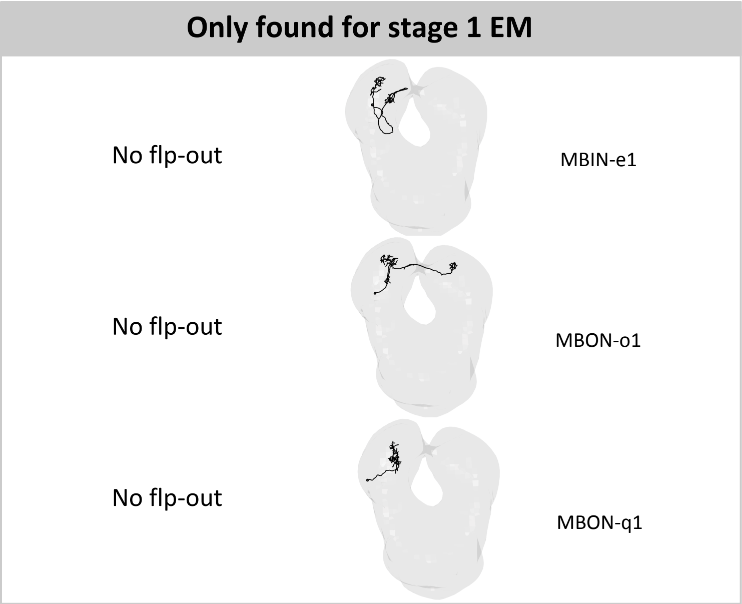

Supplementary Figure 2: Morphological organization of MBE neurons identified in stage 3 and stage 1 larvae

For both stage 3 and stage 1 the morphological organization of mushroom body extrinsic neurons (MBEs) is shown. For stage 3 this is based on light microscopy brain scans from flp-out experiments (left panels), for stage 1 on electron microscopy reconstructions from (1) (right panels). In the case of OAN-a1 and OAN-a2 two cells were obtained in the flp-out experiment; the electron microscope reconstructions of both neurons are presented in the right hand panel. For the MBE double pairs only one flp-out is shown because the ipsilateral members of the double pair cannot be distinguished by light microscopy data; as they are separately revealed in the electron microscope reconstructions, however, they are both presented in the panels to the right.

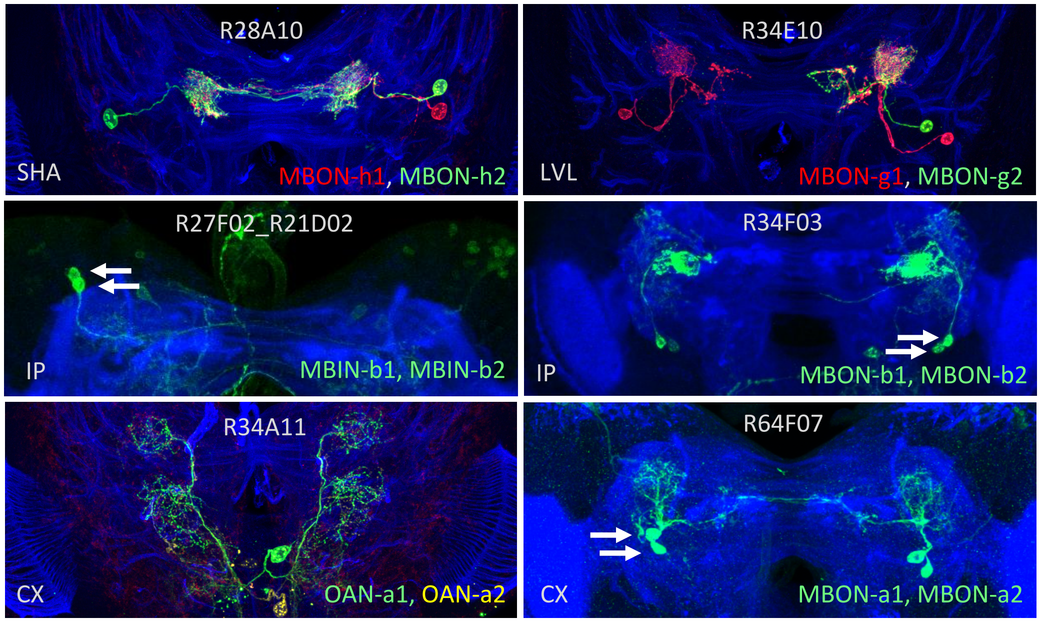

Supplementary Figure 3: MBE neuron (double) pairs and MBE symmetry

In all but in the bottom left case, flp-outs from MBE double pairs are presented, showing strongly overlapping innervation patterns of the ipsilateral neurons of the double pair. In all but the bottom left and middle left panels, the flp-outs of three or respectively four members of the double pair within the same brain directly reveal that MBE neurons are ‘paired’, i.e. are present in both hemispheres, and that the paired neurons in both hemispheres have symmetrical morphology. In the other cases, the paired nature of the MBEs and their symmetry was ascertained through independent flp-outs across brains, as well as from electron microscope reconstruction (1). The bottom left panel shows the segmentally homologous OAN-a1 and OAN-a2 neurons. Each panel shows a z-projection of the region of larval brain including the indicated MBEs. Anti-neuroglian staining (blue) provides a label for the brain. Individual neurons are shown as multi-color flp-outs in red, yellow and green depending on the expressed markers. Information about the Gal4 or split-Gal4 driver strain is given at the top of each panel. The identity of the MBEs and information about the innervated compartment is indicated within each panel. Arrows point to MBE cell bodies.

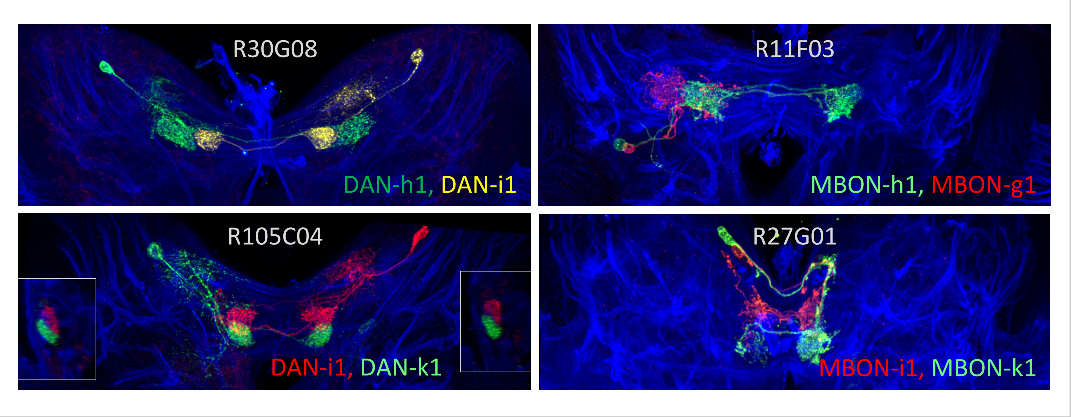

Supplementary Figure 4: MBE neurons cover compartments in a non-overlapping way
Four examples of double flp-out events of MBE neurons that innervate neighboring compartments, showing their non-overlap. Each panel shows a z-projection of the larval brain region including the indicated MBEs. Anti-neuroglian staining (blue) provides a label for the brain. Individual neurons are shown as multi-color flp-outs in red, yellow and green depending on the expressed markers. Information about the Gal4 and split-Gal4 driver strain and the identity of the MBEs is given within each panel. For DAN-i1 and DAN-k1 the insets show a sagittal view of medial lobe innervation.

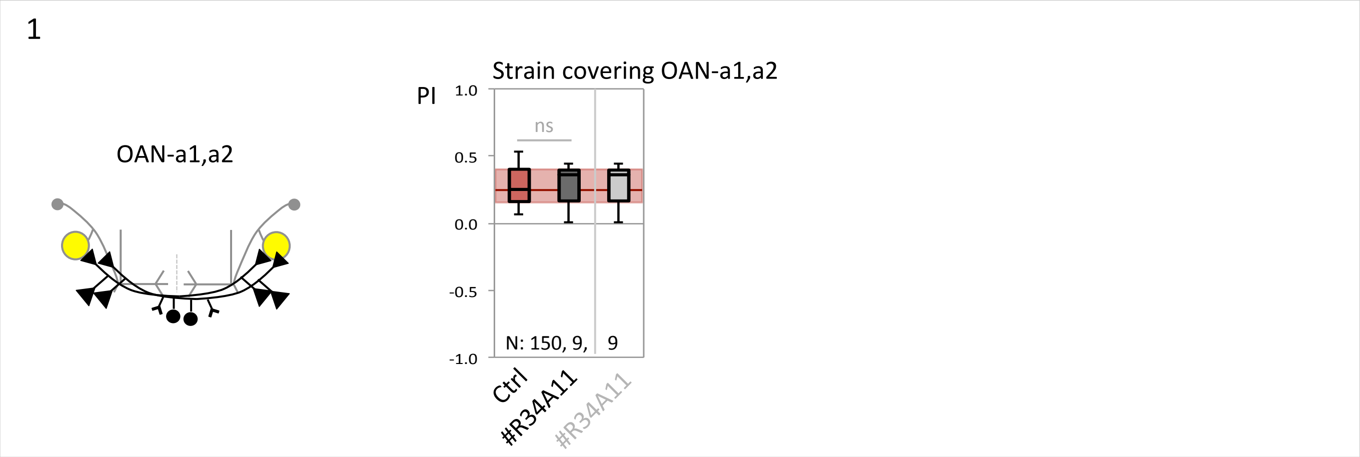

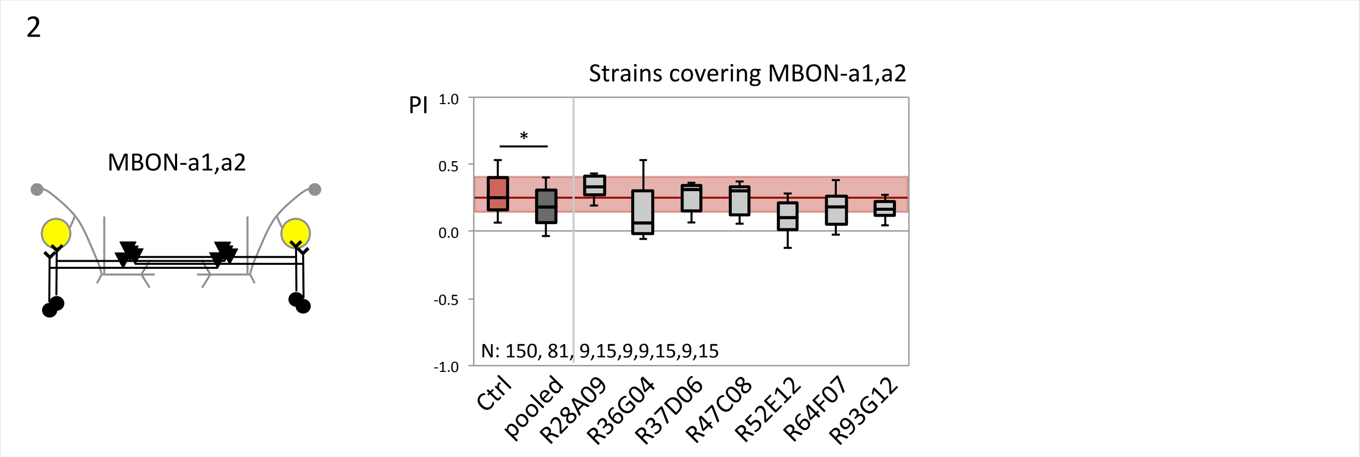

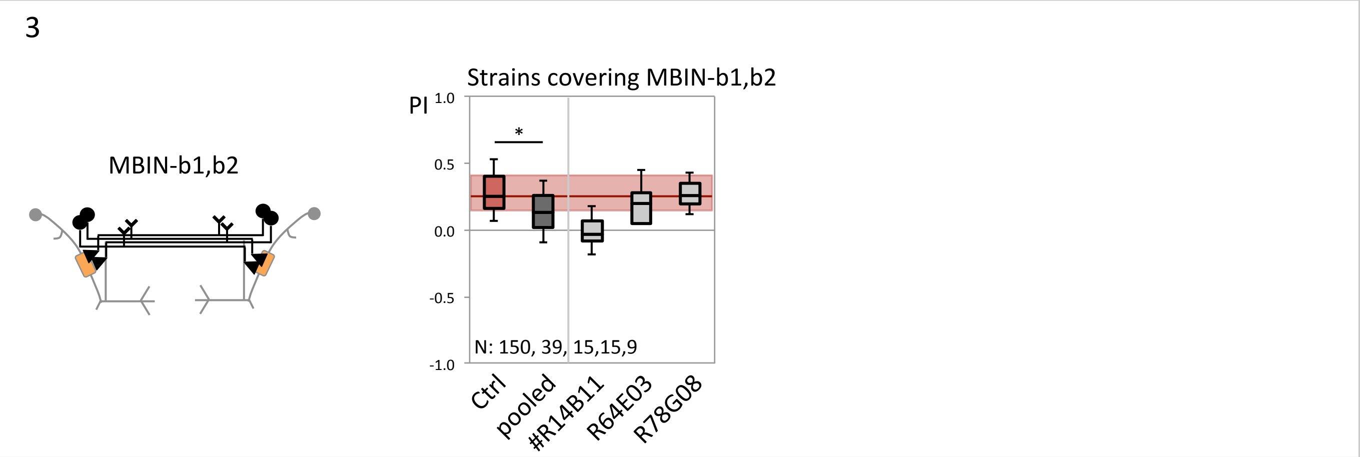

Supplementary Figure 5, ctd

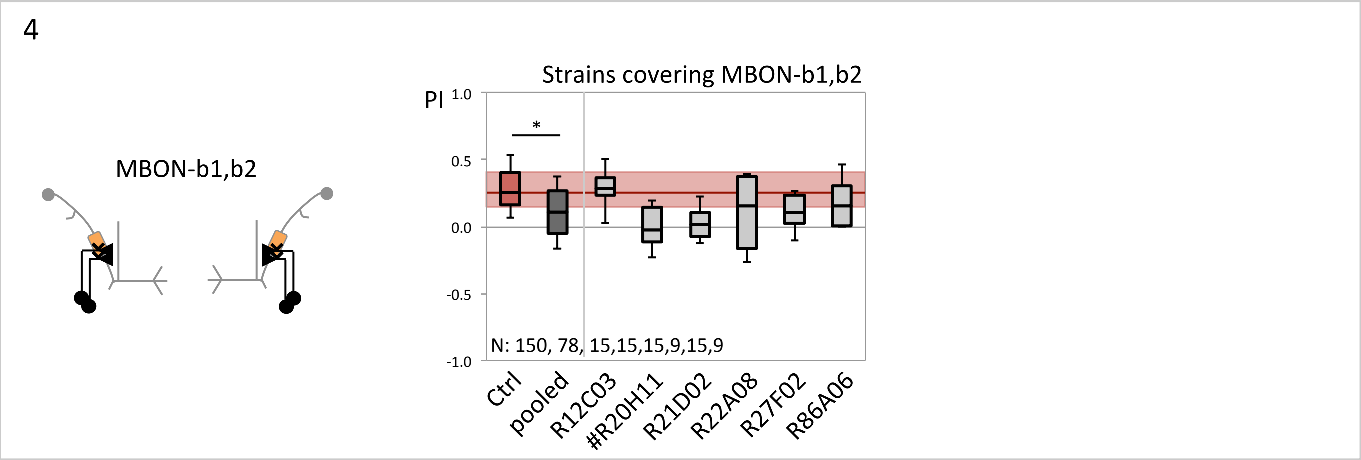

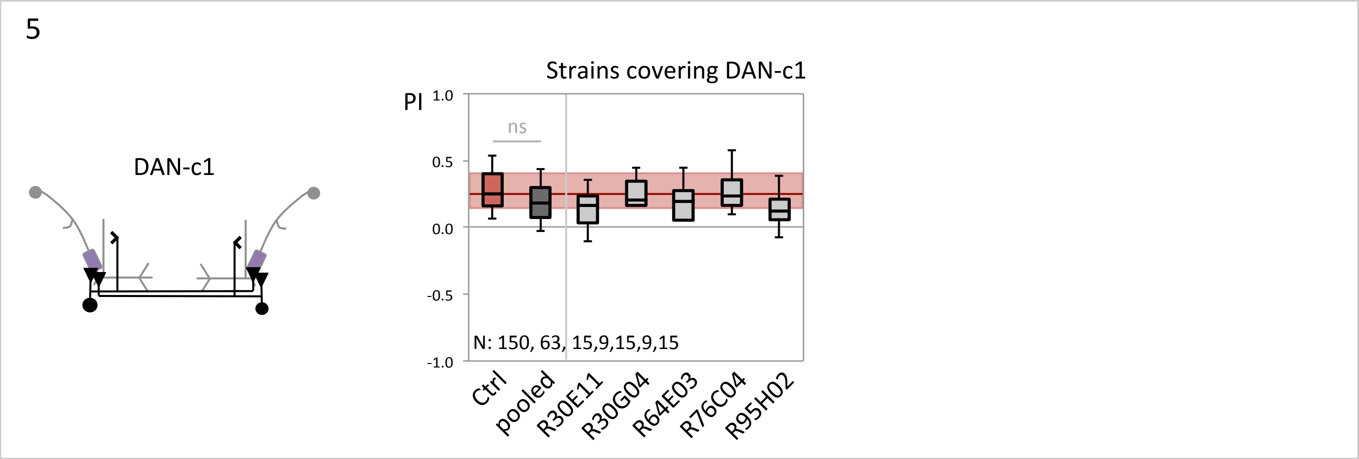

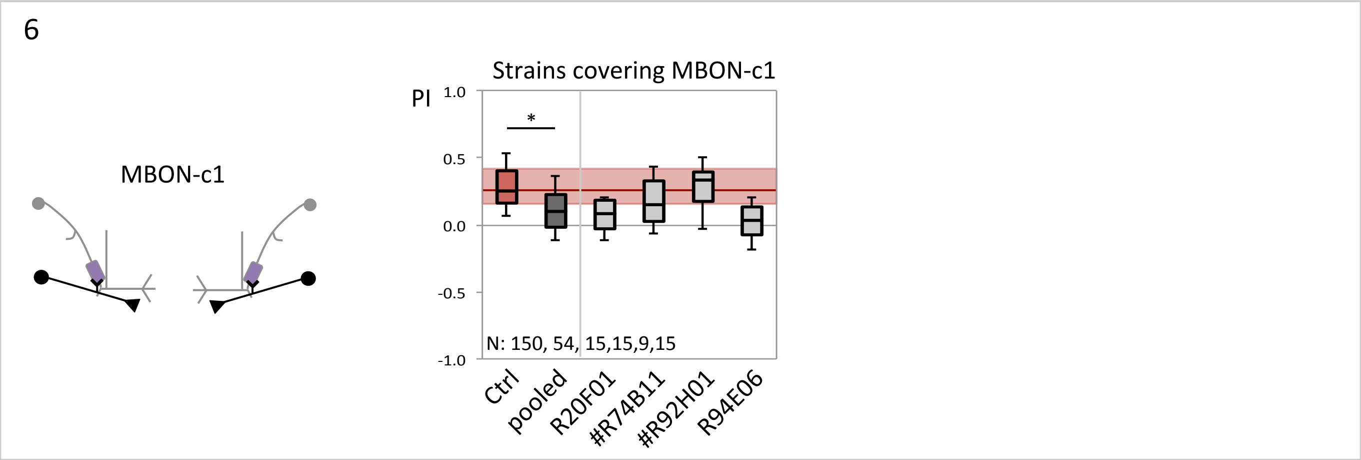

Ctd. Supplementary Figure 5

7

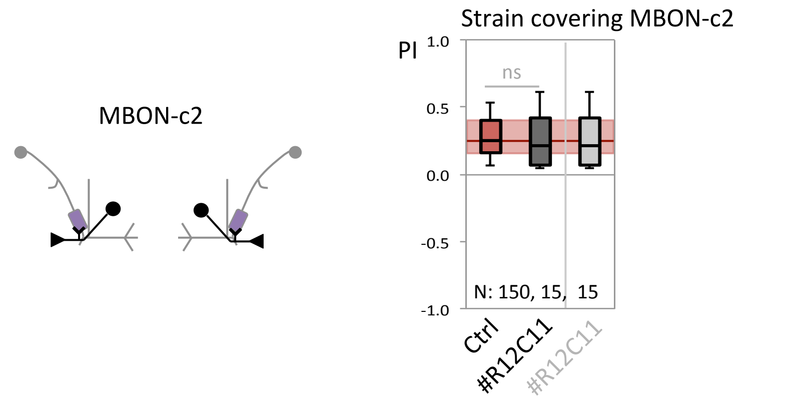

8

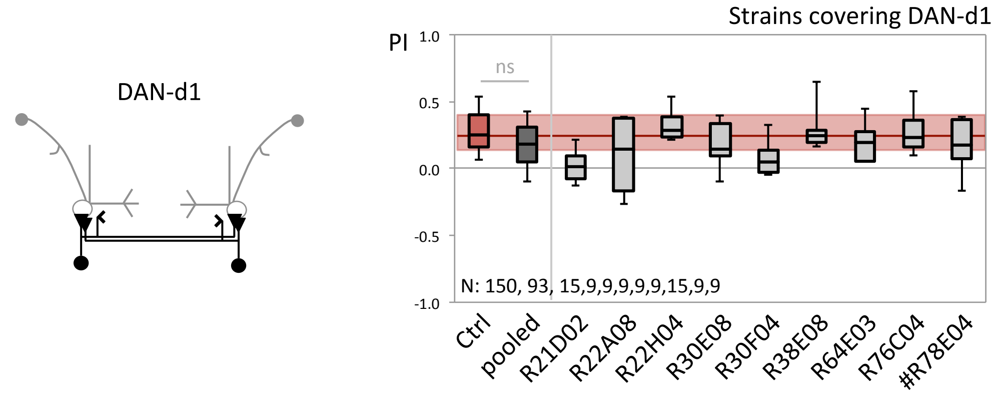

9

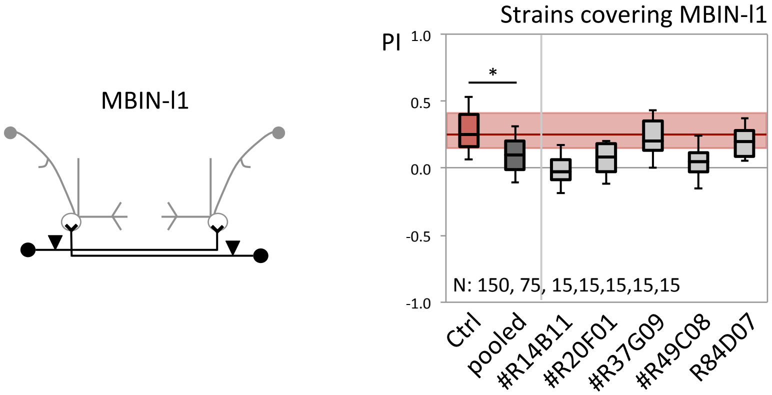

Ctd. Supplementary Figure 5

10

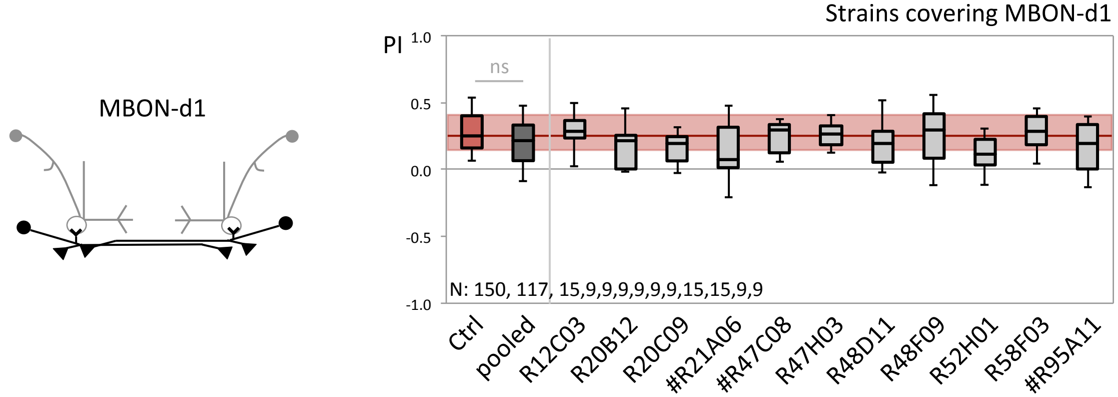

11

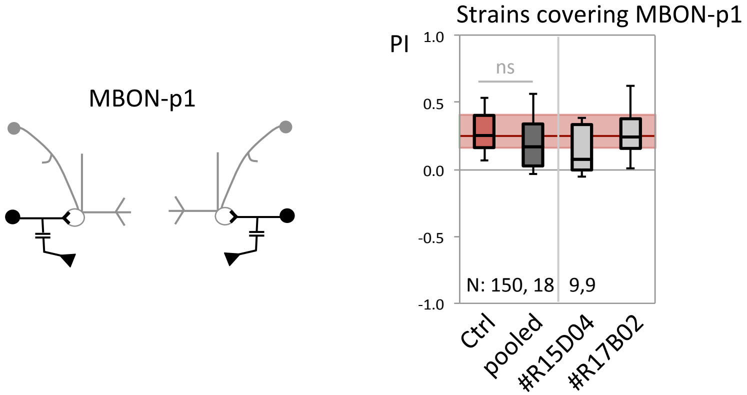

12

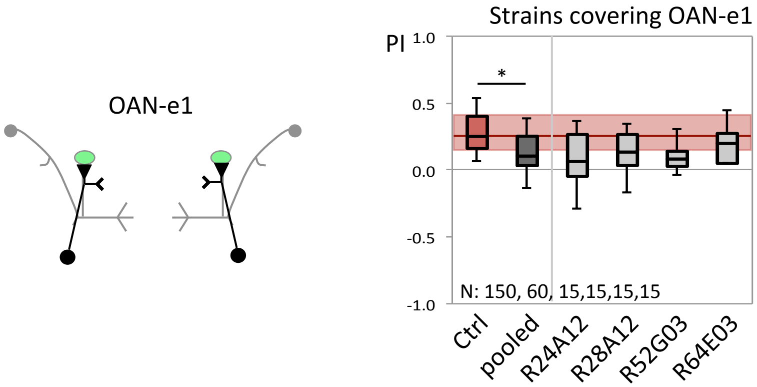

Ctd. Supplementary Figure 5

13

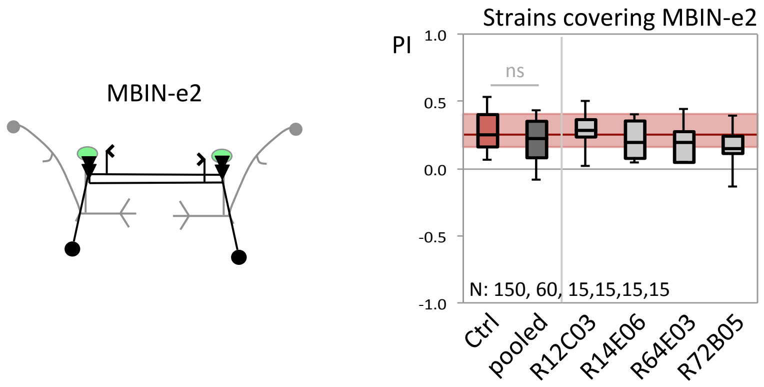

14

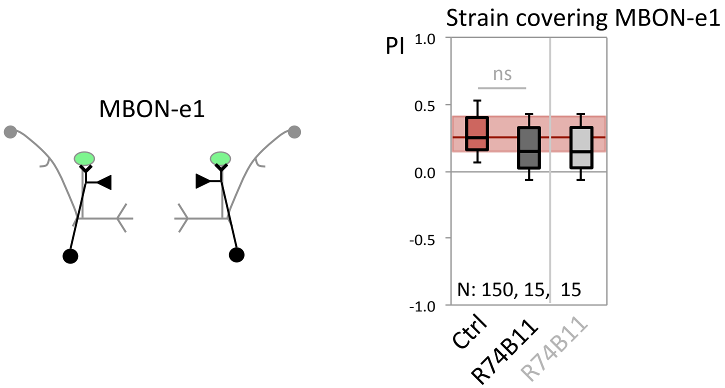

15

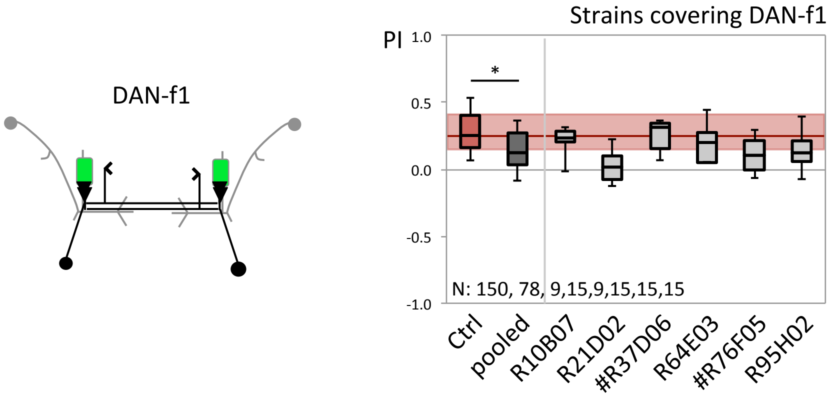

Ctd. Supplementary Figure 5

16

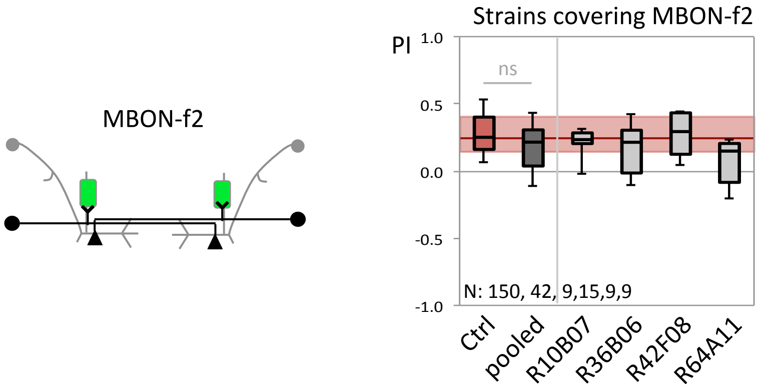

17

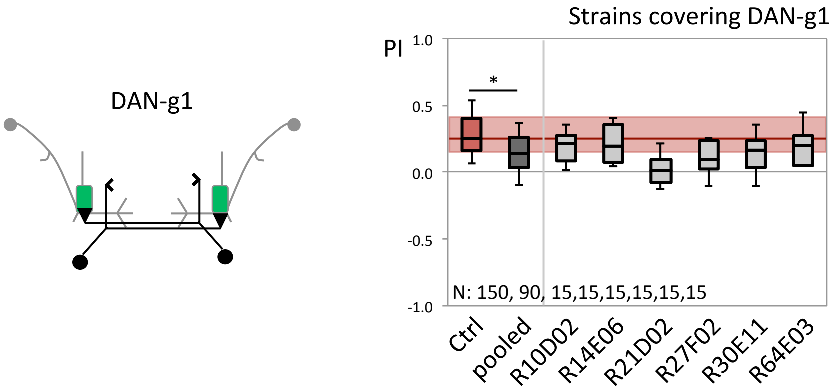

18

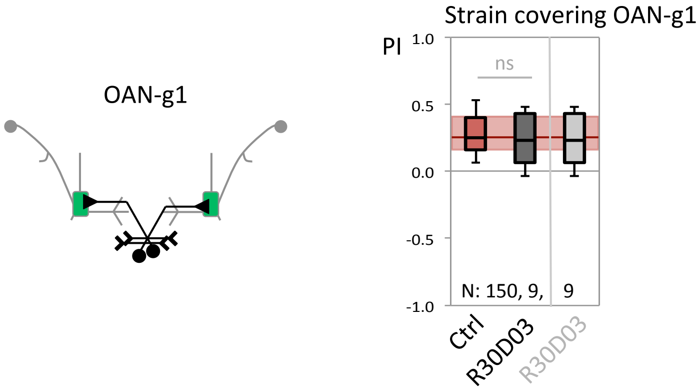

Ctd. Supplementary Figure 5

19

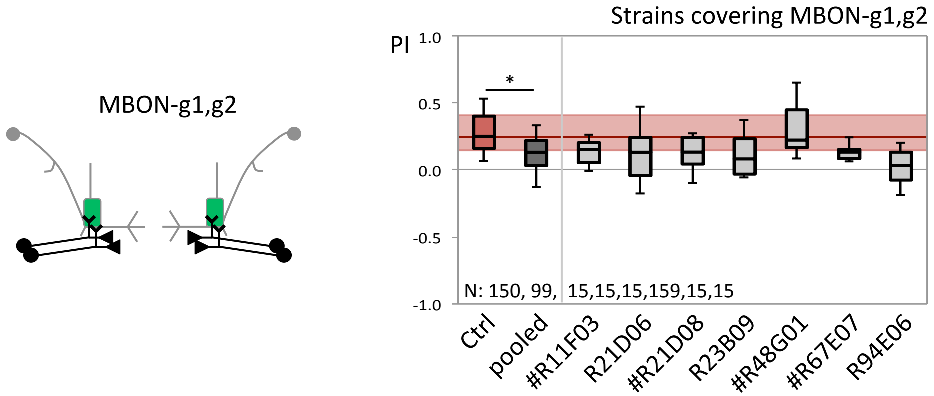

20

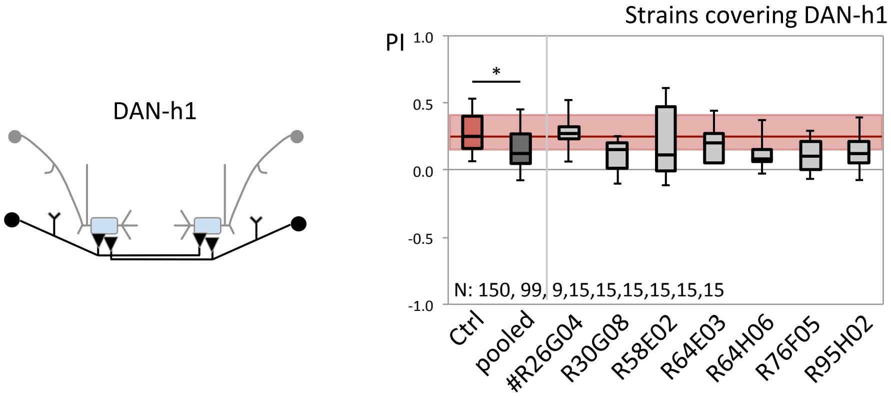

21

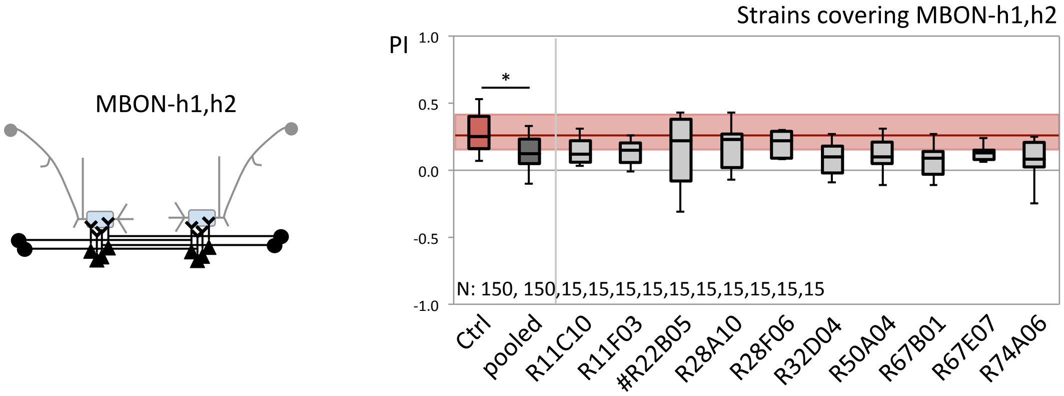

Ctd. Supplementary Figure 5

22

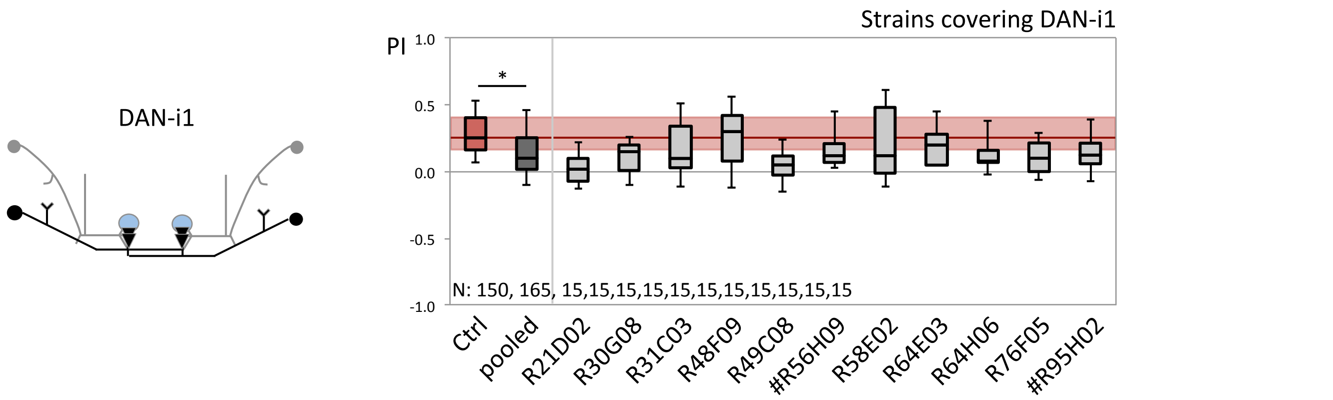

23

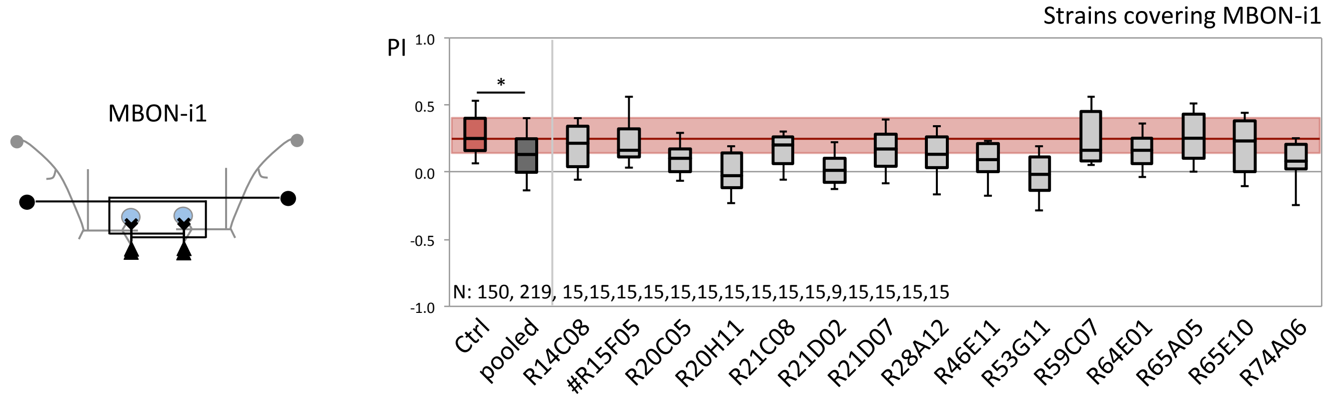

24

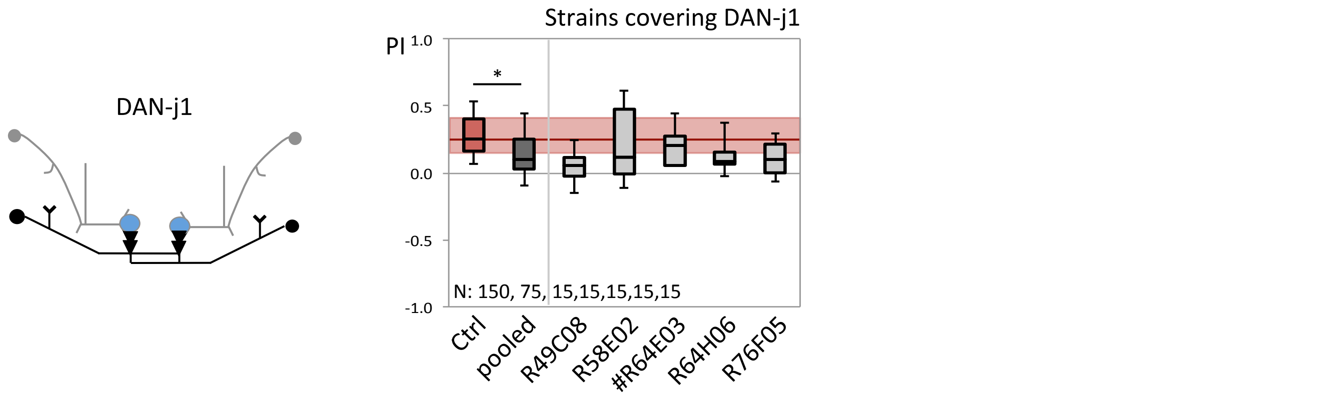

Ctd. Supplementary Figure 5

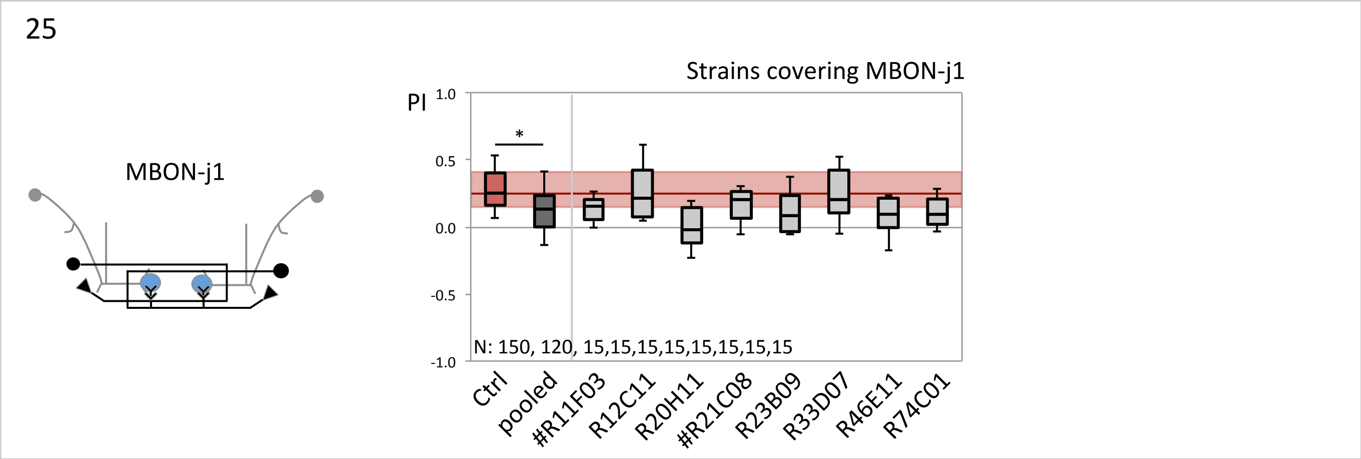

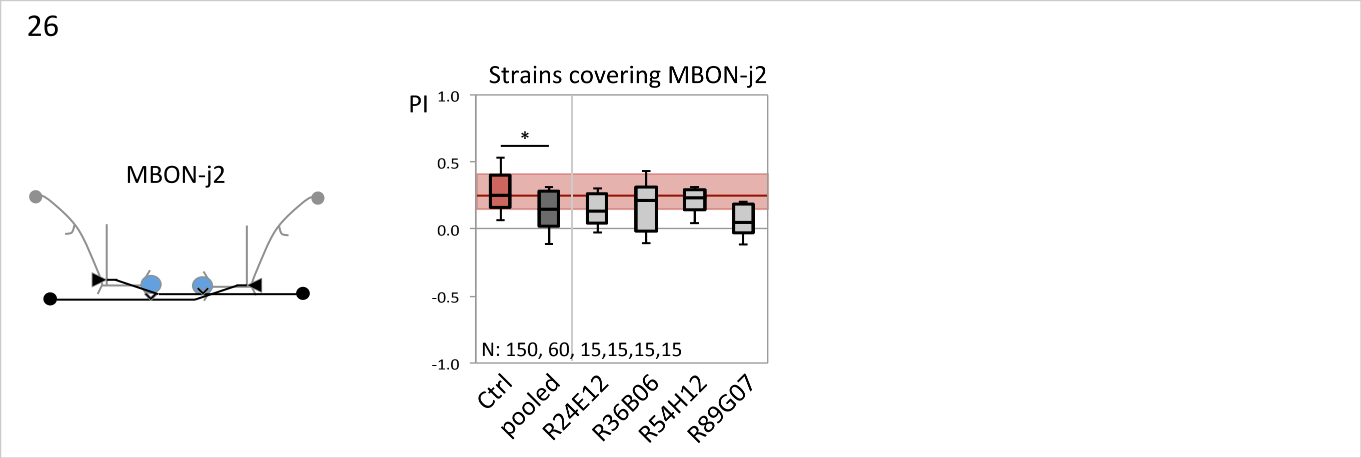

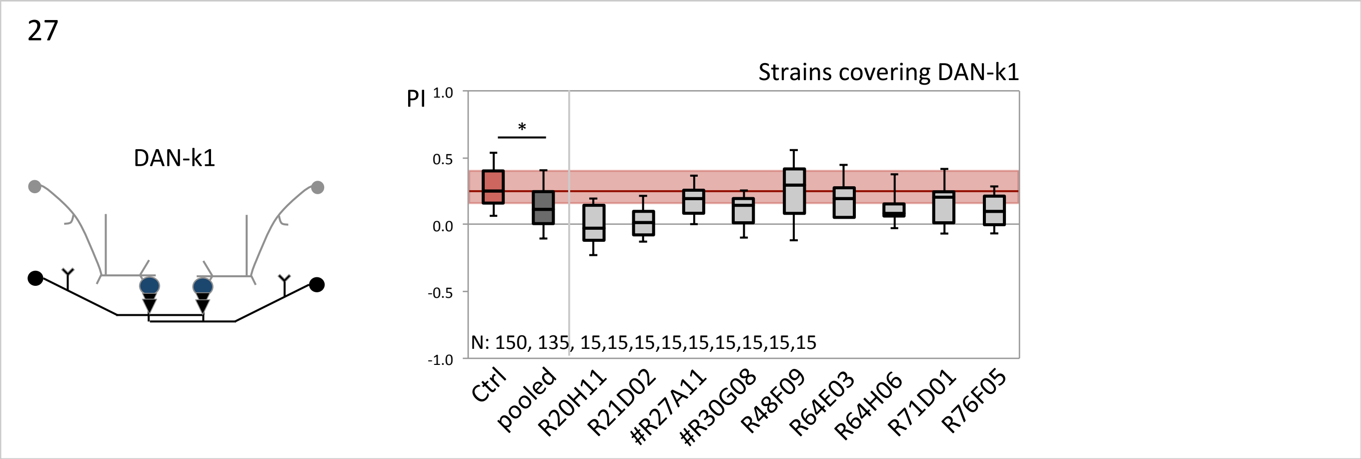

Ctd. Supplementary Figure 5

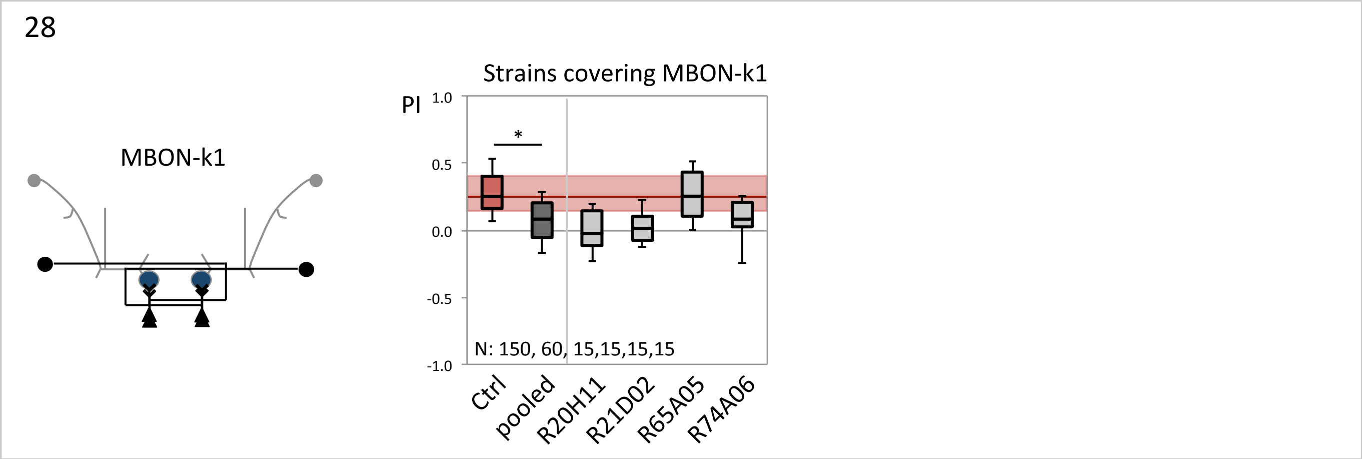

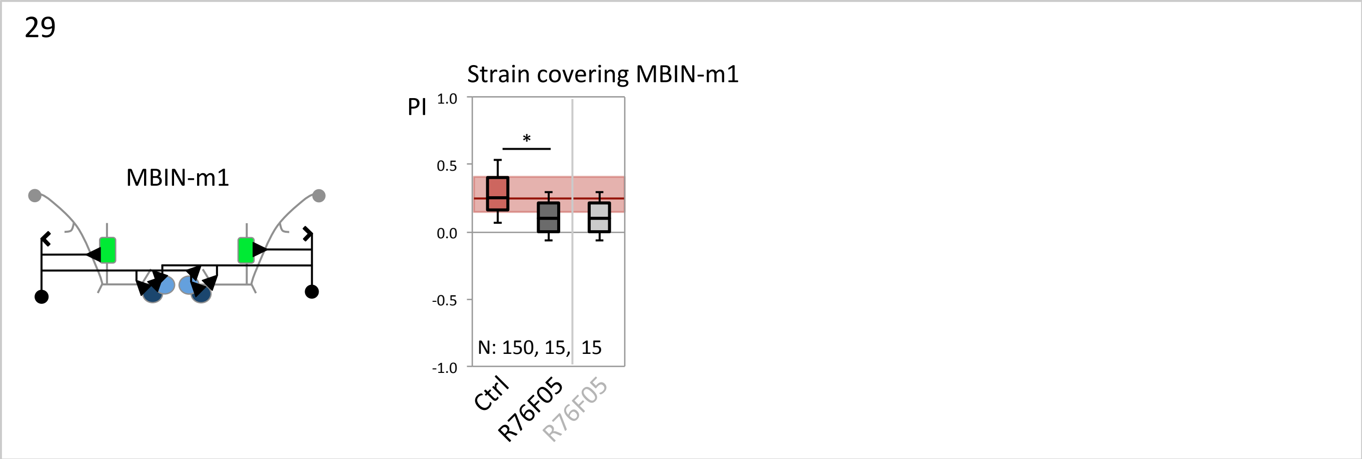

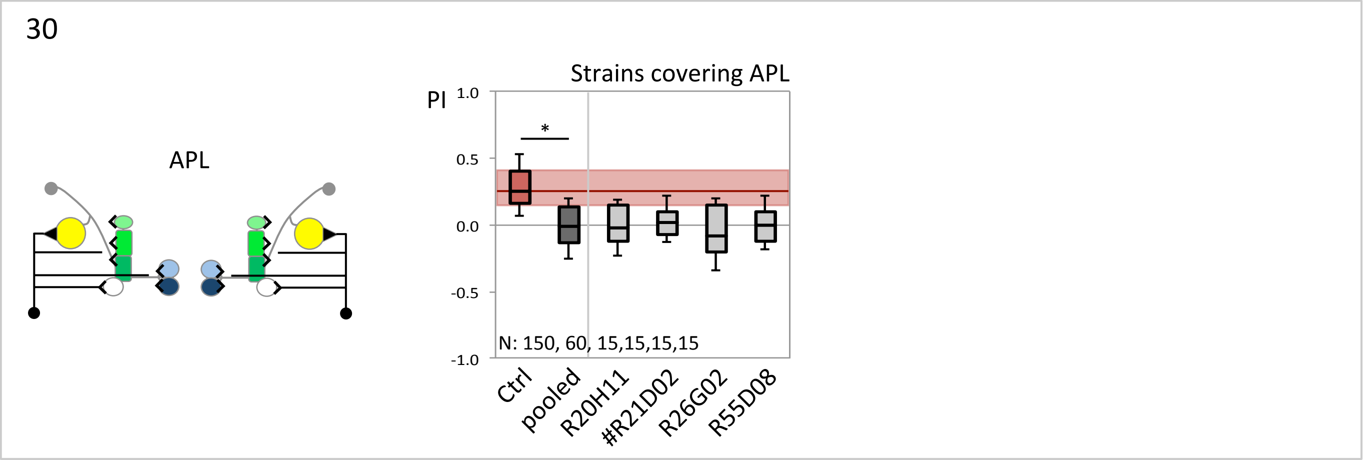

Ctd. Supplementary Figure 5

31

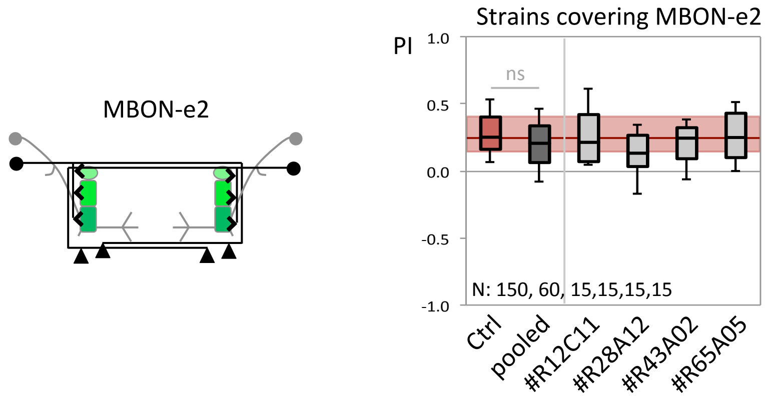

32

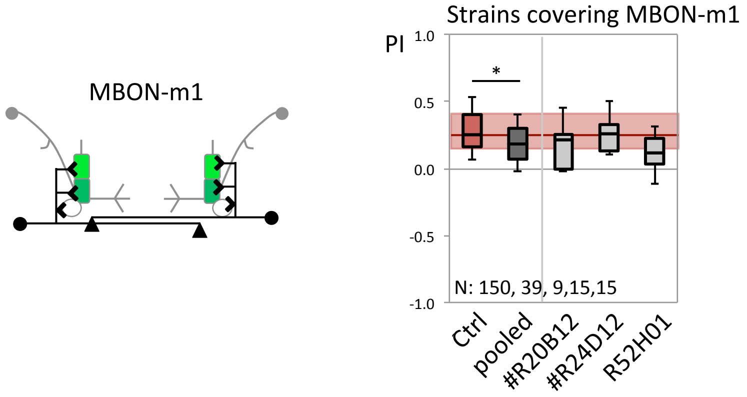

33

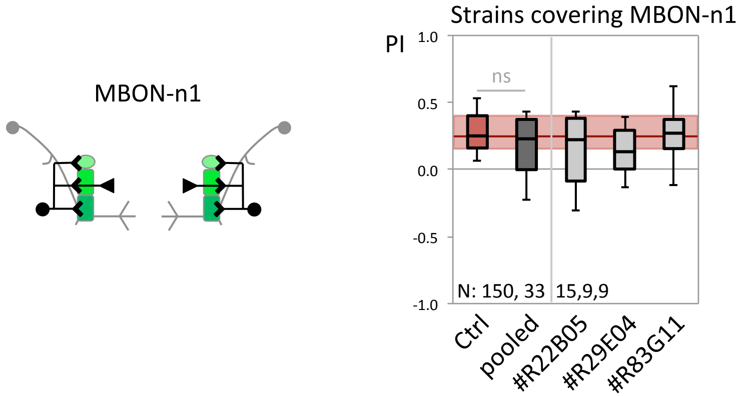

Ctd. Supplementary Figure 5

34

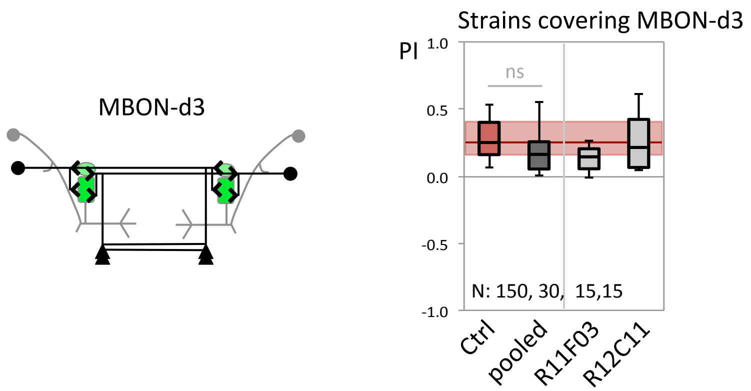

35

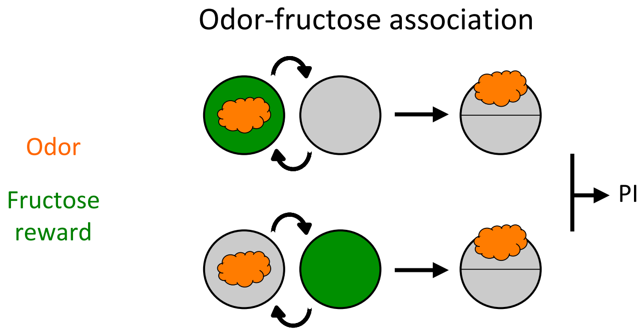

Supplementary Figure 5: Pre-screen results, combined for Gal4 strains covering the indicated MBE neuron

(Panels 1-34) Gal4 strains including MBE neurons in their expression pattern were crossed to *UAS-shi*^{ts}. At restrictive temperature and thus with synaptic output blocked from the respectively covered neurons, the larvae were trained in an odor-fructose reward associative learning task (panel 35). Appetitive odor-fructose memory is indicated by positive associative performance indices (PIs). Throughout panels 1-34, a schematic of the respectively indicated MBE neuron is shown in addition. The red shading indicates the 25/75 % quantile of the genetic control strain heterozygous for *UAS-shi*^{ts} but not expressing Gal4 (Ctrl). Sample sizes are indicated within the figure. * and ns refer to P< 0.05 and P> 0.05, respectively, in Mann-Whitney U-tests between the control and the data combined across the experimental strains presented individually to the right. The # symbol indicates that the strain yielded the respective cell as a flp-out clone, but that the cell cannot be discerned directly from the expression pattern of the Gal4 strain; this suggests stochastic and/or weak transgene expression in the respective cell and strain. In general, possible differences between Gal4 strains in stochasticity and level of transgene expression should be borne in mind when interpreting the pre-screen results. Also, there may be combinatorial effects when a Gal4 strain expresses in more than one MBE (Table 2, Table 3) and/or in neurons outside the mushroom body (2) (http://flweb.janelia.org/cgi-bin/flew.cgi). Further, many Gal4 strains used in the pre-screen led to transgene expression in more than one MBE so it was practically impossible to run all strains covering the same MBE in parallel. Last but not least, firm conclusions would require the full set of genetic and behavioral controls as employed in the follow-up experiments shown in Figure 3, Figure 4-6, Figure 8 and Figure 10.

(Panel 35) Schematic of the odor-fructose reward association paradigm; details as in Figure 3e.

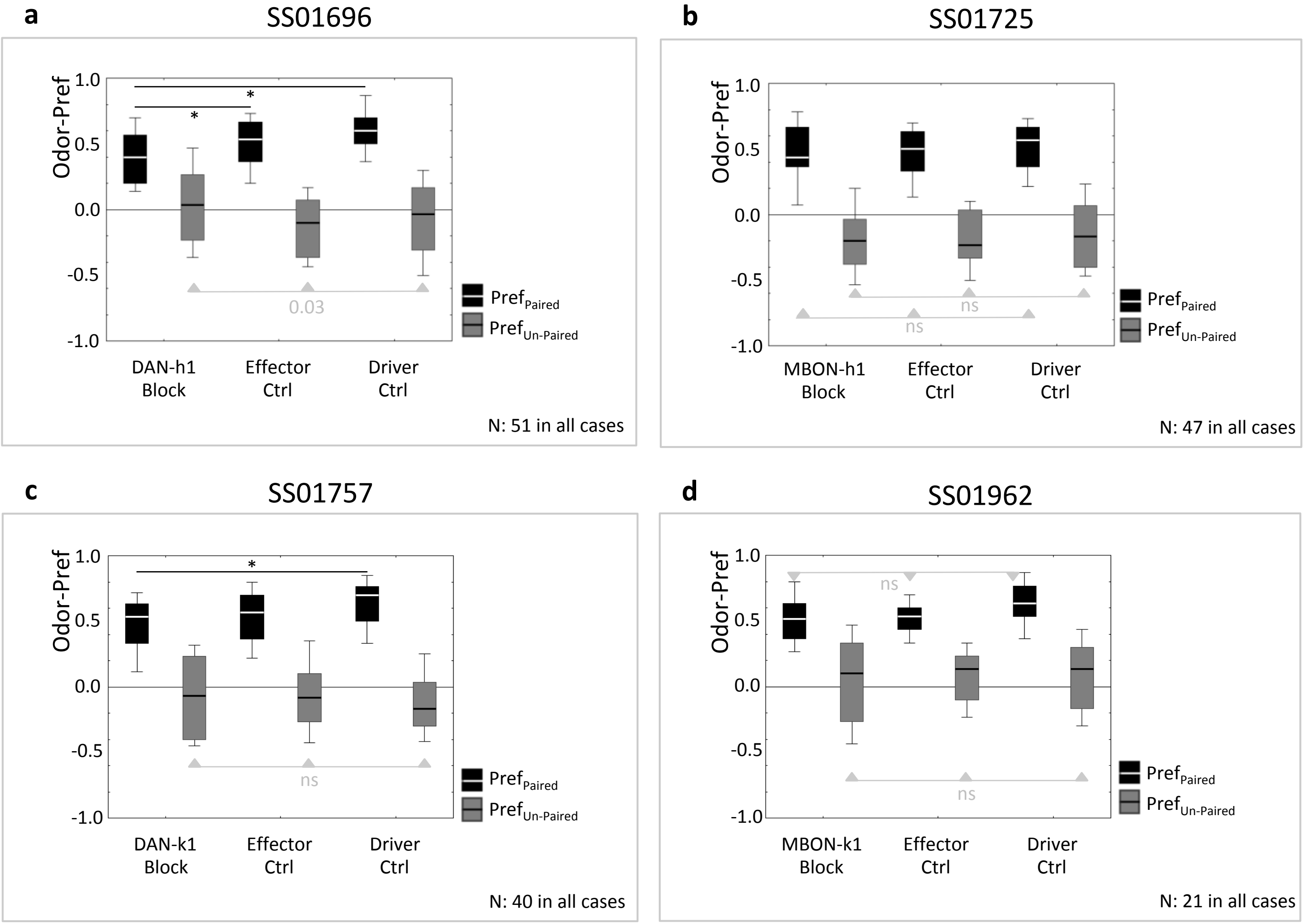

Supplementary Figure 6, ctd.

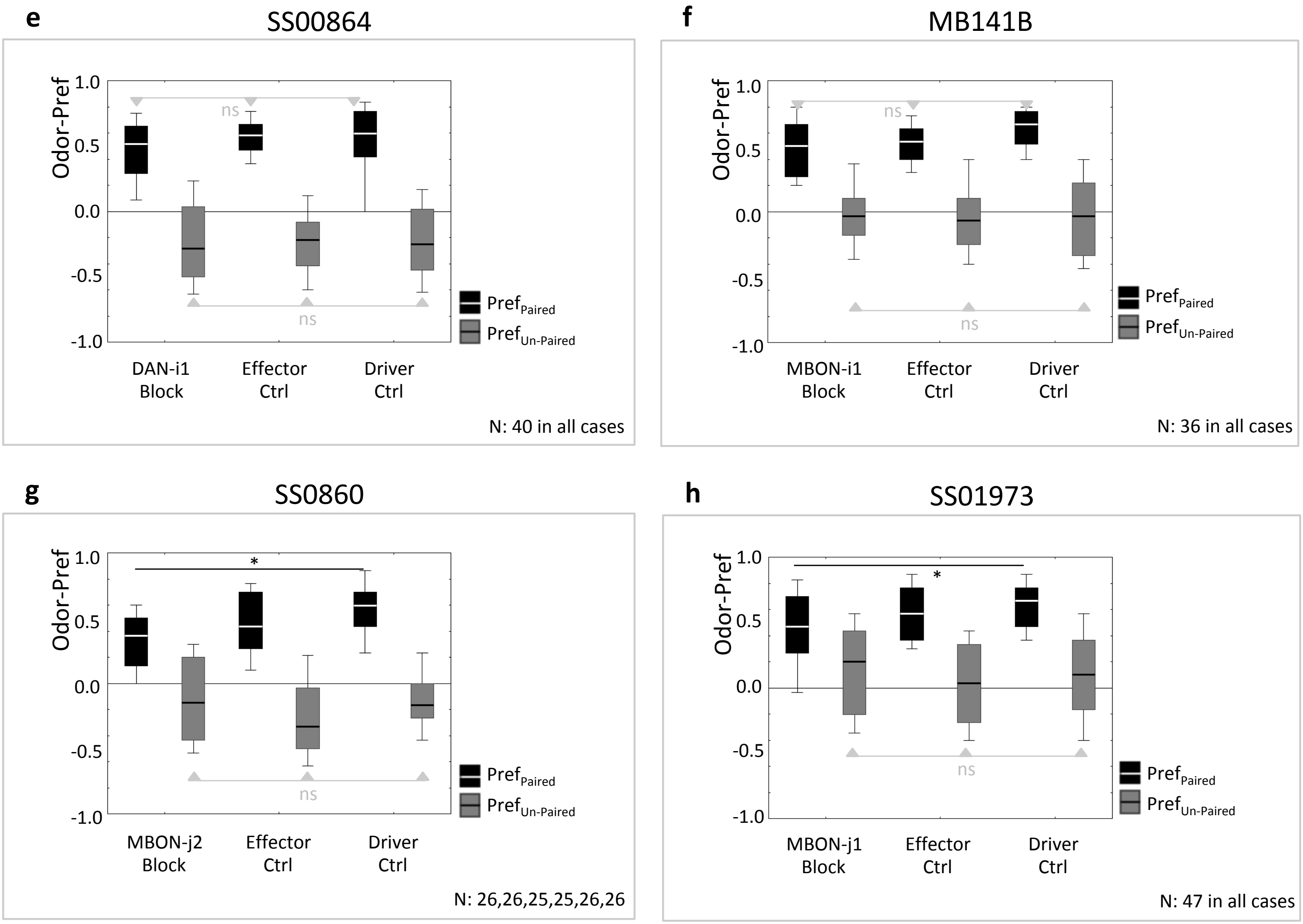

Ctd. Supplementary Figure 6

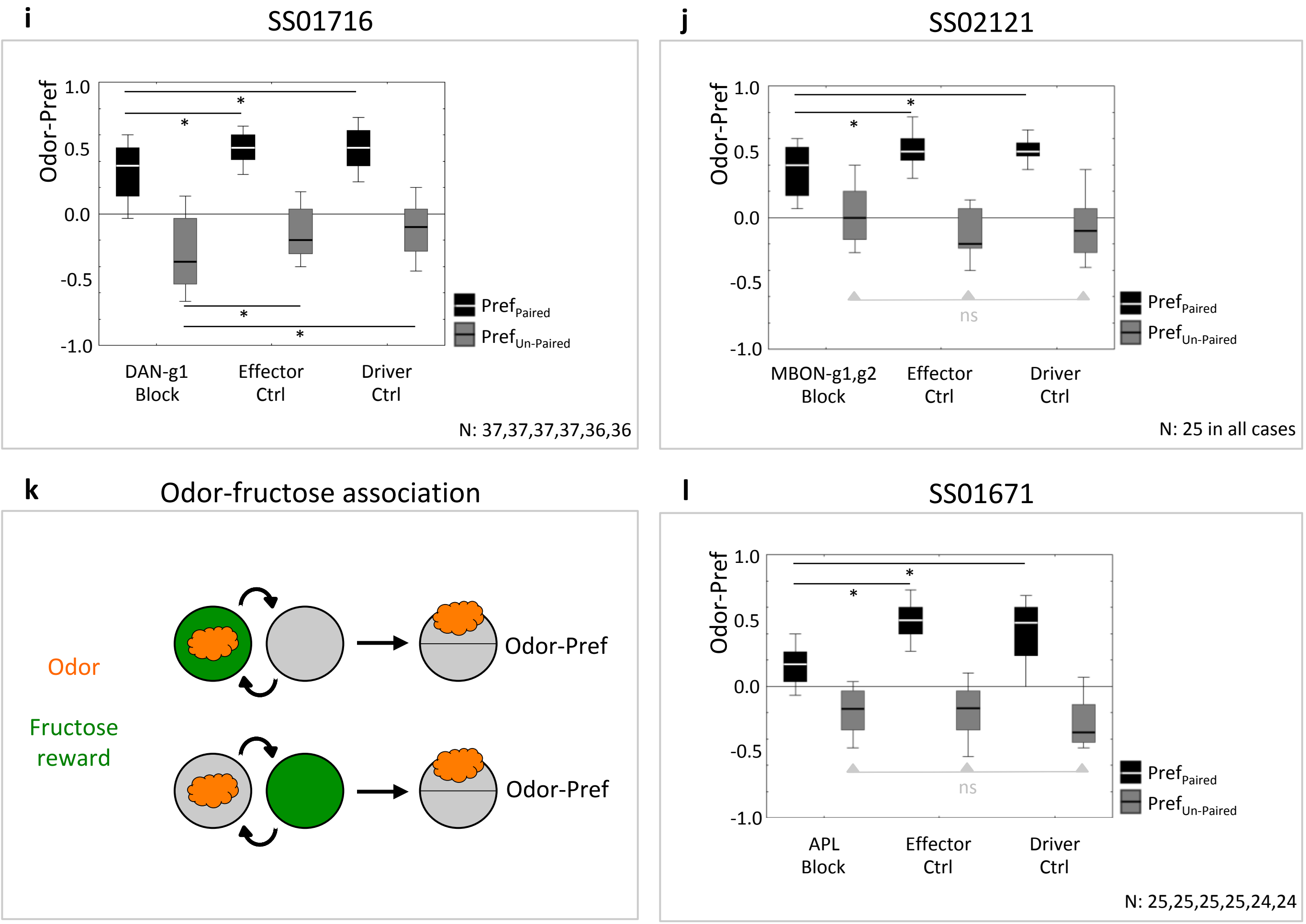

Supplementary Figure 6: Preference scores from odor-fructose association experiments upon silencing MBEs

(a-k) Preference scores for the trained odor *n*-amylacetate (Odor-Pref) underlying the associative performance indices (PI) displayed in Figure 3a-d and Figure 4a-f (a-j). A schematic of the training paradigm is shown in (k) (details as in Figure 3e).

(l) Same as in (a-j), regarding the associative performance indices (PI) displayed in Figure 10a.

Sample sizes are indicated within the figure. * refers to P< 0.05/2 in Mann-Whitney U-tests; ns refers to P> 0.05 in Kruskal-Wallis tests.

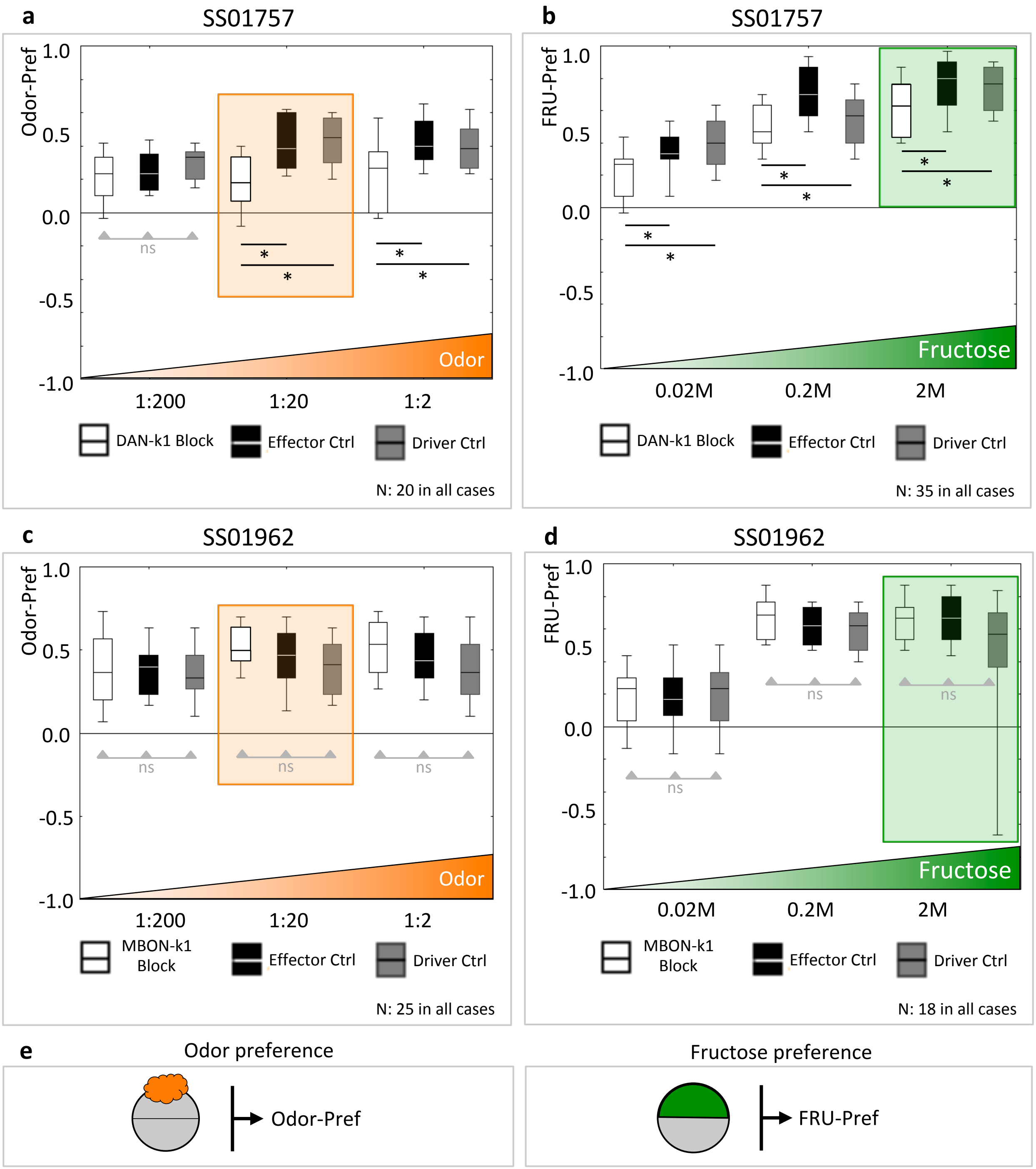

Supplementary Figure 7: Differential requirement of DAN-k1 but not MBON-k1 for innate odor- and fructose preference - extended

(a, b) For the split-Gal4 strain covering the MBIN to the lower toe of the medial lobe (DAN-k1; SS01757), the panels show the preference scores of experimentally naïve larvae for the odor *n*-amylacetate (a; AM), or for fructose (b; FRU), at the indicated dilutions/ concentrations. Larvae are either heterozygous for the split-Gal4 drivers as well as *UAS-Kir2.1::GFP* (DAN-k1 Block), or heterozygous for only the *UAS-Kir2.1::GFP* effector (Effector Ctrl), or heterozygous for only the split-Gal4 drivers (Driver Ctrl).

(c, d) Same as for (a, b), concerning the MBON of the same compartment (MBON-k1; SS01962).

(e) Schematics of the preference paradigms (details as in Figure 3e).

Please note that the data for *n*-amylacetate preference and fructose preference emphasized by the bounding boxes are also presented in Figure 3c for DAN-k1.

Sample sizes are indicated within the figure. * refers to P< 0.05/2 in Mann-Whitney U-tests; ns refers to P> 0.05 in Kruskal-Wallis tests.

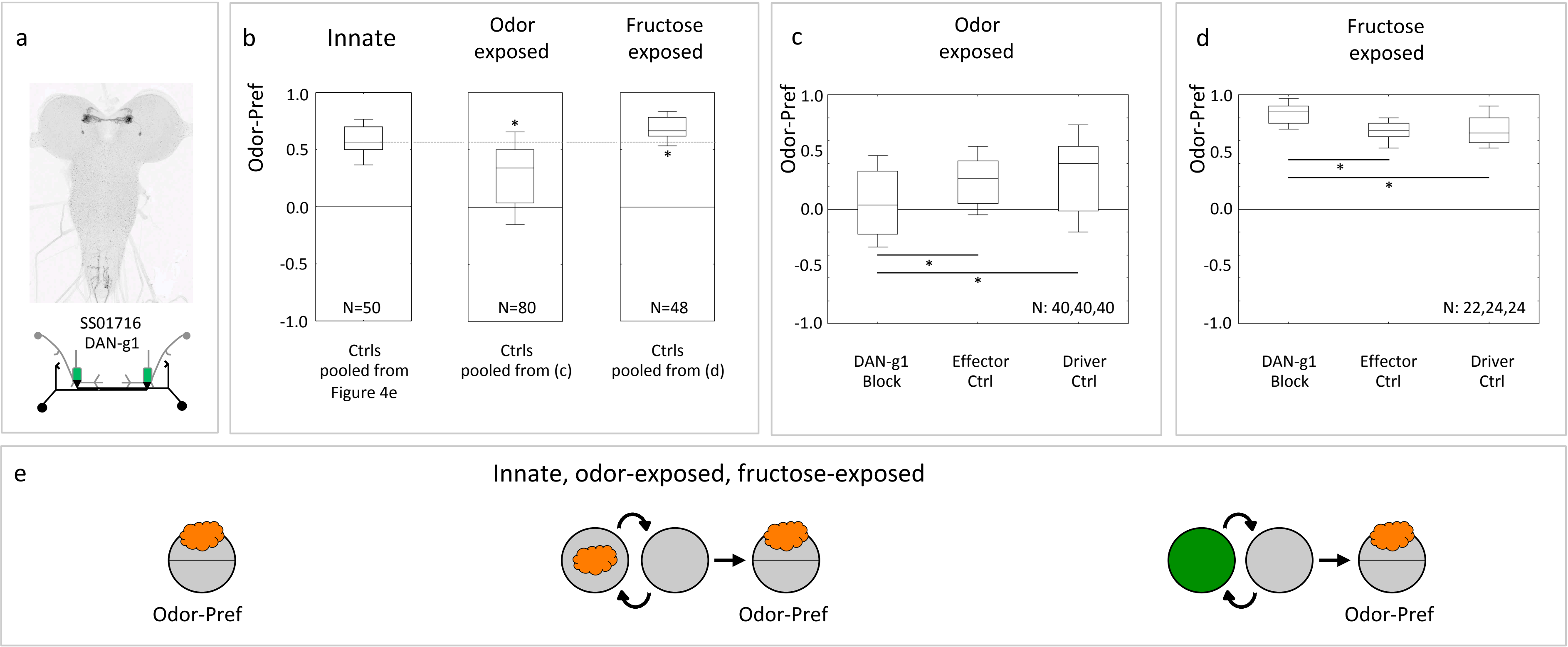

Supplementary Figure 8: Silencing DAN-g1 makes larvae more susceptible to the non-associative effects of stimulus-exposure

(a) Expression pattern of the split-Gal4 strain covering the MBON of the lower vertical lobe (DAN-g1; SS01716). Regarding this neuron, a closer inspection of the odor-fructose association data upon its silencing suggests an unexpected function. Although innate odor preference remains intact (Figure 4e), odor preference scores are shifted towards the negative both after paired odor-fructose reward training and after unpaired presentations of odor and fructose (Figure S6i). In analyses of associative learning, such effects are intentionally averaged-out by calculating the difference in preference between paired versus unpaired-trained groups for the associative performance index (Figure 3e). Importantly, no such general, non-associative shift in preference towards the negative is seen for any other neuron in this study (Figure S6); in particular, it is not seen for the output neuron double pair of the compartment innervated by DAN-g1 (MBON-g1,g2; Figure S6j). It was thus asked whether silencing DAN-g1 specifically affects odor preference after non-associative stimulus exposure.

(b-d) In genetic controls odor exposure decreases odor preference (b; 3-4), an effect that is boosted upon silencing DAN-g1 (c). Fructose exposure increases odor preference in genetic controls (b; 3-4), an effect that is likewise boosted by silencing DAN-g1 (d). Thus, silencing DAN-g1 makes the larvae more susceptible to the effects of odor-only exposure and to the effects of fructose-only exposure; undisturbed activity in DAN-g1 thus appears to be required to partially 'buffer' olfactory behavior against non-associative effects of stimulus-exposure. Sample sizes are indicated within the figure. In (b) * refers to $P < 0.05/2$ in Mann-Whitney U-tests versus innate odor preference. In (c, d) * refers to $P < 0.05/2$ in Mann-Whitney U-tests.

(e) Schematic of the paradigms to measure innate odor preference in experimentally naïve larvae, odor preference after odor exposure, and odor preference after fructose exposure (details as in Figure 3e).

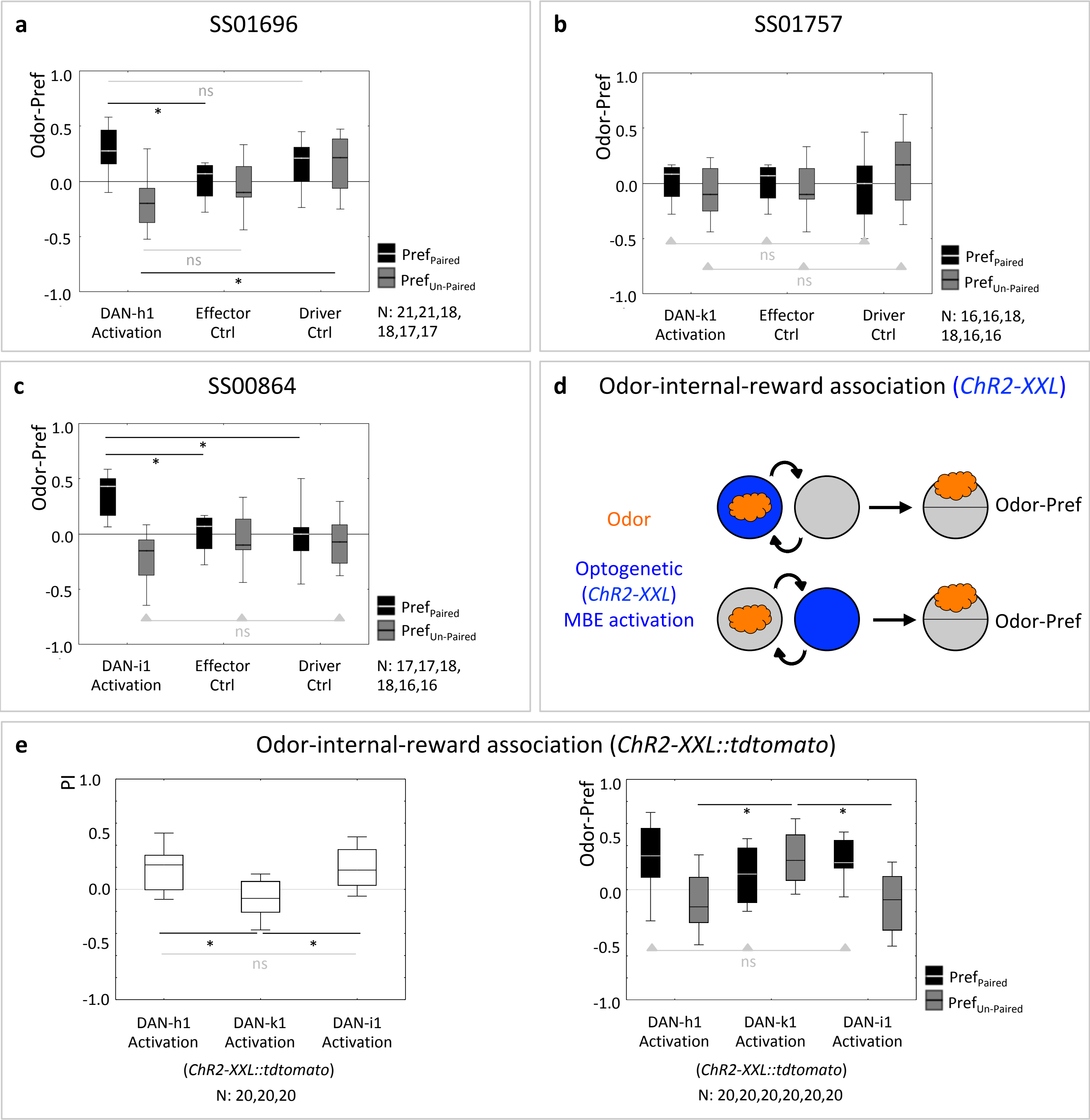

Supplementary Figure 9: Preference scores from odor-internal-reward association experiments using ChR2-XXL, and use of ChR2-XXL::tdtomato for odor-internal-reward association experiments

(a-c) Preference scores for the trained odor *n*-amylacetate (Odor-Pref) underlying the associative performance indices (PI) displayed in Figure 5a-c using *ChR2-XXL* for optogenetic activation of the indicated DAN as an internal reward.

(d) Schematic of the training paradigm (details as in Figure 5d).

(e) Performance indices and Preference scores for odor-internal-reward association using *ChR2-XXL::tdtomato* as the effector. As with *ChR2-XXL*, DAN-h1 and DAN-i1, but not DAN-k1, are sufficient as an internal reward signal.

Sample sizes are indicated within the figure. At plain horizontal lines * refers to P< 0.05/2 and ns to P> 0.05/2 in Mann-Whitney U-tests; at horizontal lines with arrowheads, ns refers to P> 0.05 in Kruskal-Wallis tests.

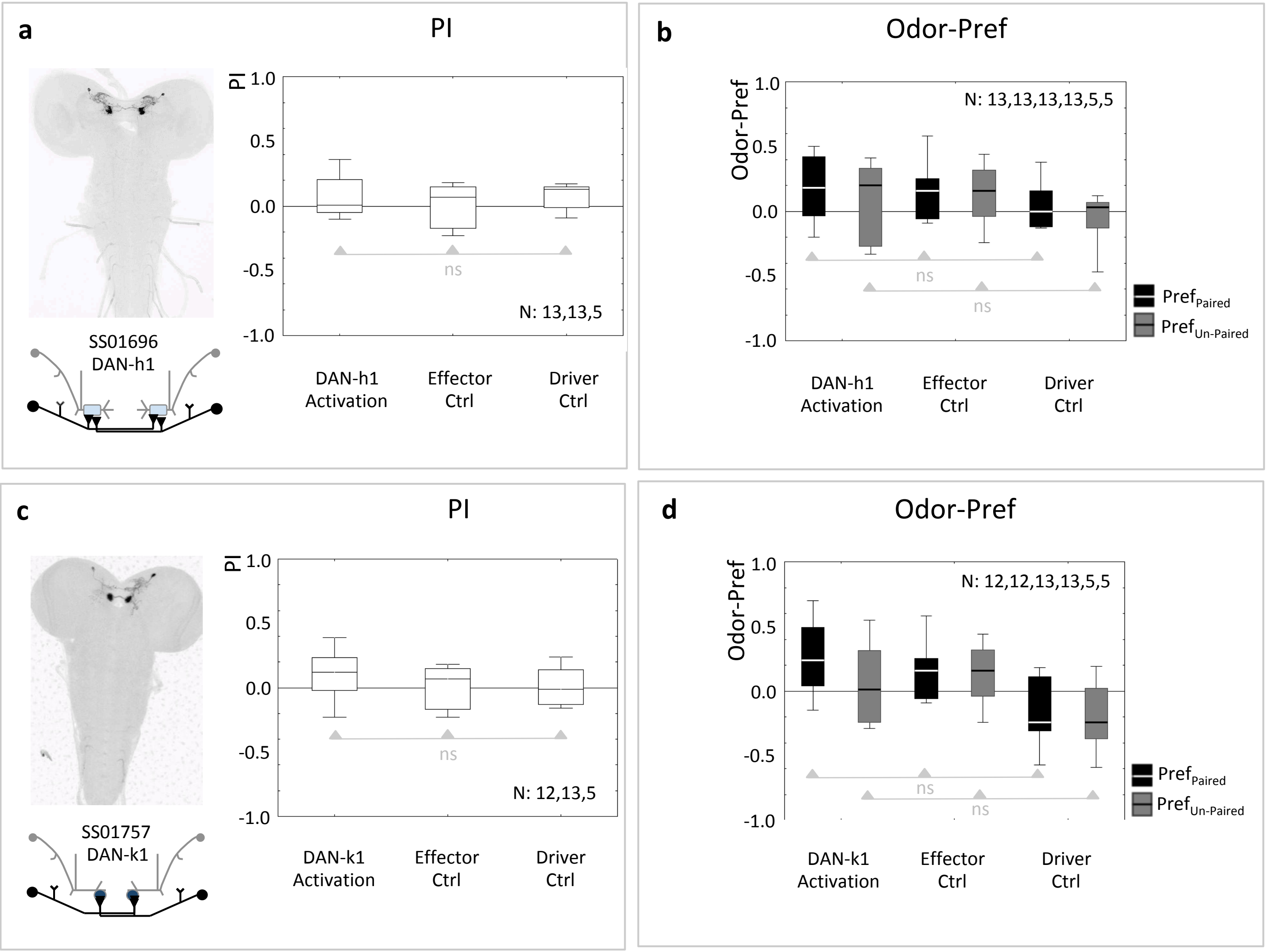

Saumweber et al. Fig S10, ctd

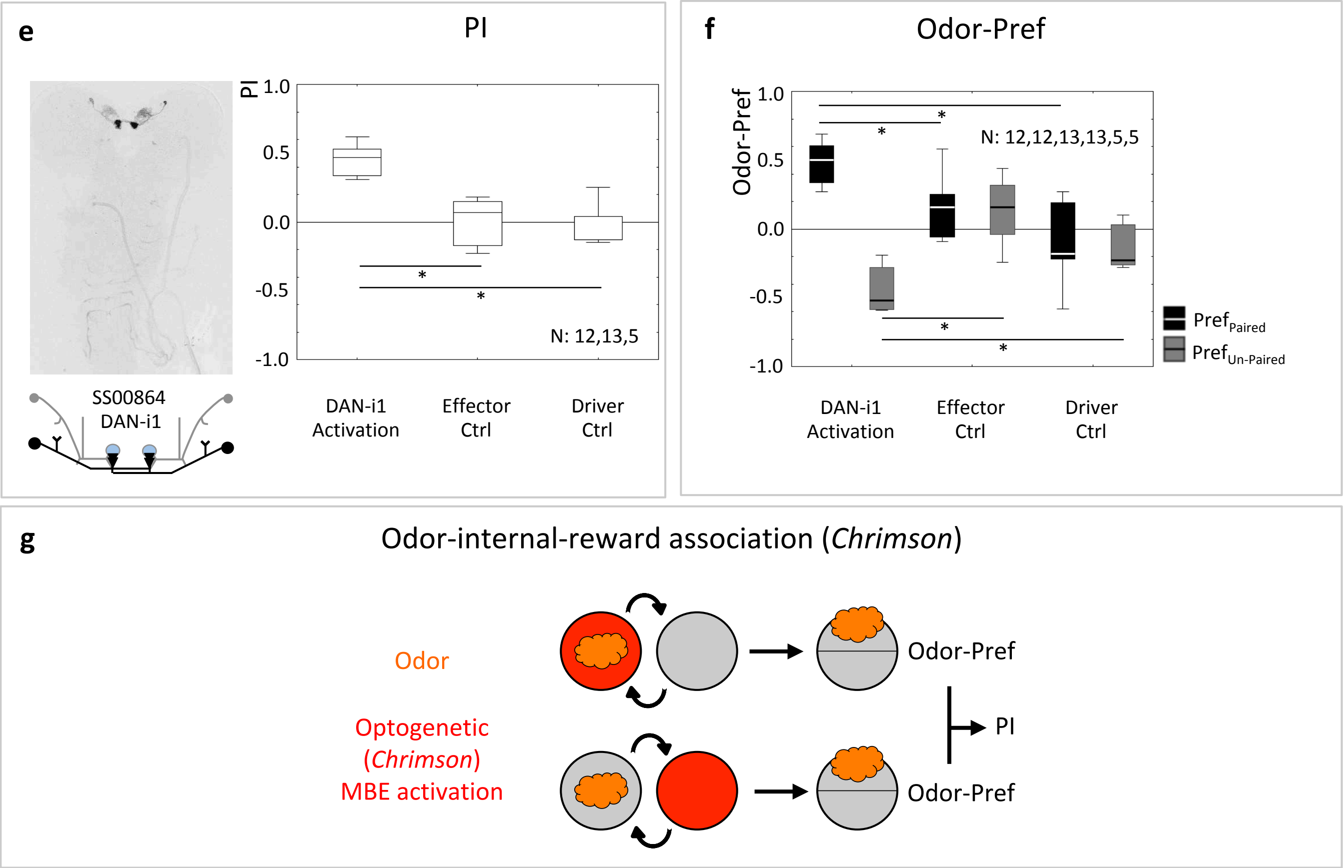

Supplementary Figure 10: Odor-internal-reward association experiments using Chrimson

(a-f) Larvae are trained with odor stimulation and optogenetic activation of the indicated DANs as an internal reward signal. The panels show the expression pattern of the indicated split-Gal4 strain and a schematic overview of the covered DAN, as well as the associative performance indices after internal-reward training (a, c, e), and the underlying preference scores (b, d, f). Larvae are either heterozygous for the split-Gal4 drivers as well as *UAS-Chrimson* (DAN-Activation), or heterozygous for only the *UAS-Chrimson* effector (Effector Ctrl), or heterozygous for only the split-Gal4 drivers (Driver Ctrl). Activation of DAN-h1 (a, b) as well as activation of DAN-k1 (c, d) is without measurable effect, whereas activation of DAN-i1 (e, f) is sufficient as an internal reward.

(g) Schematic of the associative learning paradigm with odor and internal-reward (details as in Figure 5d, except that red light is used for the activation of neurons by Chrimson).

Sample sizes are indicated within the figure. * refers to P< 0.05/2 in Mann-Whitney U-tests; ns refers to P> 0.05 in Kruskal-Wallis tests.

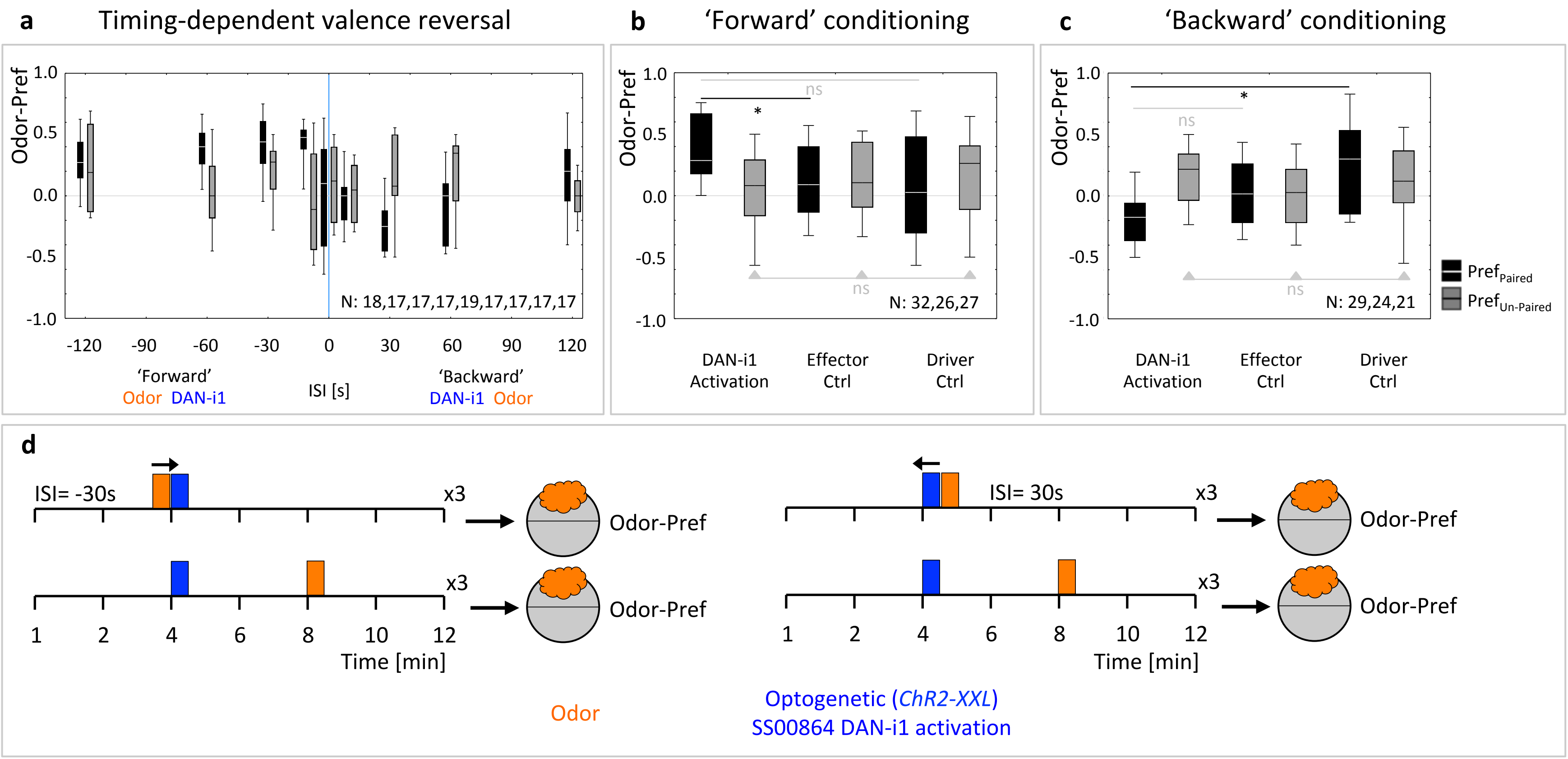

Supplementary Figure 11: Preference scores from timing-dependent valence reversal of optogenetic DAN-i1 reinforcement

(a-c) Preference scores for the trained odor *n*-amylacetate (Odor-Pref) underlying the associative performance indices (PI) displayed in Figure 6a-c using *ChR2-XXL* for optogenetic activation of DAN-i1 as an internal reward at various relative timings between odor presentation and DAN-i1 activation (Inter-Stimulus-Interval, ISI)

(d) Schematic of the training paradigm (details as in Figure 6d).

Sample sizes are indicated within the figure. At plain horizontal lines * refers to P< 0.05/2 and ns to P> 0.05/2 in Mann-Whitney U-tests; at horizontal lines with arrowheads, ns refers to P> 0.05 in Kruskal-Wallis tests.

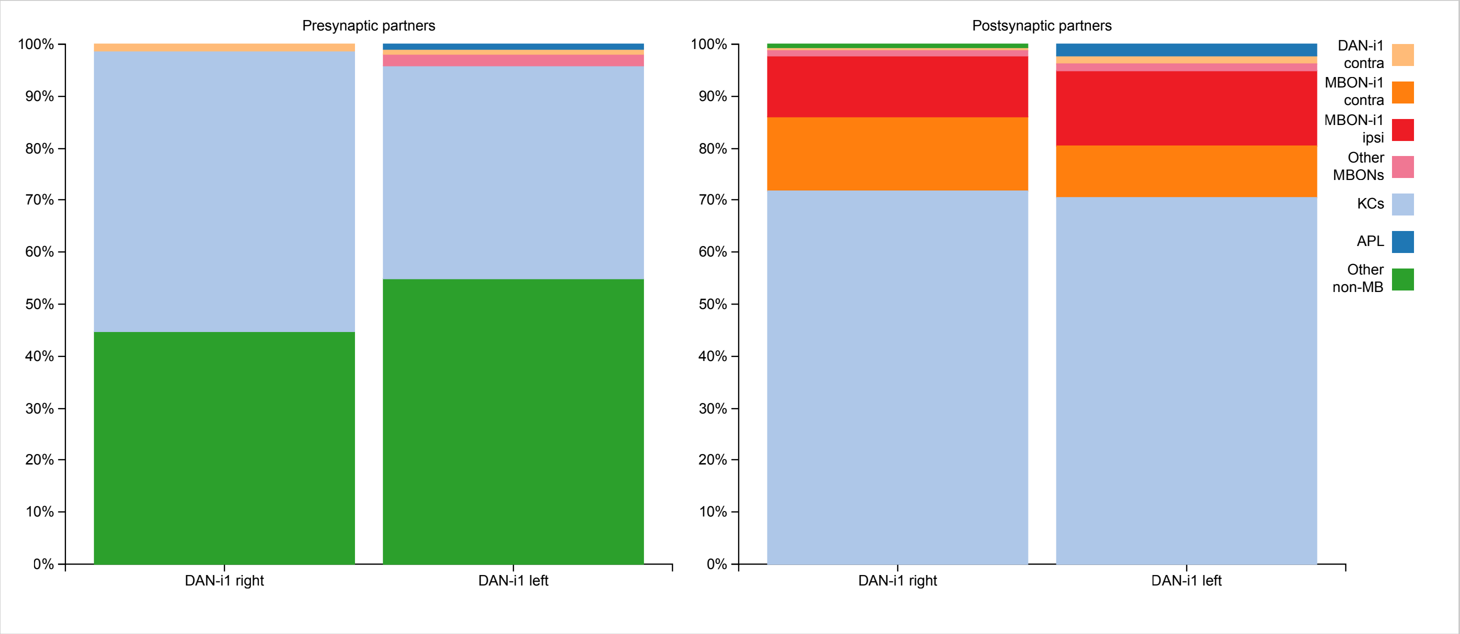

Supplementary Figure 12: Synaptic partners of DAN-i1

Number of presynaptic partners of DAN-i1 as a percentage of the total number of synaptic inputs to DAN-i1 (left panel), and number of postsynaptic partners of DAN-i1 as a percentage of the total number of synaptic outputs from DAN-i1 (right panel), in both cases separated for the right- and left-hemisphere DAN-i1. Thus, DAN-i1 collects input from outside the MB, and connects reciprocally to KCs; it also delivers about a third of its outputs to the homo-compartmental MBON-i1, about equally towards MBON-i1 of the right and left hemisphere. This corresponds to the ‘canonical circuit’ described in (1).

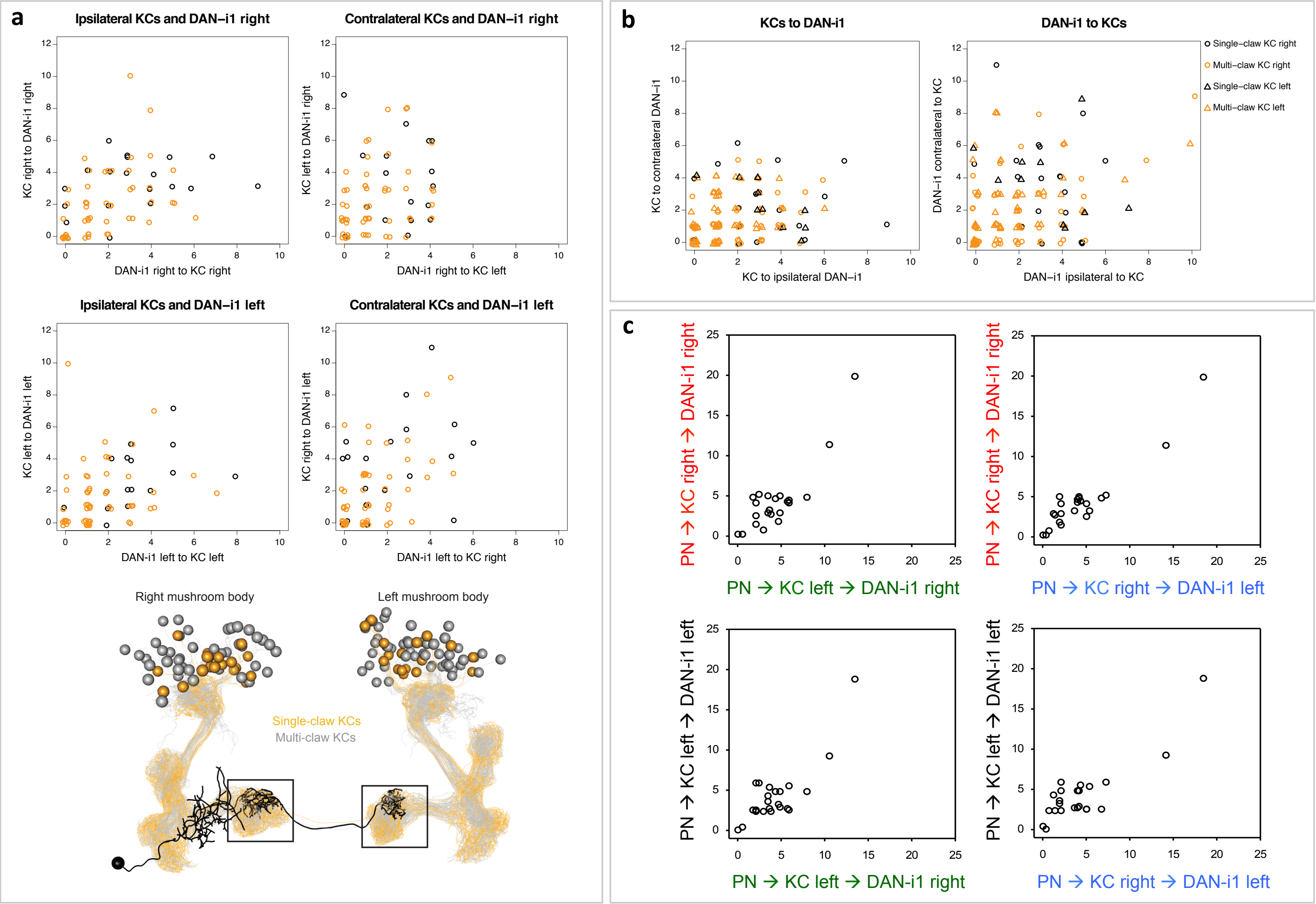

Supplementary Figure 13: Connectivity of DAN-i1 with KCs

(a) Data from Figure 7d, separated by body side. That is, for those KCs reciprocally connected with DAN-i1, the number of KC-to-DAN-i1 synapses versus the number of DAN-i1-to-KC synapses is plotted. The two top panels present these data for the right-hemisphere DAN-i1, separated for the KCs ipsi- and contralateral to it (left and right panel, respectively). The two middle panels show the same for the left-hemisphere DAN-i1. Single- and multi-claw KCs are presented separately. Apparent correlations (Spearman rank correlation analyses at P< 0.05) contrast with the case of the APL neuron where no such correlations are found (Figure 9c). For an overview, the bottom panel reproduces Figure 7a. (b) KC connections to the ipsi- and contralateral DAN-i1. For each KC the number of synapses to the ipsilateral DAN-i1 versus the number of its synapses to the contralateral DAN-i1 is plotted (left panel), as are the numbers of the corresponding DAN-i1-to-KC synapses (right panel). In both panels single- and multi-claw KCs are plotted separately. This reveals a mild yet significant correlation between the number of synapses by which KCs connect to the ipsi- and contralateral DAN-i1. The correlations were determined with Spearman rank correlation analyses at P< 0.05. (c) The correlations of the PN-KC-DAN-i1 matrix product reveal that both DAN-i1s sample the KC coding space in the same way, and that likewise each DAN-i1 samples the ipsi- and contralateral KC coding space in the same way, and in proportion to the fraction of PN-KC synapses that the respective PN class contributes (which is correlated between right and left hemisphere; not shown). The same correlations are found in the case of the APL neuron (Figure 9d; Supplementary Fig. 17a). The correlations were determined with Spearman rank correlation analyses at P< 0.05.

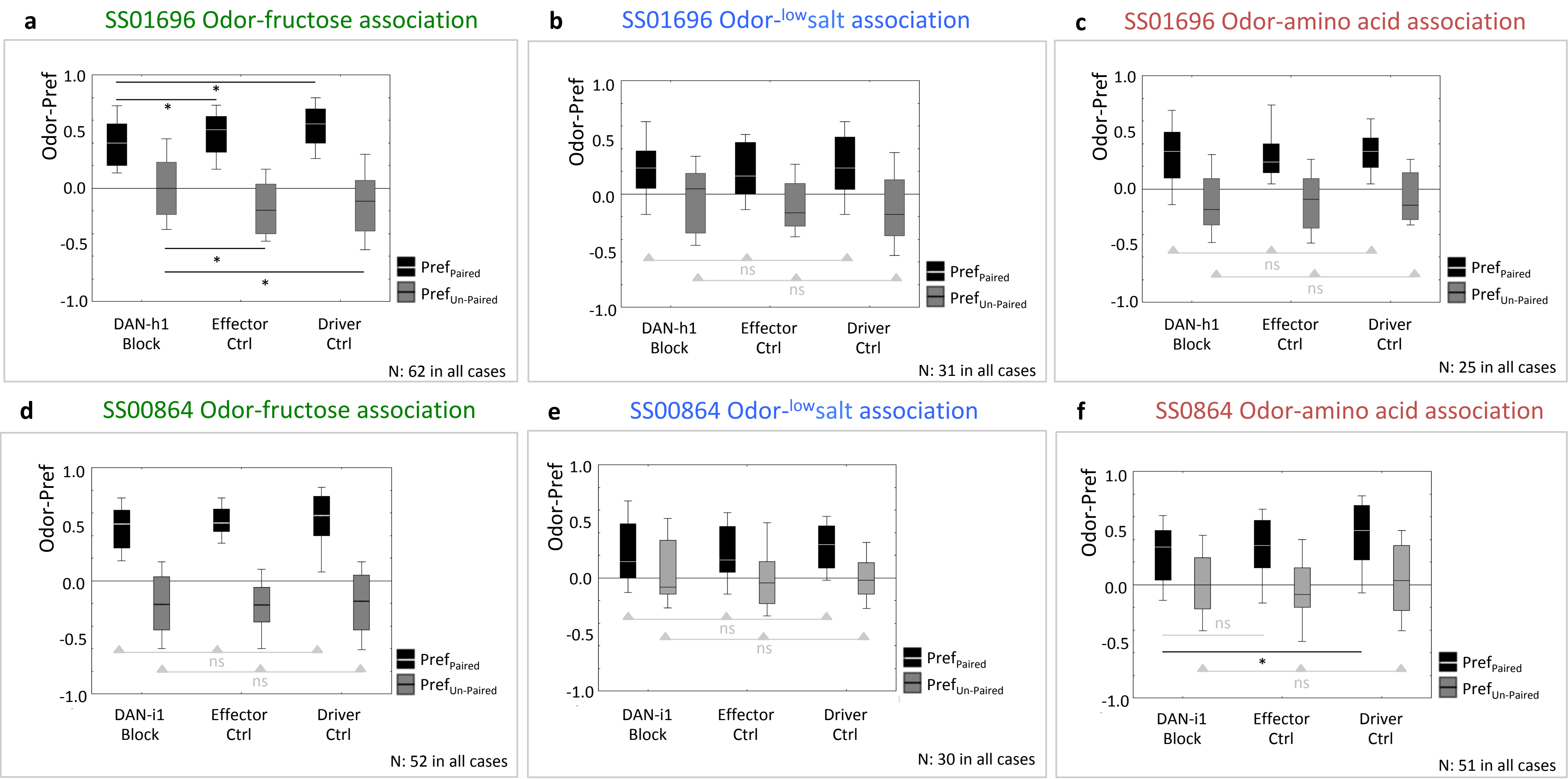

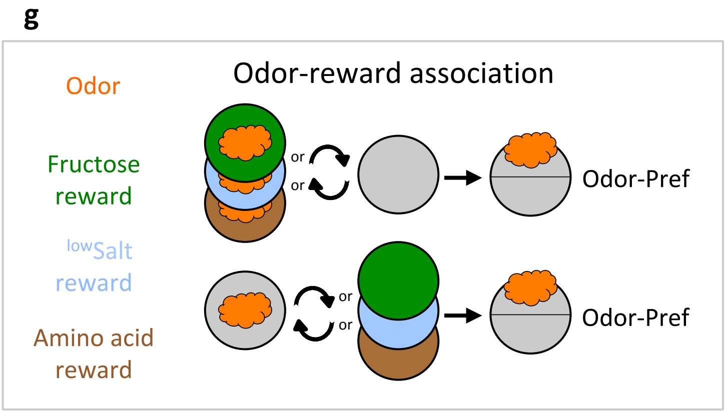

Supplementary Figure 14: Preference scores upon silencing DAN-h1 or DAN-i1, compared between taste rewards

(a-c) Preference scores for the trained odor *n*-amylacetate (Odor-Pref) underlying the associative performance indices (PI) displayed in Figure 8a, regarding the silencing of DAN-h1.

(d-f) Preference scores for the trained odor *n*-amylacetate (Odor-Pref) underlying the associative performance indices (PI) displayed in Figure 8b, regarding the silencing of DAN-i1.

(g) Schematic of the training paradigm (details as in Figure 8c).

Sample sizes are indicated within the figure. * refers to $P < 0.05/2$ in Mann-Whitney U-tests; ns refers to $P > 0.05$ in Kruskal-Wallis tests (except the top case in f where it refers to $P > 0.05/2$ in a Mann-Whitney U-test).

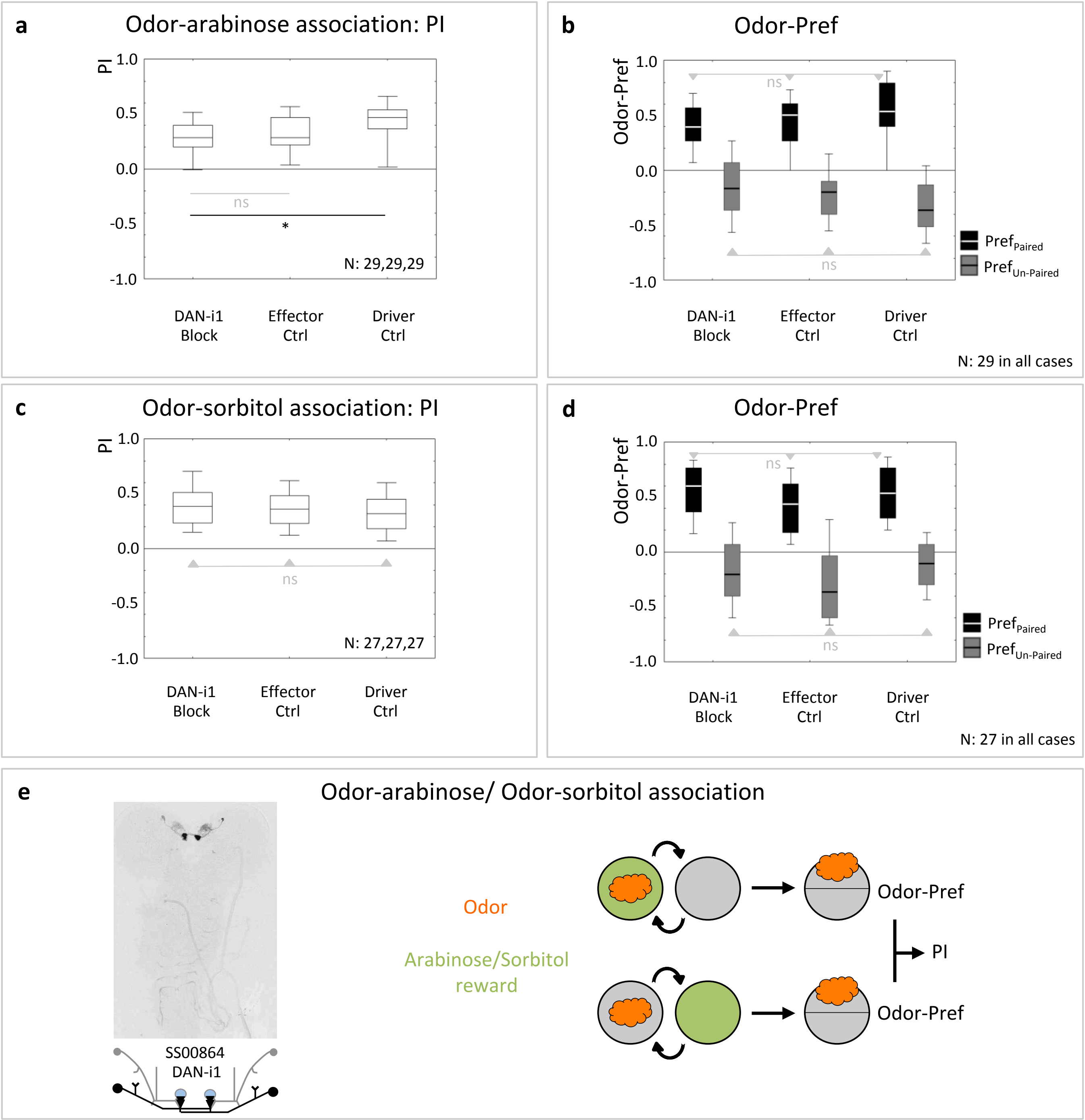

Supplementary Figure 15: Silencing DAN-i1 impairs neither odor-arabinose nor odor-sorbitol association

(a, b) For a split-Gal4 strain covering the MBIN of the upper toe (DAN-i1; SS00864), the panels show the associative performance indices measuring odor-arabinose reward memory (a), and the preference indices underlying these associative performance indices (b) upon silencing DAN-i1 by means of *Kir2.1::GFP* expression.

(c, d) Same as in (a, b), for odor-sorbitol reward association.

(e) Expression pattern of the split-Gal4 strain and a schematic overview of DAN-i1 (left), and schematic of the training paradigm (right; details as in Figure 3e).

Sample sizes are indicated within the figure. In (a) * refers to P< 0.05/2 and ns to P> 0.05/2 in Mann-Whitney U-tests. In all other cases ns refers to P> 0.05 in Kruskal-Wallis tests.

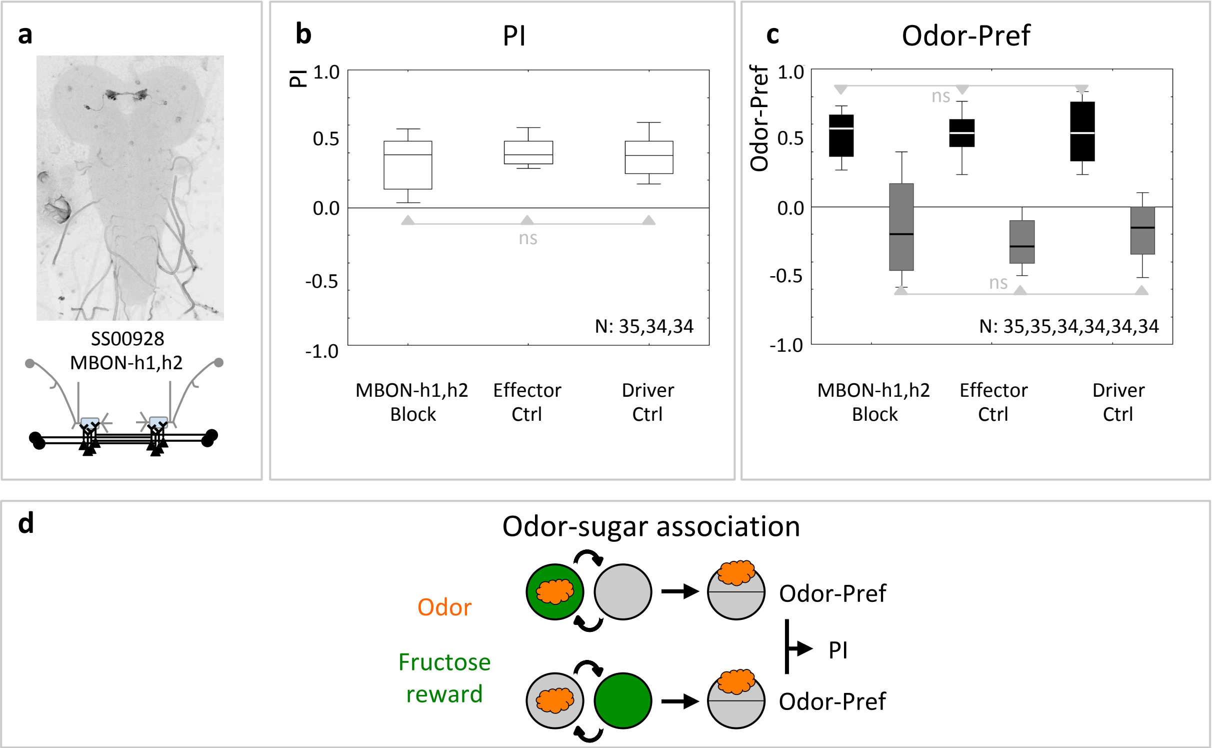

Supplementary Figure 16: Silencing MBON-h1,h2 does not affect odor-fructose association - revisited

(a-c) For an additional split-Gal4 strain covering the medial lobe shaft MBON double pair (MBON-h1,h2; SS00928), the panels show the expression pattern of the strain and a schematic overview of MBON-h1,h2 (a), the associative performance indices measuring odor-fructose reward memory (b), and the preference indices underlying these associative performance indices (c) upon silencing MBON-h1,h2 by means of *Kir2.1::GFP* expression.

(d) Schematic of the training paradigm (details as in Figure 3e).

Sample sizes are indicated within the figure. ns refers to P> 0.05 in Kruskal-Wallis tests.

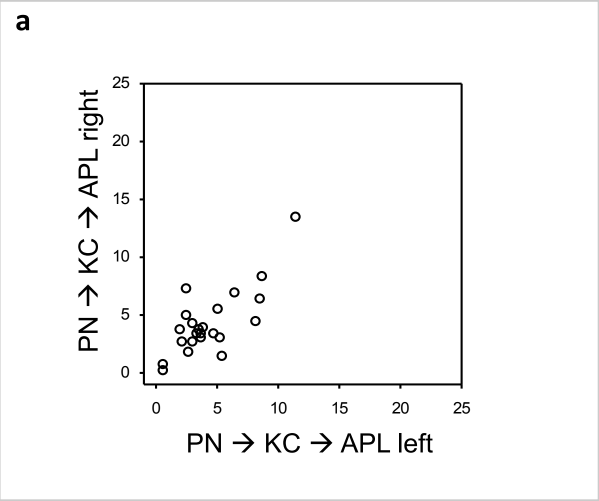

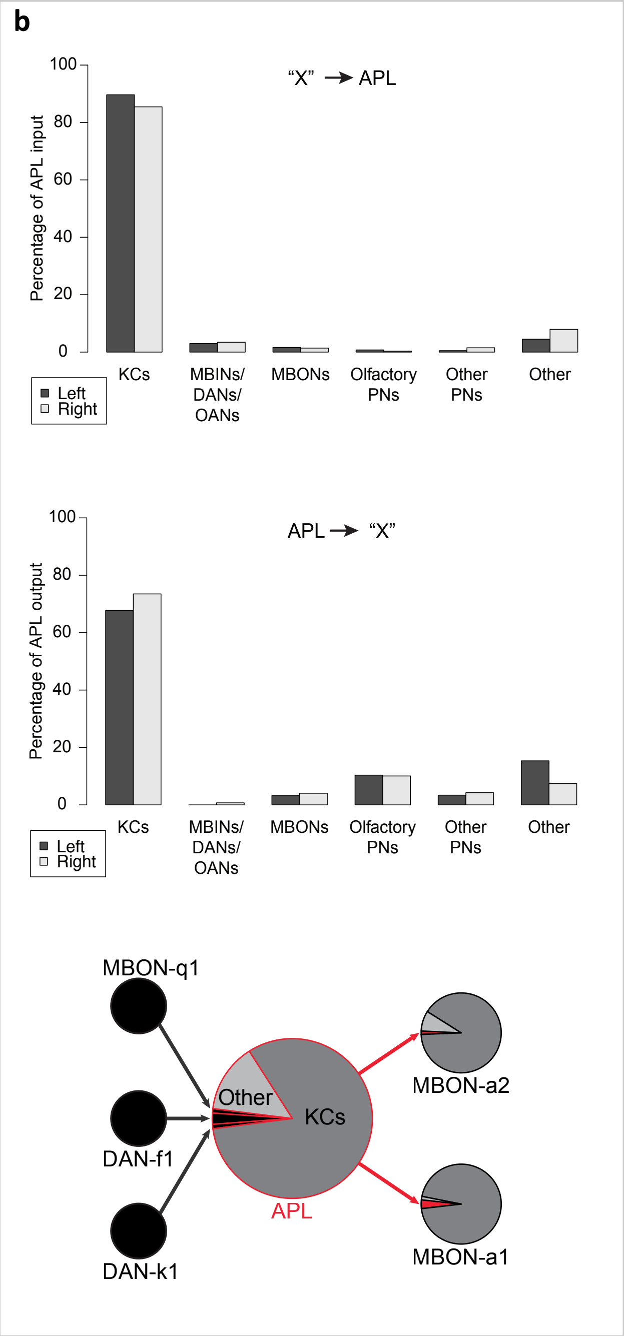

Supplementary Figure 17: Connectivity of the APL neuron
(a) The correlation of the PN-KC-APL matrix product shows that the right- and the left-hemisphere APL neuron sample the KC coding space in the same way, and in proportion to the fraction of PN-KC synapses that the respective PN class contributes (which is correlated between right and left hemisphere; not shown). The same correlation is found for the DAN-i1 neuron (Supplementary Fig. 13c). Correlations were determined with a Spearman rank correlation analysis at $P < 0.05$.
(b) Connectivity of the APL neuron with non-KCs. Fraction of the APL neurons' presynaptic partners as percent of the total amount of synaptic input to APL (top), and fraction of APLs' postsynaptic partners as percent of the total amount of synaptic output from APL (middle). Data are shown for the left (black) and right brain hemisphere (grey) separately. KCs contribute more than two thirds of the inputs to the APL neuron, which in turn dedicates about two thirds of its output to KCs. The bottom panel shows the connectivity of the APL neuron with DANs and MBONs. APL receives input from one vertical lobe DAN (DAN-f1) and one medial lobe DAN (DAN-k1) as well as from a vertical lobe MBON (MBON-q1, which was only found in stage 1 larvae: Figure S1 and Figure S2). The APL neuron delivers output to both calyx MBONs (MBON-a1 and MBON-a2). Indicated connections are axo-dendritic and shown as fractions of inputs onto the receiving neuron.

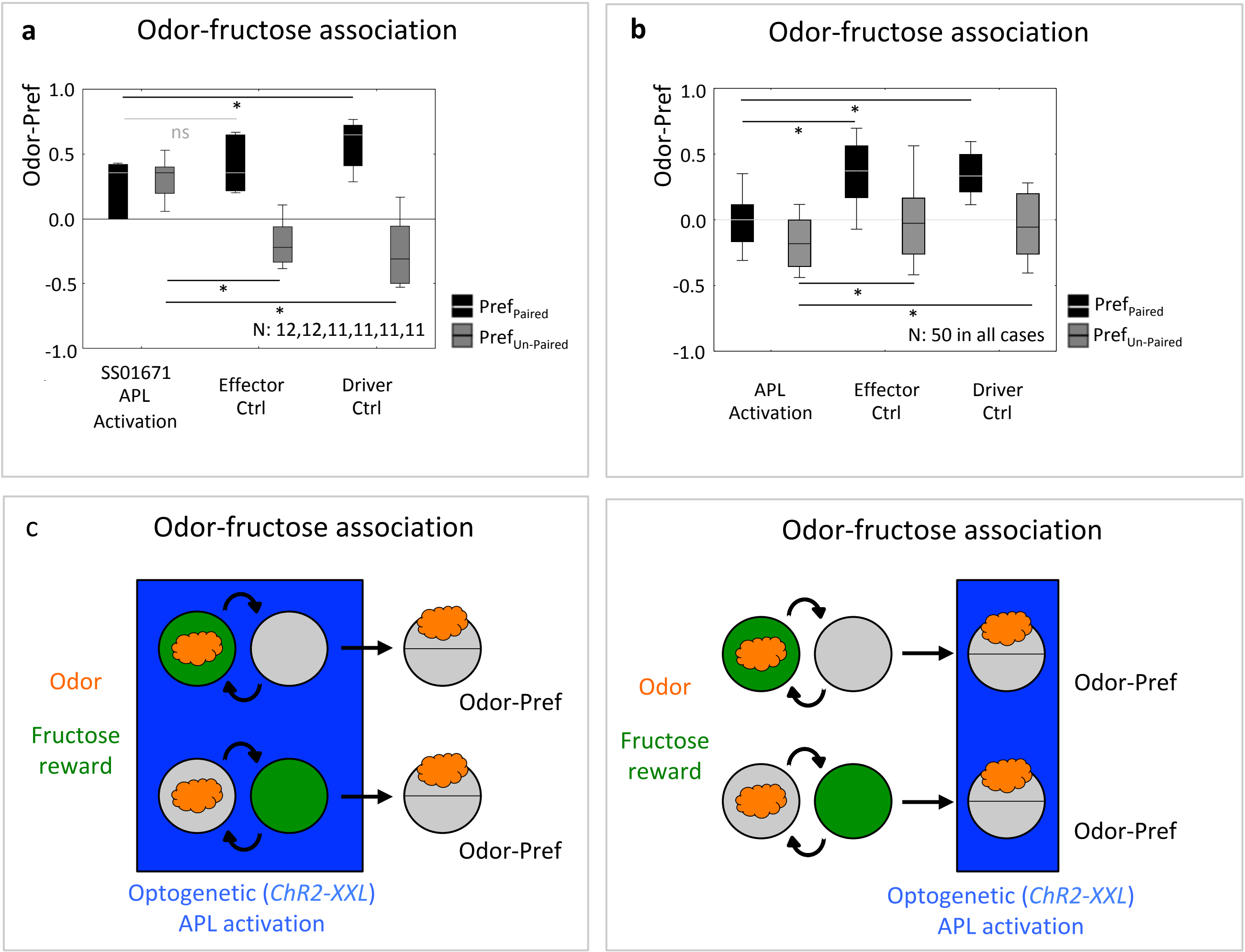

Supplementary Figure 18: Preference scores upon optogenetically activating the APL neuron during training only or during testing only

(a) Preference scores for the trained odor *n*-amylacetate (Odor-Pref) underlying the associative performance indices (PI) displayed in Figure 10c, regarding the optogenetic activation of the APL neuron during training only.

(b) Same as in (a), but for optogenetic activation of the APL neuron during testing only; data relate to the PI scores in Figure 10e.

(c and d) Schematics of the behavioral paradigms (details as in Figure 10d and f).

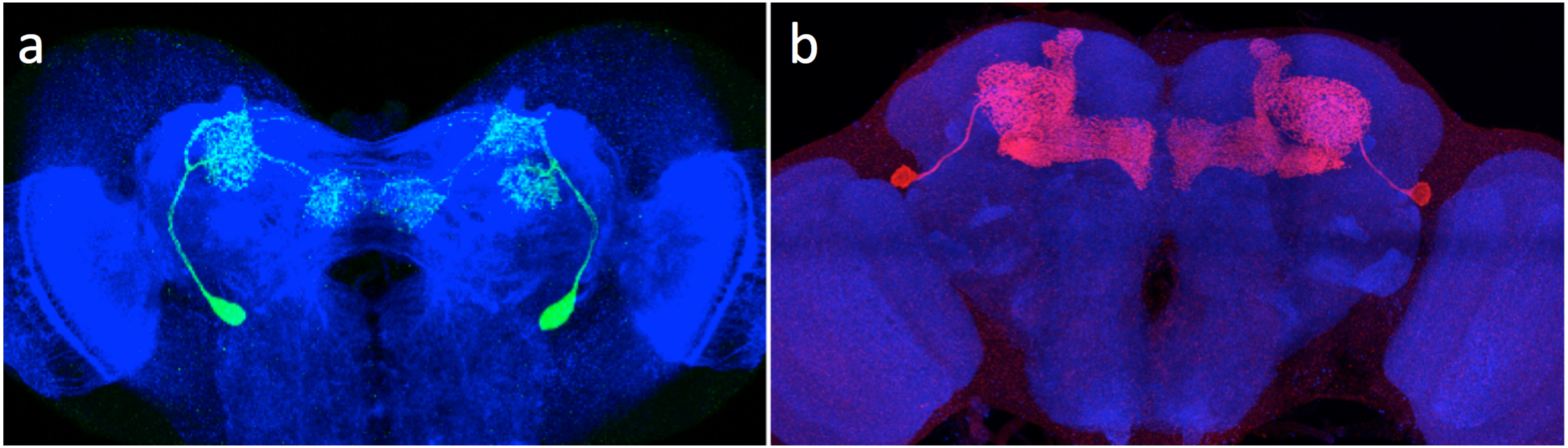

Supplementary Figure 19: The APL neuron is remodeled and persists through metamorphosis

(a) The APL neuron in the larva, revealed by anti-GFP staining (green) in offspring of a split-GAL4 driver strain expressing only in the APL neuron (GMR_SS01671) crossed to *UAS-GFP* as the effector strain. Neuropil structure is revealed using anti- N-cadherin (blue).

(b) Transgene expression in the APL neuron was induced late in larval life and then maintained into adulthood using a technique based on (5). A conditional flippase (hPRFlp) is expressed from the GMR_SS01671 split-Gal4 driver. The flippase is activated only late in the last larval stage by feeding larvae the progesterone mimic mifepristone (RU-486). The activated flippase removes a "stop" cassette from an actin-LexA driver, resulting in the constitutive expression of the tomato-RFP variant into the adult stage. To achieve this for the APL neuron, GMR_SS01671 was crossed to the three-transgene strain pJFRC48-13XLexAop2-IVS-myrtdTomato; Actin5Cp4.6>dsFRT>LexAp65; pJFRC108-20XUAS-IVS-hPRFlp-p10 respectively located to the attP8, attP5 and VK00005/ TM6 landing sites (available upon request). The image shows staining using anti-RFP (red); neuropil structure is revealed using anti- N-cadherin (blue).

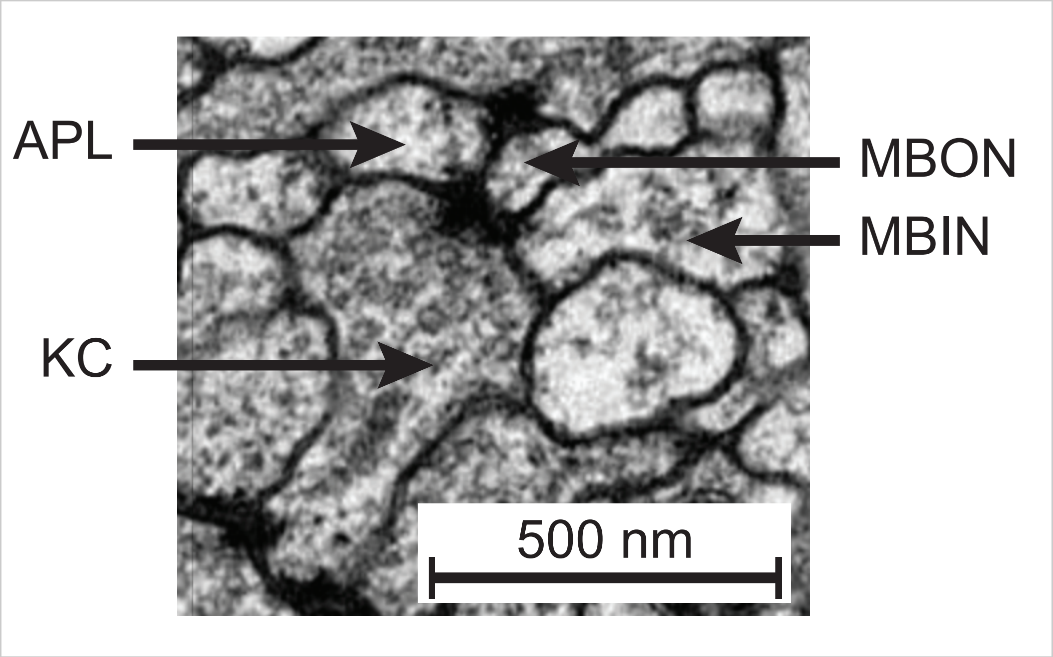

Supplementary Figure 20: Example of a polyadic complex with KCs, MBINs, MBONs, and the APL neuron
Electron micrograph of a polyadic synaptic complex of a KC with the APL neuron, an MBIN and an MBON.

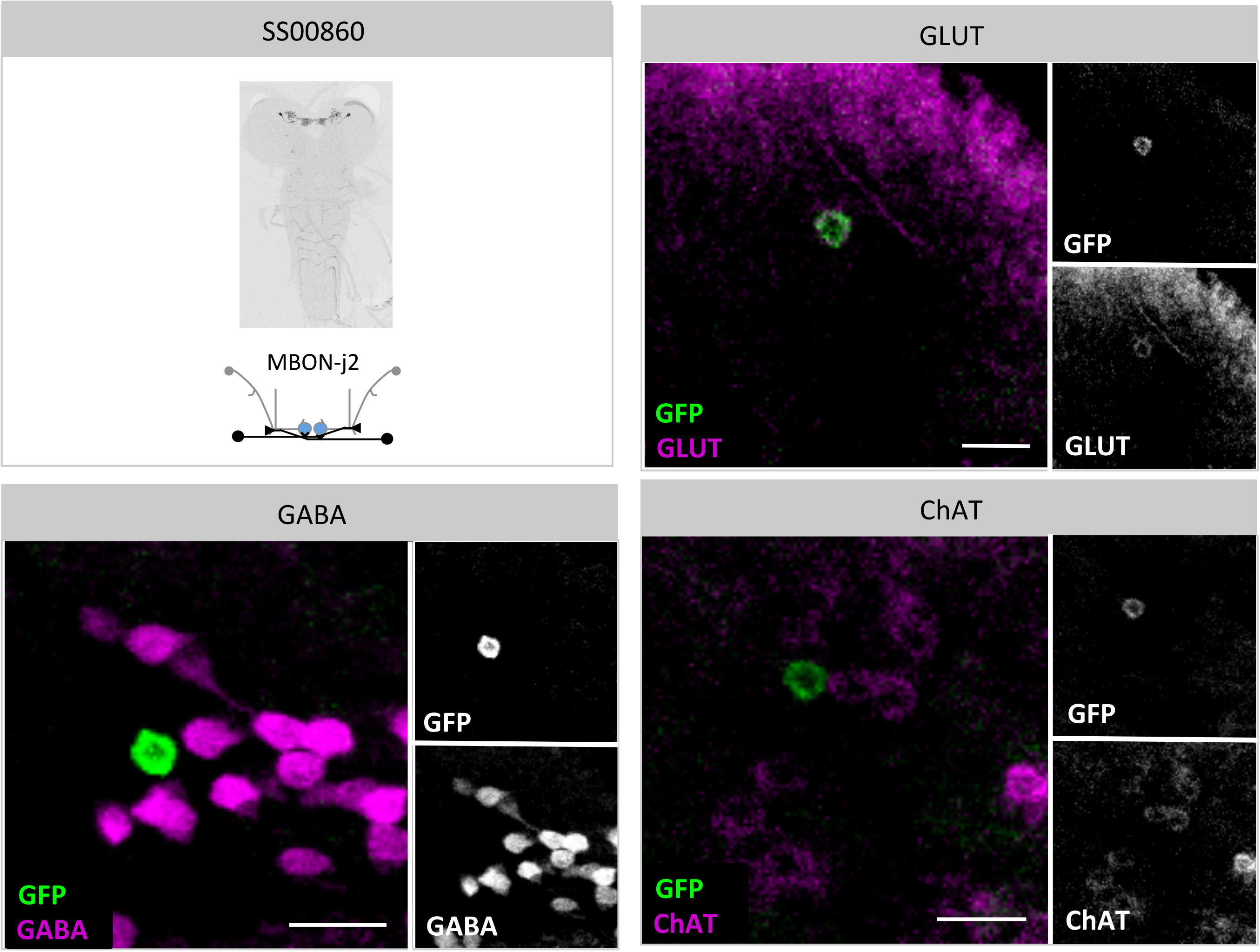

Supplementary Figure 21: MBON-j2 is glutamatergic

Testing for the neurotransmitter properties of MBON-j2 by immunohistochemistry. At the top left the expression pattern of the SS00860 split-Gal4 driver strain and a schematic of MBON-j2 is shown. In addition, double labeling of MBON-j2 by anti-GFP in green with staining respectively for anti-GLUT (GLUT: vesicular glutamate transporter; top right), anti-GABA (GABA: gamma aminobutyric acid; bottom left), and anti-ChAT (ChAT: choline acetyltransferase; bottom right) is shown in magenta. Insets show the respective staining separately in black and white. MBON-j2 only stains for anti-GLUT and is therefore regarded as glutamatergic. Scale bars: 5 µm.

Supplementary References

1 Eichler, K., Li, F., Litwin-Kumar, A., Park, Y., Andrade, I., Schneider-Mizell, C. M., Saumweber, T., Huser, A., Eschbach, C., Gerber, B., Fetter, R. D., Truman, J. W., Priebe, C. E., Abbott, L. F., Thum, A. S., Zlatic, M., and Cardona, A., The complete connectome of a learning and memory centre in an insect brain. *Nature* **548** (7666), 175-182 (2017).

2 Li, H. H., Kroll, J. R., Lennox, S. M., Ogundeyi, O., Jeter, J., Depasquale, G., and Truman, J. W., A GAL4 driver resource for developmental and behavioral studies on the larval CNS of Drosophila. *Cell Rep* **8** (3), 897-908 (2014).

3 Michels, B., Diegelmann, S., Tanimoto, H., Schwenkert, I., Buchner, E., and Gerber, B., A role for Synapsin in associative learning: the Drosophila larva as a study case. *Learn Mem* **12** (3), 224-231 (2005).

4 Saumweber, T., Weyhersmüller, A., Hallermann, S., Diegelmann, S., Michels, B., Bucher, D., Funk, N., Reisch, D., Krohne, G., Wegener, S., Buchner, E., and Gerber, B., Behavioral and synaptic plasticity are impaired upon lack of the synaptic protein SAP47. *J Neurosci* **31** (9), 3508-3518 (2011).

5 Harris, R. M., Pfeiffer, B. D., Rubin, G. M., and Truman, J. W., Neuron hemilineages provide the functional ground plan for the Drosophila ventral nervous system. *Elife* **4** (2015).
