# Supplementary material for: Functional architecture of reward learning in mushroom body extrinsic neurons of larval Drosophila
Source: Nat Commun. 2018 Mar 16;9:1104. doi: 10.1038/s41467-018-03130-1 (PMC5856778; doi:10.1038/s41467-018-03130-1)
Supplement: Supplementary file 2 — Description of Additional Supplementary Files(PDF 76 kb) [file 41467_2018_3130_MOESM2_ESM.pdf]

Description of Additional Supplementary Files

File Name: Supplementary Movie 1
Description: Body and nervous system of a 3rd instar Drosophila larva.

File Name: Supplementary Movie 2
Description: 3D view of the body and nervous system of a 3rd instar Drosophila larva, with zoom on motor neuron terminals.

File Name: Supplementary Movie 3
Description: Schematic body plan (top; from Demerec and Kaufmann, 1940) and indication of orientation using a 3D print from the file in Movie 1 (bottom), followed by a series of optical sections from ventral to dorsal, and back.

File Name: Supplementary Movie 4
Description: Indication of orientation using a 3D print from the file in Movie 1, followed by a series of optical sections from anterior to posterior, and back.

File Name: Supplementary Movie 5
Description: Indication of orientation using a 3D print from the file in Movie 1, followed by a series of optical sections from left to right, and back.

File Name: Supplementary Movie 6
Description: Visualization of APL connectivity within the mushroom body (left) and of all MBINs, PNs, KCs, and MBONs (right) using a standard force-directed algorithm (Fruchtermann and Reingold 1991). Based on the connectivity data available in Eichler et al. (2017).

File Name: Supplementary Movie 7
Description: Visualization of DAN-i1 connectivity within the mushroom body (left) and of all MBINs, PNs, KCs, and MBONs (right) using a standard force-directed algorithm (Fruchtermann and Reingold 1991). Based on the connectivity data available in Eichler et al. (2017).

File Name: Supplementary Data 1
Description: Documents the behavioral data presented in the Figures and Supplementary Figures.
